# How does biomass distribution change with size and differ among species? An analysis for 1200 plant species from five continents

**DOI:** 10.1111/nph.13571

**Published:** 2015-07-22

**Authors:** Hendrik Poorter, Andrzej M. Jagodzinski, Ricardo Ruiz‐Peinado, Shem Kuyah, Yunjian Luo, Jacek Oleksyn, Vladimir A. Usoltsev, Thomas N. Buckley, Peter B. Reich, Lawren Sack

**Affiliations:** ^1^ Plant Sciences (IBG‐2)Forschungszentrum Jülich GmbHD‐52425JülichGermany; ^2^ Polish Academy of SciencesInstitute of DendrologyParkowa 5KornikPL‐62‐035Poland; ^3^ Department of Game Management and Forest ProtectionFaculty of ForestryPoznan University of Life SciencesWojska Polskiego 71cPoznanPL‐60‐625Poland; ^4^ Departamento de Selvicultura y Gestión de Sistemas ForestalesINIA‐CIFORAvda. A Coruña, km 7.5.Madrid28040Spain; ^5^ Sustainable Forest Management Research InstituteUniversity of Valladolid‐INIAMadridSpain; ^6^ Jomo Kenyatta University of Agriculture and Technology (JKUAT)PO Box 62000Nairobi00200Kenya; ^7^ Department of EcologySchool of Horticulture and Plant ProtectionYangzhou University48 Wenhui East RoadYangzhou225009China; ^8^ State Key Laboratory of Urban and Regional EcologyResearch Centre for Eco‐Environmental SciencesChinese Academy of Sciences18 Shuangqing RoadHaidian DistrictBeijing100085China; ^9^ Department of Forest ResourcesUniversity of Minnesota1530 Cleveland Ave NSt PaulMN55108USA; ^10^ Ural State Forest Engineering UniversitySibirskiy Trakt 37Ekaterinburg620100Russia; ^11^ Botanical Garden of Ural Branch of Russian Academy of Sciencesul. Vos'mogo Marta 202aEkaterinburg620144Russia; ^12^ IA Watson Grains Research CentreFaculty of Agriculture and EnvironmentThe University of Sydney12656 Newell HighwayNarrabriNSWAustralia; ^13^ Hawkesbury Institute for the EnvironmentUniversity of Western SydneyLocked Bag 1797PenrithNSW2751Australia; ^14^ Department of Ecology and EvolutionUniversity of California Los Angeles621 Charles E. Young Drive SouthLos AngelesCA90095USA

**Keywords:** allometry, biomass allocation, biomass distribution, leaf mass fraction (LMF), leaf weight ratio, metabolic scaling theory, shoot : root ratio

## Abstract

- We compiled a global database for leaf, stem and root biomass representing *c*. 11 000 records for *c*. 1200 herbaceous and woody species grown under either controlled or field conditions. We used this data set to analyse allometric relationships and fractional biomass distribution to leaves, stems and roots.
- We tested whether allometric scaling exponents are generally constant across plant sizes as predicted by metabolic scaling theory, or whether instead they change dynamically with plant size. We also quantified interspecific variation in biomass distribution among plant families and functional groups.
- Across all species combined, leaf vs stem and leaf vs root scaling exponents decreased from *c*. 1.00 for small plants to *c*. 0.60 for the largest trees considered. Evergreens had substantially higher leaf mass fractions (LMFs) than deciduous species, whereas graminoids maintained higher root mass fractions (RMFs) than eudicotyledonous herbs.
- These patterns do not support the hypothesis of fixed allometric exponents. Rather, continuous shifts in allometric exponents with plant size during ontogeny and evolution are the norm. Across seed plants, variation in biomass distribution among species is related more to function than phylogeny. We propose that the higher LMF of evergreens at least partly compensates for their relatively low leaf area : leaf mass ratio.

## Introduction

A plant's organs serve multiple distinct functions. For example, leaves provide sugars, stems and branches position the leaves in an advantageous light environment and transport water as well as nutrients, and roots acquire water and nutrients and anchor the plant. For a species to achieve optimal performance at the whole‐plant level, there has to be a certain proportionality among these functions, as all are essential for growth and reproduction. This proportionality depends in part on the relative amounts of mass present in these organs. Although various terminology has been used (Reich, 2002), the generic term we will use throughout this paper to describe how the biomass of one organ relates to that of another or of the whole is ‘biomass distribution’. Note that this should not be confused with dynamic allocation of newly fixed photosynthates to different organ systems, as the realized biomass distribution at any moment is the cumulative result of dynamic carbon (C) allocation over time and loss rates of mass among organs throughout its life. In this study, we focus on the relationship of biomass distribution to plant size and its variation among species.

Biomass distribution has been studied using two basic approaches. The first approach employs an allometric analysis. It focuses on how the absolute size of an organ (or its physiological rate) relates to the total size of the organism or another organ, as these sizes or rates may change during development or across species. These relationships are often well described by a power law of the form:

(Eqn 1) $$Y=aX^{b}$$

where *X* could be, for example, the mass of an individual of a given species, and *Y* the mass of a specific organ. Parameter *a* is the ‘allometric constant’ and *b* the ‘scaling exponent’. Early researchers including Snell (1892) and Dubois (1897) observed that across species the relationship between brain mass and whole‐organism mass was characterized by a scaling exponent of ⅔. Pearsall (1927) applied Eqn 1 to analyse relationships between different plant organs during development, and showed the scaling exponent *b* to be mathematically equivalent to the ratio of the relative growth rates of organs *X* and *Y* (Huxley, 1932). Further work showed strong allometric trends in animals for metabolic rate (e.g. whole‐organism respiration rate) against body mass, with an apparently stable scaling exponent of ¾ (Kleiber, 1932; but see Makarieva *et al*., 2008). Although correlation coefficients or *r* ^2^ values were not commonly reported in the time of Kleiber, it was already obvious that Eqn 1 explained a great deal of the variation in the biological traits considered.

West *et al*. (1997) proposed an intriguing biological model unifying allometric observations in plants and animals in what is now called ‘metabolic scaling theory’ (MST; see Table 1 for an explanation of the acronyms used). This theory suggested a central role for the vascular transport system of water in the case of plants and of blood or air in the case of animals. It involved a number of assumptions, of which the optimization of the fractal‐like design of the vascular transport system is the most important, and predicted constant scaling exponents (such as ¾ for metabolic rate vs size) across large ranges of plant size, often with quarter‐powers. Niklas, Enquist and co‐workers further developed the MST to include relationships between plant parts in across‐species comparisons, again predicting fixed exponents with quarter‐powers (e.g. Enquist & Niklas, 2002a; Niklas, 2004; McCarthy *et al*., 2007). Combining MST with a number of assumptions regarding the lengths, diameters and mass densities of stems and roots, these authors predicted that the scaling of leaf vs stem, leaf vs root and stem vs root mass would follow constant scaling exponents of ¾, ¾ and 1.0, respectively (Enquist & Niklas, 2002a). We will refer to this model as the MST1 model. The predicted relationships were apparently supported by the high *r* ^2^ of fixed power laws fitted to compiled data sets of *c*. 400–700 records (the number depending on the publication), combining data for *c*. 250–300 vascular plant species ranging from small herbs grown in the laboratory to adult trees from various forests and plantations around the world (Enquist & Niklas, 2002a; Niklas, 2004). Niklas & Enquist (2002) therefore concluded that ‘a single biomass allocation pattern for leaf stem and root construction appears to hold sway across all extant seed plants.’

**Table 1:** List of abbreviations of concepts and variables used here

Abbreviation	Full name	Elucidation	Units
MST	Metabolic scaling theory	A model explaining scaling relationships between biological variables among (groups of) plants or animals	
FEM	Functional equilibrium model	The concept that plants invest relatively more biomass in the organ that limits growth most	
LMF	Leaf mass fraction	Leaf dry mass/total plant dry mass	g g^−1^
SMF	Stem mass fraction	Stem dry mass/total plant dry mass	g g^−1^
RMF	Root mass fraction	Root dry mass/total plant dry mass	g g^−1^
pLMF	Percentile rank of LMF	The percentile rank of an LMF observation relative to all data in the database, after correction for size‐related differences	%
pSMF	Percentile rank of SMF	As pLMF, but for an SMF observation	%
pRMF	Percentile rank of RMF	As pLMF, but for a RMF observation	%
SLA	Specific leaf area	Leaf area/leaf dry mass	m^2^ kg^−1^
LAI	Leaf area index	Total leaf area/total ground area	m^2^ m^−2^

In a deviation from the MST1 model, Enquist & Niklas (2002a,b), Niklas (2004) and Enquist *et al*. (2007) suggested that ¾ scaling does not apply to ‘small’ plants, and that isometric scaling was expected for such plants (i.e. *b *=* *1.0). Various biological reasons were proposed for this change, including a disappearing effect of seed mass (Enquist & Niklas, 2002a,b), stem photosynthesis being only present in young plants (Enquist & Niklas, 2002b), the onset of secondary thickening in plants older than 1 yr (Niklas, 2004) and changes in gravity and volume‐filling architecture with age (Enquist *et al*., 2007), although these effects were not verified. In their series of papers, these authors argued for a binary contrast with an abrupt shift of the scaling exponent from 1.0 to ¾, at a transition point that was variously defined as 1 yr of age (Enquist & Niklas, 2002a), or of 1 g (Enquist *et al*., 2007) or 64 g of total dry mass (Niklas, 2004). We refer to this model as the MST2 model.

A second way to analyse biomass distribution is to express the biomass of individual organs as a fraction or proportion of the total organismal biomass present at a given time (leaf mass fraction (LMF); stem mass fraction (SMF); root mass fraction (RMF)). These proportional biomass distribution patterns have been used to analyse responses of given genotypes to a range of environmental conditions, to examine ontogenetic trends over time and to compare performance across species, and they are fundamental to models that analyse growth rates of plants to reveal the underlying components (e.g. Evans, 1972; Poorter *et al*., 2013). We will refer to this method as ‘clasmometry’ (measuring fractions) to distinguish it from allometry. Clasmometry is simpler than allometric analyses because biomass fractions can be computed directly for each plant, which avoids the assumptions inherent in fitting Eqn 1 to data for all plants combined. Although the resulting biomass fractions do not account directly for variation in body size (Packard & Boardman, 1988), it is straightforward to do so, by plotting biomass fractions against plant size, in effect achieving a similar goal as in an allometric analysis of biomass distribution (Poorter & Sack, 2012). Especially for trees, where individuals may grow through 10 orders of biomass, size is thought to be an important determinant of biomass fractions, as proportionally more biomass has to be invested in support tissue as plants grow larger (Coleman *et al*., 1994). Biomass fractions are often interpreted in terms of the functional equilibrium model (FEM; sometimes referred to as optimal partitioning theory), which states that plants change the proportion of leaves, stems and roots depending on the relative limitations of light, CO_2_, water and nutrients on the physiological activity of the various organs (Brouwer, 1963; Davidson, 1969; Bloom *et al*., 1985). The FEM principles have been explicitly represented within teleonomic (goal‐directed) models in which biomass distribution among organs is adjusted during growth such that growth rate is maximized (Thornley & Parsons, 2014). Although the FEM was specifically designed to explain the response of plants to their environment, it can also be applied to plants during ontogeny (Brouwer, 1963; Buckley & Roberts, 2006a), or to the comparison of species with different physiological activities (Buckley & Roberts, 2006b).

Most previous papers on biomass distribution focused on either allometric or clasmometric analyses, but not on both. However, these provide two essential and complementary perspectives on the same phenomenon (Poorter & Sack, 2012), and in this paper we take both approaches. We focus on two related questions. First, combining all data within and across species for a ‘general’ allometry, how does plant biomass distribution shift with increasing plant size? Coordinated changes of organ sizes are reflected in the value of the scaling exponents. If MST is correct and the scaling exponents are fixed over the entirety (MST1) or a large part (MST2) of the size trajectory, this implies that the development and evolution of plant form and function are remarkably constrained. A constant scaling exponent of ¾ for leaf vs stem and leaf vs root scaling, as predicted by MST, means that, for every 1.0% increase in stem and root biomass, leaf biomass will increase by 0.75% across the whole plant size range considered. According to that theory, the relative growth rates of leaves, stems and roots would remain strictly proportional both during development across plant size and during evolution across a wide range of lineages. An alternative hypothesis is that plants adjust their biomass distribution more flexibly, so that scaling exponents change dynamically with plant size to improve performance. For animals, variation in the scaling exponent *b* has been reported by Makarieva *et al*. (2008) and Kolokotrones *et al*. (2010), for example, who showed that, even for Kleiber's law (the well‐recognized relationship between metabolic rate and body size), *b* was not a constant, but varied with animal type and generally decreased with size. It is clear that with increasing size plants generally show continuously increasing SMF and decreasing LMF, and less pronounced changes in RMF (Poorter *et al*., 2012). A further objective of this study, therefore, was to determine how these shifts in biomass fractions during plant growth are reflected in the allometric scaling exponents.

Our second question is how does biomass distribution vary across species groups, independently of the overall trends with plant size? While biomass fractions might shift greatly with plant size, there is also large variation among species at a given plant mass, and our aim was to quantify the major patterns underlying that variation. Although this question has received much less attention in the plant literature so far, previous analyses have shown that woody gymnosperms invest relatively more in leaves and less in stems than woody angiosperms (Körner, 1994; McCarthy *et al*., 2007; Reich *et al*., 2014) and herbaceous eudicots have higher LMFs and lower RMFs than herbaceous monocots (Poorter *et al*., 2012). Hui *et al*. (2014) showed that, in Chinese forests, RMFs differed among families, with low values for Cupressaceae and high values for Ulmaceae. However, no previous study has made a systematic, phylogenetically ordinated analysis across the plant kingdom. As size has such a strong effect on biomass distribution patterns (Poorter *et al*., 2012), rather than considering the variation among species in their absolute values for biomass fractions *per se*, we determined the deviations of biomass fractions from the main size‐related trends. We subsequently used allometry to analyse more specifically which of the organ relationships are affected.

To answer these questions, we compiled a database of unprecedented size and generality, with > 11 000 records on leaf, stem and root dry mass for *c*. 1200 species from all five continents. We analysed the relationships between individual plant parts as well as differences in biomass fractions, and tested specifically whether allometric exponents are fixed in relation to plant size across ontogeny and evolution, or whether there is evidence for dynamic scaling, and how biomass fractions vary among lineages and functional groups, and which organ allometries can explain these differences.

## Materials and Methods

### Data collection

We compiled an extensive database of biomass values for leaves, stems, and roots for a broad range of species and conditions, for gymnosperm and angiosperm species grown in growth chambers and glasshouses, outside in an agricultural setting or under natural conditions. Data from experiments where plants were subjected to various environmental conditions were taken from the MetaPhenomics database (Poorter *et al*., 2010), and supplemented with a range of literature data focused on species comparisons (e.g. Swanborough & Westoby, 1996; Taylor *et al*., 2010) or from any other experiment we found in the literature where plants were grown and where leaf, stem, and root mass values were reported separately. We did not include genetically modified organisms or plants treated with herbicides, hormones, and/or heavy metals, which may have led to substantial deviations from typical physiology and biomass distribution. To avoid ambiguous interpretations of individual plant size, we excluded clonal plants for which the biomass of all ramets together was reported. Finally, because biomass distribution patterns in herbs can change strongly when plants become reproductive, we included herbaceous plants only in the vegetative stage. Because we were interested in the effect of size on biomass distribution, we included data from various harvests, if reported. For each genotype and species, we collected the mean values of leaf, stem, and root mass per harvest, where the number of plants harvested generally was in the range of three to eight individuals.

The data for field‐grown plants comprised mainly shrubs and trees, where individuals are more easily distinguishable than in herbaceous plants. This is important as we used individual plant size as a relevant variable in the analysis. Data were included from large data compilations from the Western scientific literature (Cannell, 1982), the Eastern European literature (Usol'tsev, 2013) and Chinese papers and reports (Luo *et al*., 2014). These literature compilations were supplemented with original data collected for a range of species from C‐accounting initiatives (Montero *et al*., 2005; Kuyah *et al*., 2013; A. M. Jagodzinski *et al*., unpublished) and governmental reports, as well as primary literature on field‐grown plants (e.g. Ovington & Olson, 1970). In cases where biomass data were provided per ground area rather than per tree, we calculated values for the average tree, using the reported tree density. Following Niklas & Enquist (2002), we ignored the reproductive biomass in these trees, which – if present – generally forms a relatively small fraction of total biomass (0.03–1.2%; Cannell, 1982). References for all publications from which data were taken are given in Supporting Information Table S1.

The various assumptions related to data quality and representativeness are discussed in Notes S1. Species names were checked with the Taxonomic Name Resolution Service (Boyle *et al*., 2013). In total, there are 11 217 records for 1207 species reported in 1366 papers or scientific reports. A full list of references is given in Table S1. The actual biomass data can be found in Table S2.

### Data analysis

Inspection of the allometric plots showed five out of the > 11 000 records to deviate strongly from the others. They were removed from the analysis. For the establishment of overall general trends all other data were included, such that the trajectories of variables with size combine intra‐ and interspecific variation. Allometric relationships were first analysed with standard major axis (SMA) lines fitted to log‐transformed data, as predicted by MST to explain the relationships. We subsequently fitted quadratic or cubic polynomials in stepwise Model 1 regression analysis and checked which model was best supported using the Bayesian information criterion and analysis of residuals. Two different approaches were used for assessment of the changes in slope with plant size. First, we calculated the derivative of the fitted polynomial equation. This is a somewhat rigid approach, always yielding a straight line in the case of a second‐degree polynomial. We also applied a procedure that allows more flexible relationships, first determining the slope over small intervals and subsequently smoothing the resulting data with a polynomial (Poorter, 1989). Hence, we divided all data into 50 size classes, based on total plant dry mass. For each size bin we determined the median value of log‐transformed leaf, stem, and root mass, and used these median mass values to calculate the slope (difference in biomass of organ A/difference in biomass of organ B) over each adjacent triplet using a central derivative kernel. That is, the slope over each three neighbouring classes was determined from the values of the left and right class and this value was assigned to all three class members. The procedure was then reiterated shifting the triplet one bin to the right. This eventually leads to three slope estimates per class, which were averaged, plotted as a function of total plant mass and smoothed by a Loess curve (Efron & Tibshirani, 1994). The median values and 95% confidence intervals (CIs) of this Loess curve were derived after a bootstrapping procedure with 20 000 repetitions (Efron & Tibshirani, 1994). Apart from increased flexibility, the advantage of this second approach is that the results are not constrained by the *a priori* choice of an equation to fit the data.

For the assessment of species‐specific deviations from the overall trends of biomass distribution with plant size, we again used the 50 size classes. All observations of LMF (or SMF or RMF) within a given size class were ranked and characterized by percentiles. In this way, we corrected for the overall effect of size on mass fractions. The use of percentiles was inspired by the fact that LMF values were more variable for small than large plants. All percentiles (indicated in this paper as pLMF, pSMF and pRMF) calculated for a given species across the 50 size bins were subsequently combined. For each species, the median percentile was then calculated and the significance of deviations from the overall median (50%) was tested using a *t*‐test.

All data were analysed using R software (R Core Team, 2014). Phylogenetic analyses were conducted with package Diversitree (FitzJohn, 2012) from the R software, using the phylogenetic tree published in Zanne *et al*. (2014). In the Diversitree package, only one value per species is used in the calculations, so we used the median response across all records for a given species for the analysis, as specified earlier. For this analysis we only considered those species for which at least four records were present in the database. Because *c*. 20% of the species in our data set were not covered by the phylogenetic tree used, we also examined whether there were systematic differences at the family level. For this analysis, we only considered those families that were represented by at least four species and at least four observations per species. Differences between (groups of) species were tested statistically by Welch's *t*‐test or ANOVA.

## Results

### Fixed or dynamic scaling exponents

**Figure 1:**
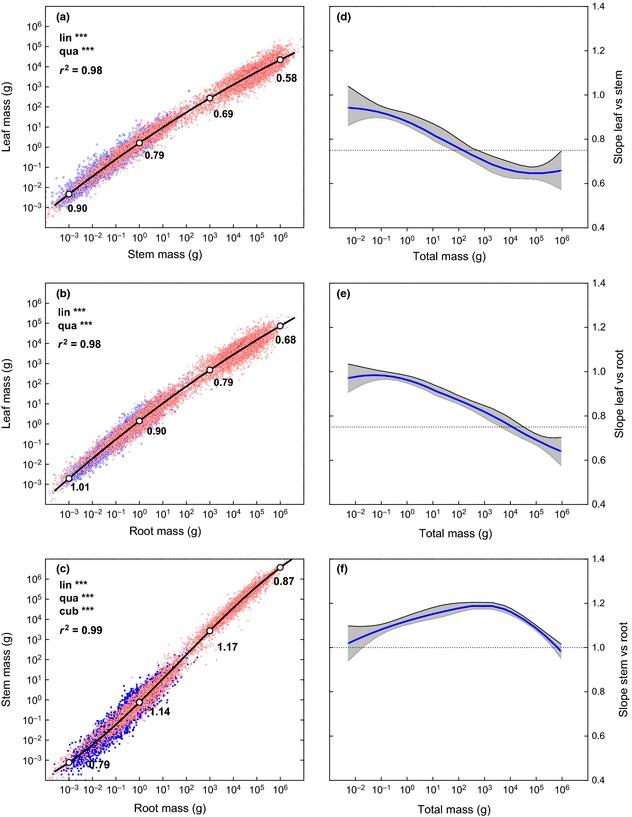
(a–c) The allometric relationship for (a) leaf vs stem mass; (b) leaf vs root mass; (c) stem vs root mass. Red and blue points represent data for woody (*n *=* *8170) and herbaceous (*n *=* *2960) species, respectively. The bold black lines show the overall fit of a quadratic (a, b) or cubic (c) regression. Numbers indicate the value for the slope of the line at the indicated white points. (d–f) The slope of the allometric relationship for (d) leaf vs stem mass; (e) leaf vs root mass; (f) stem vs root mass, all as a function of total plant dry mass. The bold lines indicate the Loess curve through the mean slope values, based on a bootstrap procedure with 20 000 repetitions. The shaded area indicates the 95% confidence interval of the slopes. The black dotted line indicates the value of (d, e) ¾ and (f) 1.0, as predicted by MST1 theory. lin, linear; qua, quadratic; cub, cubic.

Our database contained > 11 000 records, including *c*. 3000 for herbaceous species and *c*. 8200 for woody species, and representing *c*. 1200 species in total. More detail is given in Table S3. With plant dry mass varying from < 1 mg for 1‐wk‐old seedlings (e.g. *Erica cinerea*) to over 14 000 kg in > 100‐yr‐old trees (*Trilepsium madagascariensis*), the database included plant size over > 10 orders of magnitude. Straight lines fitted to the log‐transformed leaf vs stem, leaf vs root and stem vs root biomass data had *r* ^2^ values up to 0.988 (Table 2a; for separate regressions on herbaceous and woody species, see Table S4), although examination of residuals (Fig. S1) showed clear patterns that reject the use of log‐linear allometry to describe these data. Ignoring this problem, the value for the leaf vs stem scaling exponent was 0.74, very close to the ¾ predicted by MST, whereas both the leaf vs root (0.85) and stem vs root (1.15) scaling exponents were clearly and significantly (*P *<* *0.001) higher than those expected from the MST1 and MST2 models (post‐transition point). Statistical analysis showed that a quadratic curve was more appropriate to fit the data for leaf vs stem and leaf vs root scaling, whereas a cubic polynomial was more suitable for the stem vs root scaling. This was confirmed by stepwise regression (*P *<* *0.001 in all cases for the additional terms), by evaluation of the Bayesian information criterion (Table 2b), and by inspection of the residuals (Fig. S1) even though the increase in *r* ^2^ was small. These analyses clearly rejected the MST1 and MST2 models, which assumed single log‐linear relationship with fixed exponents, in favour of leaf vs stem, leaf vs root and stem vs root biomass allometries that shift continuously and substantially with plant size (Fig. 1a–c).

**Table 2:** Results of the fit for the allometric analysis

(a) Regression	a	b	95% CI for *b*	*r* ^2^
LM vs SM	0.113	0.740	0.738–0.742	0.978
LM vs RM	0.070	0.849	0.847–0.851	0.977
SM vs RM	−0.058	1.147	1.145–1.149	0.988

(b) Regression	*a*	*b*_1_	*b*_2_	*b*_3_	*r* ^2^	Δ BIC
LM vs SM	0.213	0.795	−0.0177	–	0.981	−1360
LM vs RM	0.151	0.897	−0.0184	–	0.979	−891
SM vs RM	−0.126	1.144	0.0318	−0.00606	0.989	−799

(a) Standard major axis regression (SMA; model 2 regression) for the intercept (*a*) and slope (*b*) of the regression of leaf mass (LM) vs stem mass (SM), LM vs root mass (RM), and SM vs RM, all based on log_10_‐transformed values. The 95% confidence interval for the slope and the *r* ^2^ of the equation are given. (b) Ordinary least square regression (OLS), with estimates for the linear (*b*_1_), quadratic (*b*_2_) and cubic (*b*_3_) coefficients. *a* is the value for the intercept, and Δ BIC shows the change in the value of the Bayesian information criterion as compared to a linear fit, for which the BIC was *c*. 5500 in all cases. The total number of observations was 11 217.

We determined the actual values of scaling exponents and how they changed with plant size in two ways: by calculating the derivative of the fitted polynomials and by smoothing locally determined slopes. The two approaches yielded similar conclusions: both leaf vs stem and leaf vs root scaling slopes were significantly higher than ¾ for plants smaller than 10 and 1000 g, respectively, and both slopes were significantly lower than ¾ for trees exceeding 10 and 100 kg (Fig. 1d,e). We thus found no indication of a constant scaling exponent across the size range considered. Our data contradicted the MST2 model both qualitatively, in that the shifting appeared continuous rather than discrete as predicted by MST2, and quantitatively, in that the exponents differed numerically from the values predicted by MST. While for young plants the scaling exponents involving leaf mass were close to 1.0, as shown by the 95% CI in Fig. 1(d,e), for large plants the exponents declined to values substantially below ¾. Stem vs root scaling did not comply with the MST2 model either. Although we found values close to 1.0 for very small and large plants, plants of intermediate size had a scaling exponent of up to 1.2 (Fig. 1f). Overall, there was only a small interval of plant size during which quarter‐power scaling or, for that matter, any single scaling coefficient was observed.

**Figure 2:**
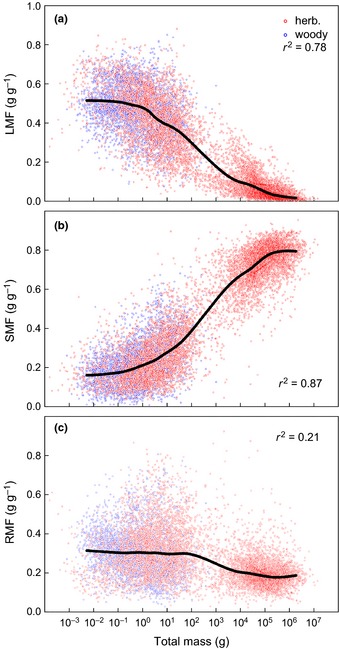
(a) Leaf mass fraction (LMF); (b) stem mass fraction (SMF) and (c) root mass fraction (RMF), plotted as a function of total plant dry mass. Red and blue points represent data for woody (*n *=* *8170) and herbaceous (*n *=* *2960) species, respectively. The bold line is a Loess curve fitted through the mean values of the 50 consecutive size classes that were discerned (see the Materials and Methods section for an explanation). The *r* ^2^ indicates how much of the overall variation in the data is explained by the Loess curve.

Thus, rather than a fixed allometry, we found small plants to show scaling coefficients of 1.0, whereas plants of intermediate sizes show disproportionate increase in stem biomass distribution, and very large plants increase stem and root mass in equal proportion. These changes were also clearly reflected in the clasmometric analysis. The overall trends in how biomass fractions changed with size, shown as lines in Fig. 2, indicate that, up to 100 g total plant mass, the median RMF remained remarkably stable, with roots representing *c*. 30% of total plant mass, but that LMF declined from *c*. 0.50 to *c*. 0.30 over that range, while SMF increased from *c*. 0.20 to *c*. 0.40. Above a size of 100 g, where almost all records in the data set pertain to woody plant species, the changes in biomass distribution are yet more pronounced: the RMF drops from *c*. 0.30 to *c*. 0.20, the average LMF decreases to 0.015 for very large trees, and SMF strongly increases up to *c*. 0.80. Above 1000 kg, stem and root mass fractions seem to stabilize.

### Differences in biomass distribution among lineages and functional groups

**Figure 3:**
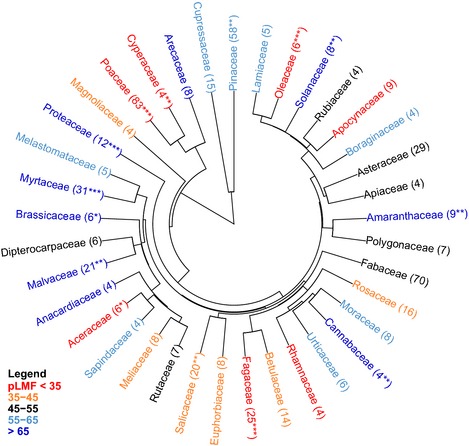
Phylogenetic tree of the leaf mass fraction (LMF) data at the family level. Data are based on the deviations of the LMF of each record from the median trend line as shown in Fig. 2(a), and are given as percentiles which are subsequently averaged per species (see the Materials and Methods section). Family names are colour‐coded depending on the median LMF ranking (pLMF) value considered over all species. Numbers behind the family name indicate the number of species on which the data are based. Families where the pLMF averaged over species deviates significantly from the overall median are indicated: *, *P *<* *0.05; **, *P *<* *0.01; ***, *P *<* *0.001.

Subsequently, we tested the extent to which phylogeny affected these biomass distribution patterns, focusing on the deviation for each point from the main trends in median mass fractions as measured by percentiles (pLMF, pSMF and pRMF). Species explained 55% and families 23% of the total variation in pLMF across all observations. For pLMF, the full phylogenetic tree at the species level is shown in Fig. S2, and a summary at the family level is given in Fig. 3. There is a clear phylogenetic signal in the gymnosperm families Pinaceae and, to a lesser extent, Cupressaceae, which have a higher LMF than average for their size. Further detail is shown in Table 3, where the observed ranges in pLMF, pSMF and pRMF are given for these families. Another clear contrast is that herbaceous graminoids (Cyperaceae and Poaceae) show relatively high fractions of biomass in roots compared with other monocots and eudicotyledonous herbs of similar size. Several eudicot families with large numbers of species also deviated significantly and consistently in biomass distribution. The most notable ones are listed in Table 3 and include the Fagaceae, Proteaceae and Solanaceae.

**Table 3:** The median percentile rank in leaf mass fraction (pLMF) per family averaged over all species measured for that family

Family	≥ 4 observations per species			≥ 1 observation per species		
	Median pLMF	No. of species	P	Median pLMF	No. of species	P
Cyperaceae	11	4	**	31	11	*
Oleaceae	16	6	***	35	12	ns
Aceraceae	25	6	*	31	9	*
Fagaceae	29	25	***	40	49	**
Poaceae	34	83	***	33	173	***
Salicaceae	37	20	**	39	30	**
Betulaceae	37	14	+	33	21	**
Asteraceae	59	29	+	60	64	*
Pinaceae	59	58	**	59	82	***
Malvaceae	66	21	**	58	32	ns
Moraceae	68	8	+	62	11	ns
Cupressaceae	68	15	*	61	20	+
Amaranthaceae	69	9	**	68	13	**
Arecaceae	70	8	+	54	13	ns
Myrtaceae	70	31	***	56	64	ns
Brassicaceae	72	6	*	77	8	**
Solanaceae	79	8	**	81	9	***
Cannabaceae	80	4	**	72	7	*
Proteaceae	81	12	***	79	18	***

pLMF values per species are considered over all size classes present in the database. The analysis was carried out with emphasis either on the quality of the estimate per species (at least four independent records available per species) or on the quantity of species (only one observation per species necessary for the species to be included). Data are most robust if they are consistent over the two approaches. *P*‐values are given for the probability that the averaged pLMF values deviate significantly from the median as derived by a *t*‐test. Listed are only those families with a significant deviation in this respect. ns, nonsignificant (*P *>* *0.10); +, 0.05 < *P *<* *0.10; *, *P *<* *0.05; **, *P *<* *0.01; ***, *P *<* *0.001).

**Figure 4:**
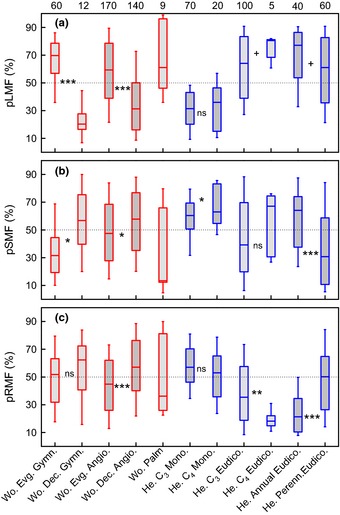
Boxplots indicating the distribution of (a) leaf mass fraction (pLMF) rankings as well as (b) stem mass fraction (pSMF) and (c) root mass fraction (pRMF) rankings for various functional groups. Red and blue boxes pertain to woody and herbaceous groups, respectively. The main box of the boxplots indicates the 25th and 75th percentiles, and the whiskers the 10th and 90th percentiles. The broken line shows the 50% value, which indicates no deviation from the mean trend. Woody palms were not included in any other woody group. Numbers at the top of (a) indicate the number of species on which each boxplot is based. Wo., woody; Evg., evergreen; Gymn., gymnosperms; Dec., deciduous; Angio., angiosperms; He., herbaceous; Mono, monocotyledons; Eudico., Eudicotyledons; Perenn., perennial. Significance values based on *t*‐tests for differences between adjacent groups are shown between the respective boxes. (ns, nonsignificant; +, 0.05 < *P *<* *0.10; *, *P *<* *0.05; **, *P *<* *0.01; ***, *P *<* *0.001).

Although we found broad phylogenetic patterning at the clade or family level, there was interesting variation below the family level as well. For example, in the case of the gymnosperms, which were found to have a relatively high LMF (Fig. 4a), the needle‐leaved deciduous species (*Larix*,* Metasequoia*, and *Taxodium*) had low values for pLMF relative to the needle‐leaved evergreen species (Fig. 4a; *P *<* *0.01). These differences were mirrored in pSMF (*P *<* *0.01), with far less divergence in pRMF. Contrasting biomass distribution patterns for deciduous vs evergreen species are also present within the angiosperms: evergreens had higher pLMF than deciduous species among the woody species of the basal angiosperms and the eudicots (Fig. 4a). In these lineages the higher pLMF of the evergreens corresponded to both a lower pSMF (*P *<* *0.05) and a lower pRMF (*P *<* *0.001) than deciduous species. The difference for the angiosperms was found for both the tropical/subtropical species (*P *<* *0.01) and for the temperate/boreal trees (*P *<* *0.05). Inspection of the deviations from the overall allometric plots among specific organs showed that differences among functional groups were attributable to modulation of the leaf vs stem scaling and leaf vs root scaling, but that the stem vs root scaling showed very little group differentiation (Fig. S3).

A third contrast we investigated was between herbaceous C_3_ and C_4_ species. C_4_ species are thought to have a superior photosynthetic rate on average than C_3_ species, and, based on the FEM framework, such a higher photosynthetic rate might be expected to drive preferential biomass distribution in the root system. However, no difference was observed between C_3_ and C_4_ species in pLMF or pRMF within the monocotyledonous clade (Fig. 4). For the eudicotyledonous clade, RMF was lower for the C_4_ species, but the number of C_4_ species in the analysis (five) is still very low. Clade was an important factor in the contrast between annual and perennial herbs. Whereas no overall difference was found within the monocotyledonous clade (data not shown), the eudicotyledonous annuals had higher investment in leaves and stems as compared with perennial species of the same size (Fig. 4). Further analysis by means of allometry showed that, in contrast to the woody species, functional herbaceous groups varied significantly in the stem vs root scaling exponent (Fig. S3).

## Discussion

### The implication of high *r* ^2^ in allometric relationships

Our database covered > 10 orders of magnitude in plant size, which is almost the full range of sizes of vascular plants in nature. Missing only is one additional order of magnitude for exceptionally large trees such as *Sequoiadendron*, which can reach 500 000 kg (Zinke & Stangenberger, 1994). All of the allometric relationships analysed showed very high *r* ^2^: whether we fitted linear or more complicated curves to the log–log data, all *r* ^2^ values exceeded 0.975. Given that ecological correlations often have *r* ^2^ values substantially lower than 0.50 (Møller & Jennions, 2002), scaling theory has therefore been considered to provide a ‘general’ biological law, which is quite an exceptional phenomenon in biology (Dhar & Giuliani, 2010). Further, the high *r* ^2^ values of log–log fits also led to the inference that fixed exponent allometric equations could explain biomass distribution to a large extent. For example, McCarthy *et al*. (2007) suggested that one could explain 97–99% of the variation in biomass distribution across the plant kingdom world‐wide if one could accurately determine the allometric constant and scaling exponent of Eqn 1. However, as emphasized in general by Nee *et al*. (2005) and exemplified in Notes S2 for the specific case of allometric relationships among organs, the *r* ^2^ value must be interpreted with care when the underlying data span orders of magnitude. This is because *r* ^2^ is mathematically determined to increase directly with the size range over which the variables are considered, regardless of the strength of the proportionality of the variables or the precise shape of the relationship. Thus, *r* ^2^ has very limited value for inferring the structure of relationships across scales. Indeed, even if the shoot and root system of plants were to show high variability in biomass distribution patterns, that is, if RMF values ranged between 0.01 and 0.99 (and thus, shoot to root ratios were confined between 0.01 and 99), the *r* ^2^ of the relationships between log‐transformed shoot and root biomasses would still be *c*. 0.94 or higher (Notes S2). Hence, the fact that an allometric equation ‘explains’ *c*. 97% of the variation in log‐transformed shoot and root mass according to its *r* ^2^ does not imply that the equation is highly informative, nor does it necessarily indicate a biological law that extends beyond the conclusion that plants or species with a larger root mass are highly likely to have larger shoot mass as well. Moreover, it follows that, in comparing linear vs curved allometric relationships, a high *r* ^2^ for the linear relationship does not necessarily imply that curvature is nonexistent or that it would add only marginal insight or predictive value. In short, the *r* ^2^ value of an allometric model spanning many orders of magnitude of size says little about the accuracy of the model's assumptions, and it does not automatically support a constant scaling exponent. As discussed later, the clasmometric approach is better suited for drawing inferences about the strength of relative proportionality of organs, because it eliminates the dependence on absolute scale that leads to spurious inflation of *r* ^2^ values in the allometric approach.

### The value of the scaling exponent

Ignoring at first the dynamic scaling relationships that were best supported by the data, and focusing on the log‐linear allometric relationships (Table 2), we found slopes to be very similar to those reported by Niklas (2004) for his data set with actual biomass observations. As our database contains 15‐fold more data than considered by Niklas (2004), we conclude that the calculated slopes are likely to be stable approximations for the log‐linear fits across all plants. As a result of our considerably higher degrees of freedom, however, we found much smaller CIs around the fitted coefficients. Consequently, none of the ¾ or 1.0 values forecasted by the MST models for the scaling exponent were within the 95% CIs, although in the case of leaf vs stem scaling the estimated slope was close to the predicted ¾. Thus, just as inclusion of ¾ in the 95% CI has previously been used to support the MST model, the much narrower CIs in our data set can be used to formally refute the model. More importantly, however, we found no indication that the scaling exponent was constant in any of the three relationships (Fig. 1; Table 2). We are the first – to our knowledge – to show this for an extended data set on plant organ mass. However, dynamic exponents have also been observed in other fields of biology: Kolokotrones *et al*. (2010), for example, found that for the relationship between metabolic rate and animal size the scaling exponent *b* decreased monotonically with size, and changing exponents were also reported in the scaling of tree respiration with tree size (Cheng *et al*., 2010).

What is the overall pattern of how these scaling exponents change with size, and how could this pattern be explained from a functional perspective? Our data set, like those of others (Enquist & Niklas, 2002a; Niklas, 2004), represents a mixture of younger and older plants. It therefore includes both comparisons across ontogenetic stages for individuals of given species, and comparisons among species, thus representing evolutionary shifts in biomass distribution across species of different size. This does not invalidate our data set as a test for MST, because the arguments about size‐related changes in organ structure and function used by MST to predict invariance of scaling exponent *b* across species (i.e. in the evolutionary domain) would apply at least as well within species (i.e. within the ontogenetic domain) as across species. Thus, a fixed scaling exponent of ¾, as suggested by MST1, would imply a completely fixed developmental pattern throughout the vegetative stage. MST2 predicts a slightly different relationship in which scaling slopes are unity for young small plants but then quickly adjust to the values predicted by MST1 at some transition point during development. Although plants < 1 g indeed show relatively fixed scaling exponents close to 1.0, plants that achieve a size of 1–10 g begin to adjust their scaling exponents gradually, with an increasing fraction of biomass in stems (Fig. 2). Most data for larger plants are from trees growing in plantations or natural forests. In the case of stands with equally sized individuals, the amount of light, nutrients and water available to an individual are directly affected, and probably restricted, by neighbours. Because these neighbouring trees limit horizontal crown expansion, increasing leaf mass will generally manifest as increased leaf area per unit ground area (LAI). Because very little light remains to be intercepted when LAI exceeds a value of *c*. 3–5 (Ellsworth & Reich, 1993; Anten *et al*., 1995), there will be little photosynthetic return on new leaf area investment, making more leaf area unprofitable for a given individual. Indeed, forests in that growth phase often reach a plateau in leaf biomass or may even decline in leaf mass (Ryan *et al*., 1997; Fernández‐Martínez *et al*., 2014). Although the response of an individual tree may be different from that of a stand, the core assumption linking energy capture to biomass in MST – namely, that plant growth is always proportional to the leaf mass present (Price *et al*., 2010) – is clearly incorrect in closed canopies, where leaf biomass earns diminishing returns. However, competition for light necessitates further investments in stem growth, most importantly in height but also in diameter, for mechanical safety. Mechanical safety also necessitates additional root growth. Therefore, for competing trees whose lateral crown expansion is restricted by neighbours, we expect leaf biomass to saturate as the profitability of investments in leaves declines, with investments gradually shifting to stems and roots, and more so to stems as a consequence of the direct benefit of height growth *per se* for light competition (Dybzinski *et al*., 2011). These expectations were supported by our data set, for which we found a substantial decline in the leaf vs root and leaf vs stem scaling exponents with increasing plant size.

For trees over 10–100 kg, the strong prioritization of stem biomass distribution decreases somewhat, with stem vs root scaling returning to unity again. At the same time, the relative change in leaf biomass is at its lowest point, with scaling exponents decreasing to as low as 0.66. An explanation for these changes could involve the negative effect of height on water transport, which can lead to a limitation of stomatal conductance and thus photosynthetic rate and growth (Koch *et al*., 2004; Ryan *et al*., 2006; Steppe *et al*., 2011). These hydraulic factors, as well as increased requirements for mechanical stability, may also favour greater biomass distribution to roots in tall trees (Nicoll & Ray, 1996). Interestingly, a recent paper specifically modelled the architectural changes as well as the changes in hydraulics during ontogeny and predicted the metabolic scaling exponent to decrease to 0.64 for large trees (Smith *et al*., 2014). Alternative explanations that are inconsistent with quarter‐power scaling involve the influence of nutrient and/or water relations on coupled carbon, nutrient, and water scaling (e.g. Reich *et al*., 2006; Savage *et al*., 2010).

We formalized our ideas of the role that increased biomass distribution to stem biomass may play both in favouring light competition and in hindering water transport in a very simple mathematic model (Notes S3). Plants without constraints on and/or rewards for height growth show a constant, isometric biomass distribution throughout their life in this model. However, when the model is modified to reward height growth, biomass distribution shifts in favour of stems, with the stem vs root and leaf vs stem scaling exponents increasing and decreasing, respectively, as we observed in our data. A more sophisticated model that applied a teleonomic approach to more detailed descriptions of canopy physiology and included mechanical safety constraints (DESPOT; Buckley & Roberts, 2006b) gave similar predictions, with leaf vs stem and leaf vs root scaling declining during growth to 0.64 and 0.62, respectively. We do not suggest that either of these models captures all subtleties of the biology of biomass distribution, but they do demonstrate that allometric scaling exponents are very likely to change with plant size as a result of size‐related changes in the return on investment in various organs. Moreover, as these allometric trends were predicted by developmental models, they apply equally well to comparisons in the ontogenetic and in the evolutionary domains. Together, these models and our data strongly contradict the MST predictions of constant scaling exponents, both empirically and theoretically. We conclude that all results and economic principles are consistent with scaling exponents that change dynamically with plant size.

### Biomass distribution patterns as dependent on size

The virtue of the allometric analyses is that they determine relationships among traits, while implicitly accounting for size differences among plants. However, these analyses generally focus on the scaling exponent ‘*b*’ rather than the allometric constant ‘*a*’ (Glazier, 2010), and provide no insight into the specific values of biomass distribution variables (Poorter & Sack, 2012). Because the leaf vs stem and leaf vs root scaling exponents were < 1.0 over the full biomass range (Fig. 1d,e), it follows that larger plants will have monotonic declines in LMF values, which was indeed the case (Fig. 2a). However, whereas organ size explained > 98% of the variation in the organ allometries, size only explained 78%, 87% and 21% for LMF, SMF and RMF, respectively. The lower *r* ^2^ values in the clasmometric approach can be explained by the fact that the ‘autocorrelative’ effect of larger plants having larger organs is removed in this type of calculation. Part of the remaining variation is probably attributable to differences in environmental conditions, which are difficult to quantify for this data set, especially for availability of nutrients and water, or inherent variation within species. For the analysis of environmental effects the reader is referred to, for example, McCarthy & Enquist (2007), Poorter *et al*. (2012) and Reich *et al*. (2014).

The other part of the remaining variation will be attributable to differences among species. In the following paragraphs, we discuss the extent to which phylogeny and functional group explain overall variation in biomass distribution patterns. As size had such a large influence on biomass distribution patterns (Fig. 2; Coleman *et al*., 1994), we analysed the deviation of each record from the overall trends rather than considering the observed biomass fractions *per se*. Focusing on pLMF as a measure of the deviations from the median, we found clear phylogenetic differences (Figs 3, S2; Table 2), consistent with findings of previous work, but importantly extending the range of variation and the types of comparisons. We discuss next the most interesting and clear contrasts, for woody and herbaceous species separately.

### Interspecific variation in woody species

One of the larger phylogenetic differences we found was that woody gymnosperms invest relatively more in leaves and less in stems than woody angiosperms (Fig. 3; Table 3), in accordance with conclusions of, for example, Körner (1994), McCarthy *et al*. (2007) and Reich *et al*. (2014), which were based on much smaller data sets representing fewer lineages. Interestingly, the few deciduous gymnosperm species deviated markedly in biomass distribution pattern from the evergreen gymnosperms, indicated by their much lower pLMF and higher pSMF. A similar contrast in pLMF between deciduous and evergreen trees was also found in the woody angiosperms, for species characteristic of both tropical/subtropical and temperate/boreal habitats, and in both small‐ and large‐sized individuals. Given that a much larger proportion of gymnosperm than angiosperm woody species are evergreen, it is likely that what was previously concluded to be a phylogenetic difference actually has a functional basis.

Considered over all species for which data on larger trees (> 100 kg) are present, the difference in actual LMF between the two functional groups is more than two‐fold, with mean (± SE) LMF being 0.018 (± 0.0005) for the deciduous species and 0.046 (± 0.0009) for the evergreens. This divergence in LMF could be explained mechanistically by assuming a yearly, fixed allocation of sugars to leaves equal for all tree species, in combination with a much larger leaf turnover in the deciduous species, as a consequence of their two‐ to three‐fold lower leaf lifespan. An alternative and potentially complementary explanation is that plants regulate LMF directly on the basis of the proportion of leaf, stem and root required, with allocation of sugars simply adjusted to that. The latter mechanism is consistent with pruning experiments with herbs, where LMF and RMF quickly recovered to original values after half of the leaf or root mass was removed (Brouwer, 1963; Poorter & Nagel, 2000). What could invoke such setpoints? An explanation at the system level would be based on the fact that forests in most regions of the world function with an LAI that differs little between deciduous and evergreen species (Iio *et al*., 2014). It is also known that, on average, the specific leaf area (SLA; leaf area per unit leaf mass) of evergreen woody species is 2–3 times lower than that of deciduous species (Poorter *et al*., 2009). Hence, all else being equal, the 2–3 times lower SLA in evergreens would have to be compensated by a 2–3 time higher LMF to arrive at the more or less similar LAI. [Correction added after online publication 22 July 2015: in the preceding sentence ‘higher’ has been corrected to read ‘lower’.]

Our large database provides a basis for comparison of individual plant groups in future studies, as it allows discoveries of distinctive biomass distribution in given life forms and clades. As examples, we highlight findings for two groups of evergreen woody species. Our analysis showed that the arborescent palms form a functional group with an especially distinct biomass distribution pattern (Fig. 4). Woody palms are among the dominant species in large part of the tropics (Ter Steege *et al*., 2013) and are particularly well adapted to survive hurricanes, and one might therefore predict a particularly well‐developed root system. However, the little information we were able to collect suggests instead that they have a high LMF, as do the other groups of evergreen species. Another surprise was the consistently high pLMF and low pRMF for Proteaceae, as these species generally come from light‐exposed, dry and nutrient‐poor areas, where large RMFs could be considered of survival value. It is possible that the ephemeral nature of their cluster roots (Lambers *et al*., 2006), that is, fast fine‐root turnover, lead to their low RMF despite a potentially large fraction of photosynthates allocated to roots.

### Interspecific variation in herbaceous species

We found a large contrast in biomass distribution within the herbaceous species between graminoids (Cyperaceae and Poaceae), which showed low pLMFs and high pRMFs, and herbaceous eudicots, which showed the reverse. This difference has also been observed in experiments where graminoids and herbaceous eudicots are grown under the same environmental conditions, and it is consistent with observations that grasslands generally show very high RMF (Jackson *et al*., 1996; Poorter *et al*., 2012). Because graminoids do not show secondary root growth, this may seem counterintuitive. Why do graminoids invest relatively strongly in root mass? One possible explanation is that graminoids must develop more roots from the shoot base to effectively explore the same root volume as eudicots. It has also been reported that roots of graminoids have lower protein concentrations and uptake rates of nitrogen per unit mass (Table 4). Thus, the higher RMF might be a compensation for a lower activity, although cause and effect could be reversed here as well. Other reasons are that graminoids may better survive grazing by quickly developing an extended well‐anchored root system that resists the pulling forces of herbivores (Read & Stokes, 2006); that the storage of starch and nutrients in a larger pool of roots enables more retranslocation to new leaves after grazing or fire; or that grasses, by having less frequent associations with mycorrhizas (Van der Heyden *et al*., 2015), invest more in roots themselves.

**Table 4:** Differences in root characteristics for herbaceous monocots and eudicots, as measured in the same experiment

Variable	Monocots	Eudicots	Difference (%)	P
RMF (g_ROOT_ g^−1^_PLANT_)	0.31 ± 0.01	0.26 ± 0.01	+20	*
[Root organic N] (mg g^−1^_ROOT_)	30 ± 1.5	42 ± 1.3	−29	***
Net NO_3_ uptake rate (mmol g^−1^_ROOT_ d^−1^)	2.4 ± 0.3	3.9 ± 0.4	−40	**
Root respiration (nmol O_2_ g^−1^_ROOT_ s^−1^)	54 ± 2.6	64 ± 3.9	−16	*

This table shows a summary of the overall difference between 11 herbaceous monocot and 13 herbaceous eudicot species. All species were grown in a growth chamber under conditions of unrestricted water and nutrient supply. More details can be found in Poorter *et al*. (1991). The differences were tested at the species level with a Welch two‐sample *t*‐test. Data are mean values ± SE. Significance values: *, *P *<* *0.05; **, *P *<* *0.01; ***, *P *<* *0.001. RMF, root mass fraction.

A second contrast we analysed is between herbaceous C_3_ and C_4_ species. Insofar as C_4_ species are thought to have higher photosynthetic capacities than C_3_ species, high expectations are placed on introducing this mechanism in C_3_ species with the aim of boosting productivity (Von Caemmerer *et al*., 2012). However, given that plants with a superior photosynthetic rate and high sugar availability may readjust their biomass distribution pattern by investing less in leaves and more in roots, the anticipated gains might partly disappear. In contrast, if C_4_ species generally show reduced water loss compared with C_3_ species, they could operate with a higher LMF and a lower RMF. However, comparing the overall biomass distribution between C_3_ and C_4_ herbs, we found evidence of neither scenario in the monocots, as there was no overall difference between C_3_ and C_4_ species. There is some indication for increased pLMF and decreased pRMF in the eudicots, but note that the number of C4 species here is too small for a firm conclusion. We note, furthermore, that C_4_ species have diverse backgrounds, and to better understand the evolutionary details, differences between C_3_ and C_4_ species need to be resolved within given lineages (Taylor *et al*., 2010).

A third noteworthy finding is for a specific family, the Solanaceae, which has exceptionally high pLMF and exceptionally low pRMF values. Many of these species as represented in the database have been bred for cultivation. Whether this has led to changed biomass distribution patterns, however, is still an open question (Milla & Morente‐López, 2015).

Further analysis of the allometric relationships among the major organs showed that the relationship between stems and roots is generally very conserved in woody species. It is likely that herbs have more freedom to change the distribution, as the mechanics of support are less critical than for large trees.

### Conclusions

Using the largest data set with which theories for plant biomass distribution have been tested, we found that plants strongly coordinate the relative sizes of leaves, stems and roots. However, our analyses rejected the ontogenetically fixed scaling exponents predicted by MST, and instead found dynamically shifting scaling exponents and biomass fractions with plant size. Furthermore, we found systematic differences in biomass distribution among species groups, with gymnosperms showing higher biomass present in leaves than angiosperm trees, and graminoids having higher biomass fractions in roots than nongraminoids. In both contrasts, these differences are likely to have a functional basis.

## Supporting information

Please note: Wiley Blackwell are not responsible for the content or functionality of any supporting information supplied by the authors. Any queries (other than missing material) should be directed to the *New Phytologist* Central Office.

**Fig. S1** Residuals of the allometric relationships after fitting an SMA regression through log–log‐transformed organ mass data.
**Fig. S2** Phylogenetic tree of the Leaf Mass Fraction (LMF) data.
**Fig. S3** Distribution of the deviations from the overall allometric log–log curves for various functional groups.
**Table S1** Literature references on which the database is built
**Table S2** Biomass data for leaves, stems and roots as well as biomass distribution patterns and deviations from the overall trends in biomass distribution as used in the current analyses
**Table S3** Overview of the representation of various plant groups in the database
**Table S4** Allometric scaling exponents as given for all records of all species, and for herbaceous and woody species separately
**Notes S1** Methodological assumptions.
**Notes S2** The inference value of *r* ^2^ in plant allometric analyses.
**Notes S3** Explanation of a very simple model that shows dynamic scaling exponents as a consequence of physiological or environmental constraints.
