# Supplementary material for: How does biomass distribution change with size and differ among species? An analysis for 1200 plant species from five continents
Source: New Phytol. 2015 Jul 22;208(3):736–49. doi: 10.1111/nph.13571 (PMC5034769; doi:10.1111/nph.13571)
Supplement: Supplementary file 1 — Fig. S1 Residuals of the allometric relationships after fitting an SMA regression through log–log‐transformed organ mass data. Fig. S2 Phylogenetic tree of the Leaf Mass Fraction (LMF) data. Fig. S3 Distribution of the deviations from the overall allometric log–log curves for various functional groups. Table S1 Literature references on which the database is built Table S2 Biomass data for leaves, stems and roots as well as biomass distribution patterns and deviations from the overall trends in biomass distribution as used in the current analyses Table S3 Overview of the representation of various plant groups in the database Table S4 Allometric scaling exponents as given for all records of all species, and for herbaceous and woody species separately Notes S1 Methodological assumptions. Notes S2 The inference value of r 2 in plant allometric analyses. Notes S3 Explanation of a very simple model that shows dynamic scaling exponents as a consequence of physiological or environmental constraints. [file NPH-208-736-s001.pdf]

New Phytologist **Supporting Information**

Article title: **How does biomass allocation change with size and differ among species? An analysis for 1200 plant species from five continents**
Authors: Hendrik Poorter, Andrzej M. Jagodzinski, Ricardo Ruiz-Peinado, Shem Kuyah, Yunjian Luo, Jacek Oleksyn, Vladimir A. Usoltsev, Thomas N. Buckley, Peter B. Reich, Lawren Sack
Article acceptance date: 15 June 2015

The following Supporting Information is available for this article:

Fig. S1 Residuals of the allometric relationships after fitting a SMA regression through log-log transformed organ mass data.

Fig. S2 Phylogenetic tree of the Leaf Mass Fraction (LMF) data.

Fig. S3 Distribution of the deviations from the overall allometric log-log curves for various functional groups.

- **Note S1** Methodological assumptions

Note S2 Inference value of r^2 in plant allometric analyses

Note S3 Explanation of a very simple model that shows dynamic scaling exponents as a consequence of physiological or environmental constraints.

Table S1 Literature references on which the database is built.

Table S2 Biomass data for leaves, stems and roots as well as biomass distribution patterns (LMF, SMF and RMF) and deviations from the overall trends in biomass distribution (pLMF, pSMF and pRMF) as used fr the current analyses.

Table S3 Overview of the representation of various plant groups in the database.

Table S4 Allometric scaling exponents as given for all records of all species, and for herbaceous and woody species separately.

Fig. S1 Residuals after fitting a SMA regression through log-log transformed organ mass data. For clarity, the residuals are binned for the 50 size classes, and the median residuals for each size class are connected with a red line.

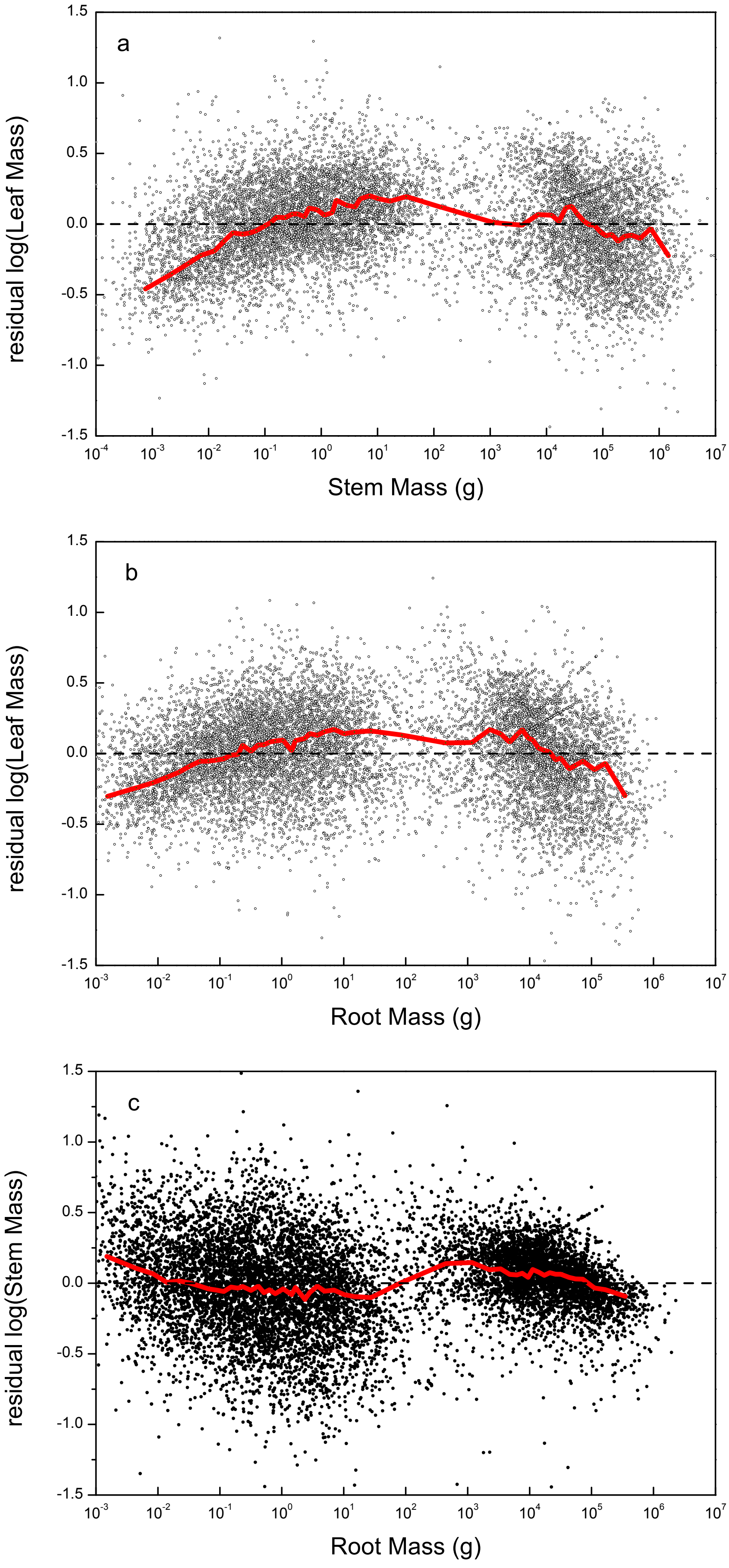
a
b
c
residual log(Leaf Mass)
residual log(Stem Mass)
Stem Mass (g)
Root Mass (g)
1.5
1.0
0.5
0.0
-0.5
-1.0
-1.5
10^{-4}
10^{-3}
10^{-2}
10^{-1}
10^{0}
10^{1}
10^{2}
10^{3}
10^{4}
10^{5}
10^{6}
10^{7}

Fig. S2 Phylogenetic tree of the Leaf Mass Fraction (LMF) data. Data are based on the deviations of the LMF of each record from the median trend line (pLMF) as shown in Fig. 2*a*, expressed as percentiles and calculated per size class (see Methods). Note that a pLMF of 50 indicates that the species does not deviate from the median trend. The total number of species included is 482. The species colour indicates the average pLMF for that species, for explanation of the colour code see the legend of the figure.

Poorter et al.

Suppl. Fig. 2

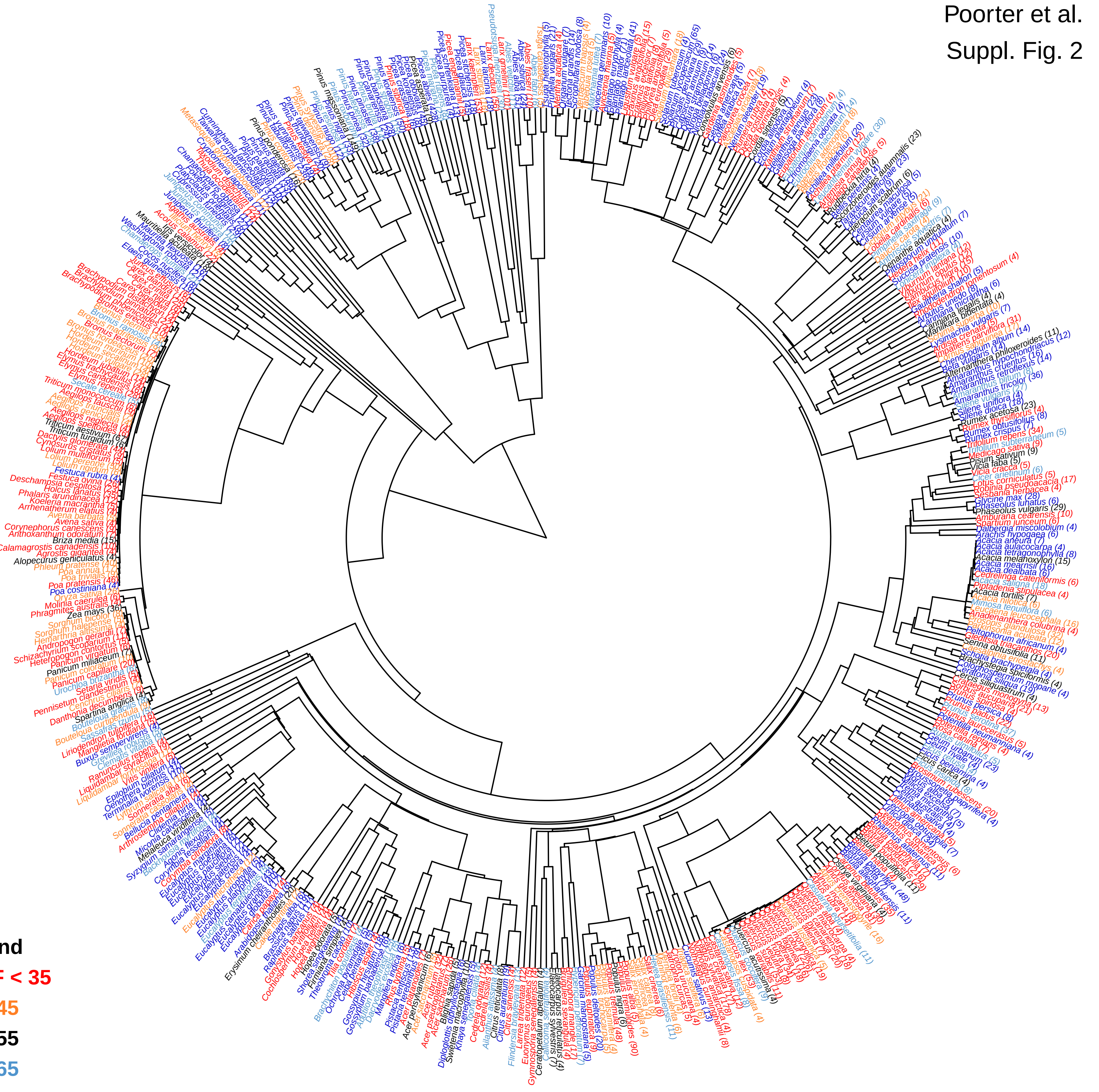

Legend

pLMF < 35

35 – 45

45 – 55

55 – 65

> 65

Fig. S3 Distribution of the deviations from the overall allometric log-log curves for various functional groups for (a) Leaf mass vs. stem mass scaling (b) Leaf mass vs. Root mass scaling and (c) Stem mass vs. Root mass scaling. The overall allometric curves were first determined by a Loess fit, and for all observations the deviations from the fitted line were calculated and averaged over species. Species averages were then used to determine boxplots of the distribution of values per species. The boxplot indicates the 10th, 25th, 50th, 75th and 90th percentile. Wo.: woody; He.: herbaceous; Evg.: evergreen; Dec.: deciduous; Perenn.: perennial; Gymn.: gymnosperms; Angio.: angiosperms; Mono: monocotyledons; Eudico.: Eudicotyledons. Significance values indicate t-tests for significant deviation from 0, which indicates no deviation from the overall allometric curve. +, $0.05 < P < 0.10$; *, $P < 0.05$; **, $P < 0.01$; ***, $P < 0.001$).

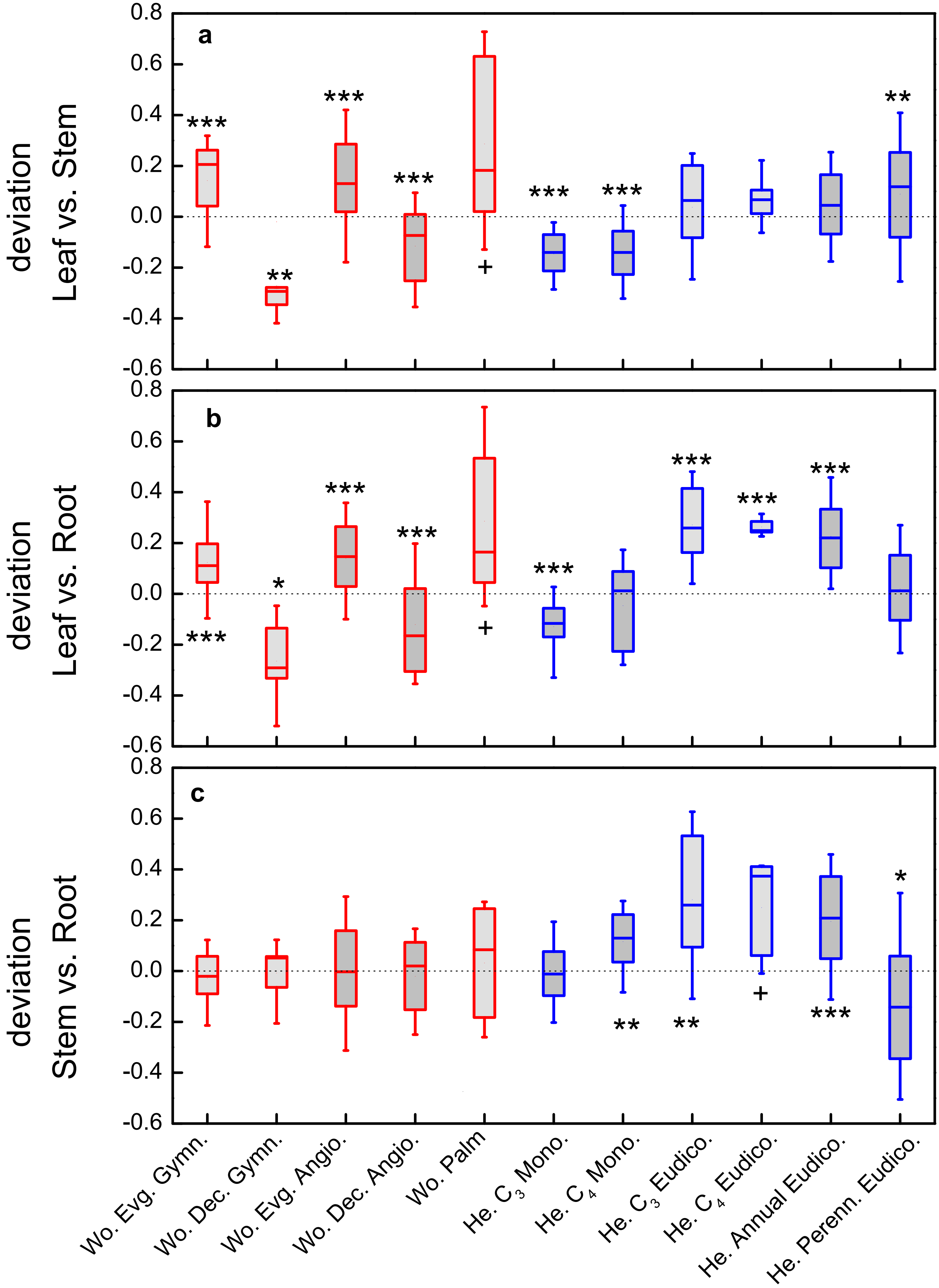
a
b
c
deviation
Leaf vs. Stem
deviation
Leaf vs. Root
deviation
Stem vs. Root
0.8
0.6
0.4
0.2
0.0
-0.2
-0.4
-0.6
***
**
+
*
Wo. Evg. Gymn.
Wo. Dec. Gymn.
Wo. Evg. Angio.
Wo. Dec. Angio.
Wo. Palm
He. C3 Mono.
He. C4 Mono.
He. C3 Eudico.
He. C4 Eudico.
He. Annual Eudico.
He. Perenn. Eudico.

Note S1. Methodological assumptions

There were four important assumptions in our methodology, shared with previous studies. First, we assumed that differences among studies in how the whole plant was separated into leaf, stem and root would have only a small effect on the patterns analysed. Not all investigators followed exactly the same protocol, and not all described their specific protocol. Ideally, organs would be consistently defined by function; for example, leaf lamina, as the organ most active in photosynthesis, would ideally be consistently separated from petioles and, in grasses, from leaf sheaths, which mainly function to position the leaf blades in a favourable location and for water and sugar transport (Pérez-Harguindeguy et al., 2013 and references therein). However, in a number of reports petioles and/or leaf bases were included in the leaf fraction. We made a correction only in the case of palm trees, where leaves can be up to 8 meters long with petioles as thick as small branches of dicotyledonous trees (Corley & Tinker, 2003). In those cases we subtracted petiole and rachis from total leaf mass, using supplementary data of other publications as necessary. We also presumed that differences among studies in the separation of stems and roots would not have a strong effect on the analyses.

Our second assumption was that root mass was accurately measured. The extraction of roots from solid substrates can be challenging (Mokany et al., 2006). On the one hand, root mass can be overestimated if roots are not cleaned well enough of adhering soil particles; on the other hand, root mass is underestimated if not all roots are recovered. Based on sophisticated methods where the root diameter of various roots were taken into account, the fraction of unrecovered root mass or volume for a Pinus root system was estimated to be 17% in young plants but only 4% in older ones (Danjon et al., 2013; Chmura et al., 2013). The effect of such error would be relatively modest, though for large field-grown trees no estimates are available.

Third, we assumed that total plant biomass is a far more important driver for biomass distribution than the age or – for trees – the ontogenetic stage of a plant. Some studies focused on size-related changes within species, while others, especially in zoology, have focused on differences across species when measured at the adult reproductive stage. In plants, modular and indeterminate growth generates far more variability in size at any ontogenetic stage than in animals, and size imposes a variety of functional constraints. It is also a strong determinant in competition. We therefore decided to include both younger and older plants, as long as they matched the criteria given in Material and Methods. Ontogeny will be an important factor, especially when flowering occurs in monocarpic species. We therefore excluded all herbs as well as monocarpic woody species in the generative phase. Consequently, the overall database has both ontogenetic and interspecific components, and total plant size is considered to be the main driver.

Our fourth assumption was that the plants for each size class in the dataset constitute a representative subsample with respect to growth conditions, functional group, and phylogeny. That is, for all sizes of plants there are individuals grown at various environmental conditions (high/low temperature, nutrients, densities, etc.). Larger trees were only grown and harvested in the field, so we also assume that the mix of conditions for small trees (partly experimental, partly field) was not very different from those of larger trees (partly plantations, partly natural). Thus, we supposed environmental effects to be randomly and equally distributed over the full range of data, adding noise to the data set, but without driving any substantial systematic deviations.

References

- **Chmura DJ, Guzicka M, Rozkowski R, Chałupka W. 2013.** Variation in aboveground and belowground biomass in progeny of selected stands of *Pinus sylvestris*. *Scandinavian Journal of Forest Research* **28**: 724-734.
- **Corley RHV, Tinker PB. 2003.** *The Oil Palm.* Oxford, UK: Blackwell Science.
- **Danjon F, Caplan JS, Fortin M, Meredieu C. 2013.** Descendant root volume varies as a function of root type: estimation of root biomass lost during uprooting in *Pinus pinaster*. *Frontiers in Plant Science* **4**: 402.
- **Mokany K, Raison RJ, Prokushkin AS. 2006.** Critical analysis of root:shoot ratios in terrestrial biomes. *Global Change Biology* **12**: 84-96.
- **Pérez-Harguindeguy N, Díaz S, Garnier E, Lavorel S, Poorter H, Jaureguiberry P, Bret-Harte MS, Cornwell WK, Craine JM,Gurvich DE *et al.* 2013.** New handbook for standardised measurement of plant functional traits worldwide. *Australian Journal of Botany* **61**: 167-234.

Note S2. Inference value of r^2 in plant allometric analyses

Sensitivity analysis of the effect of a predefined range in Root Mass Fraction (RMF: fraction of Total Plant Mass (TM) invested in roots) on the explained variance (r^2) of the relationship between log(root mass) and log(shoot mass). The predefined ranges were chosen to be narrow from a biological perspective (0.4 < RMF < 0.6), realistic (0.07 < RMF < 0.70; as observed for the current data set based on the 0.5th and 99.5th percentile), very wide (0.01 < RMF < 0.99) and improbably wide (0.001 < RMF < 0.999). Each correlation was based on 20,000 random drawings as explained below. Overall variation in simulated plant mass was either 10 orders of magnitude, or 1 order of magnitude.

	**r^2**	
**Range in RMF**	**10^{10}-fold range in TM**	**10-fold range in TM**
0.40 – 0.60	0.999	0.886
0.07 – 0.70	0.982	0.170
0.01 – 0.99	0.941	0.011
0.001 – 0.999	0.932	0.020

Simulations

To evaluate the meaning to be attached to the actual value of r^2 in allometric relationships, we simulated a population of plants with substantial variation in biomass, drawing randomly from a uniform distribution of log-transformed total plant mass, for a range that comprised 10 orders of magnitude in biomass. This is more or less similar to the current dataset. For each of these plants, root mass was then calculated, after simulating a Root Mass Fraction by randomly drawing from a uniform distribution with values ranging from – in the first scenario - 0.40 to 0.60. Shoot mass was then simply the difference between the mass of the total plant and the roots. We drew samples for 20,000 simulated plants in total, and subsequently calculated the r^2 of the allometric relationship between the log-transformed shoot and root mass for all 20,000 observations. The analysis was then repeated with three other scenarios with increasingly wider intervals for the RMF.

The compiled data showed very tight relationships between organs (r^2 ≥0.932; see table above), and between each organ and total plant biomass (r^2 ≥0.992). We analysed how such high r^2 values for the relationships between the biomass of two organs should be interpreted, and whether it permits the inference of a fixed scaling exponent, in principle. We simulated cases with various ranges in RMF. In the case of relatively narrow variation in RMF (0.40-0.60), we found that the allometric relationship yielded an r^2 higher than 0.99. However, when allowing

RMF to vary randomly across the range actually observed in our compiled dataset (99% of the cases were in the 0.07-0.70 range), the r^2 still exceeded 0.98. Only in simulations when the range in allocation became much larger than normally observed (0.01 – 0.99 or wider) was the r^2 <0.95. Indeed, even if the shoot and root system of plants showed highly variable proportionality, that is when RMF ranged between 0.01 and 0.99 (and thus, shoot:root ratios were confined between 0.01 and 99), the correlation between log-transformed shoot and root biomass was still around 0.94 or higher. The high r^2s were predominantly due to the large range of biomass considered; the r^2 dropped dramatically with increasing range of RMF when the same allometric relationship is considered over one rather than ten orders of magnitude in plant size (Table above). Hence, the fact that an allometric equation “explains” ~97% of the variation in shoot and root mass according to its r^2, does not allow the inference of the central tendency of biomass allocation. Direct examination of the allometries, and potential shifts in the allometries are necessary to establish the central trend, and whether it is fixed or dynamic.

Note S3. Accounting for changes in the economics of C allocation during growth

The central features of the economics of plant carbon allocation are that the effect of C investment in any pool on growth must be saturating – i.e., growth rate *G* approaches a finite limit as C in any pool approaches infinity – and that the effects of each C investment on growth rate are not independent: if investment is high in one pool, this increases the return on investment in other pools due to their mutual influence on photosynthesis. Perhaps the simplest implementation of these features is

$$G \propto \left(\frac{c_s}{c_s + k_s}\right)\left(\frac{c_r}{c_r + k_r}\right)\left(\frac{c_l}{c_l + k_l}\right)$$

Apart from the saturation constants kj, the carbon pools are indistinguishable in this model. When carbon allocation is optimised in this model (by modulating allocation over time, during growth, so that the marginal products for each pool remain equal: $\partial G/\partial c_s = \partial G/\partial c_r = \partial G/\partial c_l$), allocation is isometric:

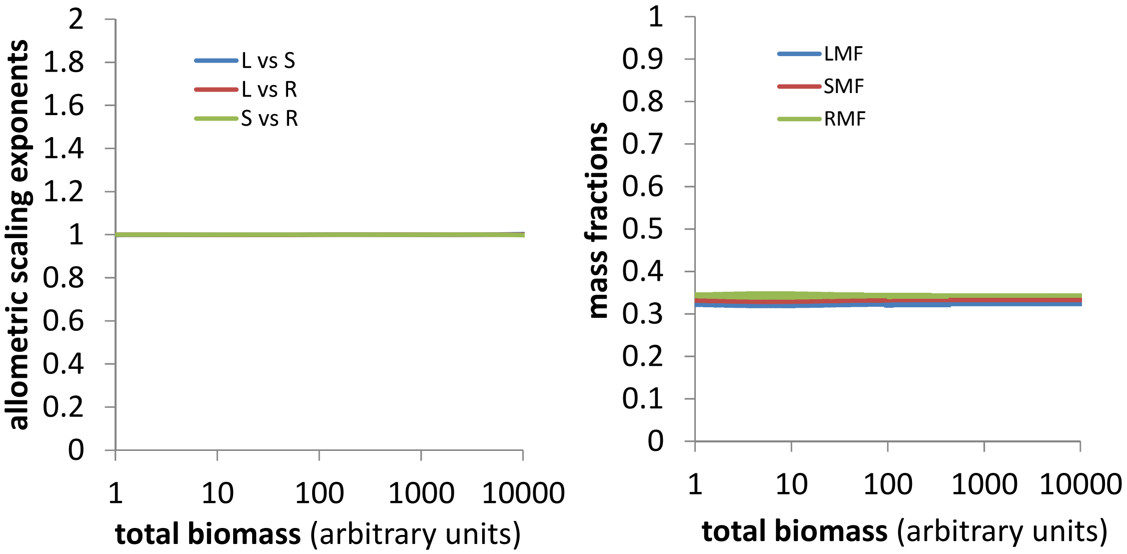

(The lines for LMF and RMF have been shifted up by 0.01 and down by 0.01, respectively, to make them visible.) To capture what happens during height growth, we must incorporate the effect of height, which influences the economics of allocation to stems, roots and leaves differently. Suppose that height growth brings diminishing returns because its benefits for light capture are counteracted by its negative impact on water transport. This can be expressed qualitatively by multiplying G by a hyperbolic function of height analogous to the terms for the carbon pools:

$$G \propto \left(\frac{c_s}{c_s + k_s}\right)\left(\frac{c_r}{c_r + k_r}\right)\left(\frac{c_l}{c_l + k_l}\right)\left(\frac{h}{h + k_h}\right)$$

where h is height. Height also depends on stem carbon. Supposing that height is proportional to the 2/3 power of stem diameter as specified by buckling theory, and that stem carbon is proportional to the product of height and diameter, it follows that $h \propto c_s^{1/(1+1/(2/3))} = c_s^{2/5} = c_s^{\bullet/(\alpha+1)} = c_s^{\beta}$ where $\beta = \alpha/(\alpha+1)$ and $\alpha = 2/3$ and $\beta = 2/5$ nominally. Then

$$\frac{h}{h + k_h} = \frac{c_s^{\beta}}{c_s^{\beta} + k_h}$$

and

$$G \propto \left(\frac{c_s^{\beta+1}}{(c_s + k_s)(c_s^{\beta} + k_h)}\right)\left(\frac{c_r}{c_r + k_r}\right)\left(\frac{c_l}{c_l + k_l}\right)$$

This model predicts increasing scaling exponents for stem relative to roots, decreasing exponents for leaf relative to stem, increasing SMF and decreasing RMF and SMF -- as observed:

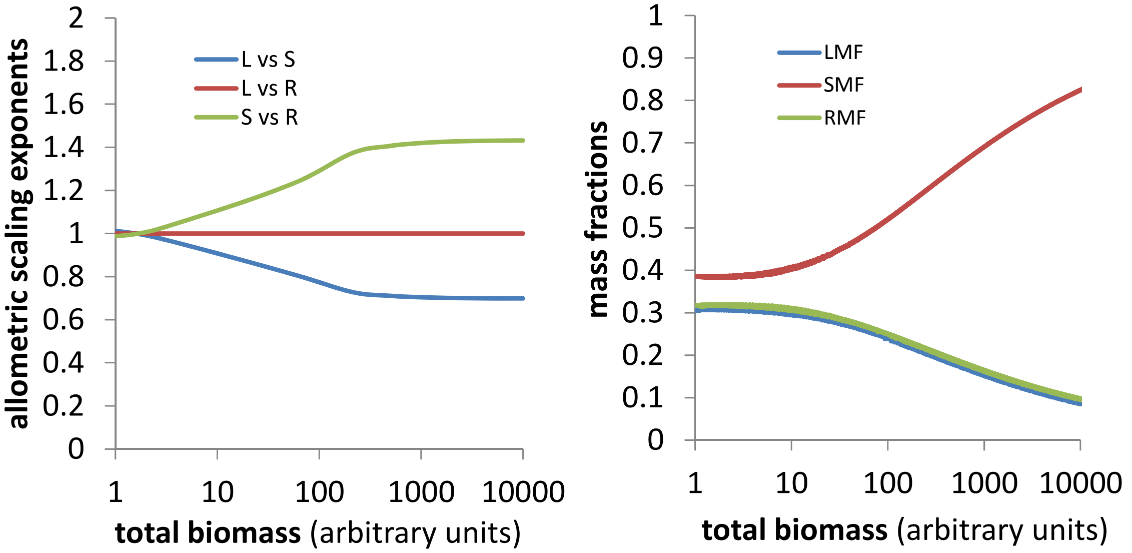

Interpretation

We do not suggest that this model should be directly interpreted as a mechanistic model for predicting changes in allometry during plant growth and neither should be the exact values of scaling exponents or mass fractions. For one, it omits many important features that may influence the economics of carbon allocation, such as the mechanical demands for below-ground anchorage, the effect of leaf investment on hydraulic conductance, the respiratory and hydraulic costs of height growth, the loss of leaf and fine root investments to senescence and the conversion of sapwood to heartwood, and the return of nutrients to the soil from leaf litter

during growth. To incorporate all of these features requires a radically more detailed and mechanistically explicit model, and it is unlikely that any such model will apply generally enough across types of plants and environments to permit general theories of allometric scaling to be deduced. Instead, the analysis above shows that when MST's fundamental assumption about plant carbon economics – namely, that growth is directly proportional to leaf biomass – is minimally elaborated to include qualitative features of the economics of stem and root carbon investment, it quickly emerges that allometric scaling constants should vary continuously and in complex fashion during plant growth.

Simulation methods

We simulated the growth model described above using $k_s = k_r = k_l = 1$ with initial biomass pool sizes equal to 1/6 so that the total initial biomass was 0.5. For simulations including the saturating positive effect of height, we set $k_h = 1$. In each time step, the marginal product for each biomass pool was computed numerically using a small biomass increment (10^{-5}), and the biomass pool with the greatest marginal product was incremented by the biomass available for allocation, which was computed as the product of *G* and an arbitrary timestep of 0.3. This ensured that the marginal products remained approximately equal across biomass pools during the simulated growth period.

Table S1. Literature references on which the database is built.

Herbaceous species:

A-E

Alameda et al. (2012) Soil Till. Res. 120: 121-129; Ali (2000) Plant Sci. 152: 173-179; Ali et al. (2013) J. Plant Growth Regul. 32: 604-614; Almeida et al. (1999) Plant Soil 210: 159-166; Ambrose & Sciuchetti (1962) J. Pharm. Sci. 51: 934-938; Andrews et al. (1992) Ann. Bot. 70: 271-276; Anten et al. (2003) Ecology 84: 2905-2918; Balachandran et al. (1997) Plant Physiol. 114: 475-481; Bar & Pressman (1996) J. Am. Soc. Hortic. Sci. 121: 649-655; Barker et al. (1996) New Phytol. 133: 637-642; Barnes et al. (1993) Crop Sci. 33: 1041-1046; Barrett & Gifford (1995) J. Biogeogr. 22: 331-339; Bar-Tal et al. (1995) Sci. Hortic. 63: 195-208; Baruch et al. (2000) Int. J. Plant Sci. 161: 107-118; Benner & Bazzaz (1988) J. Ecol. 76: 19-40; Bennett & Runeckles (1977) Crop Sci. 17: 443-445; Boot & Den Dubbelden (1990) Oecologia 85: 115-121; Bourdot et al. (1984) New Phytol. 97: 653-663; Brown et al. (1995) J. Am. Soc. Hortic. Sci. 120: 808-813; Brummett & Sciuchetti (1960) J. Pharm. Sci. 49: 274–277; Bullock (1984) Oecologia 63: 426-428; Bunce (1990) Ann. Bot. 65: 637-642; Carmi et al. (1983) Photosynthetica 17: 240-245; Carter et al. (1997) New Phytol. 136: 245-253; Causin & Wulff (2003) Can. J. Bot. 81: 152-163; Chabot (1978) New Phytol. 80: 87-98; Chapin et al. (1989) Oecologia 79: 96-105; Clevering (1997) Wetlands Ecol. Manage. 5: 275-287; Clough et al. (1979a) Oecologia 38: 13-21; Clough et al. (1979b) Plant Physiol. 64: 25-30; Coleman & Bazzaz (1992) Ecology 73: 1244-1259; Corre (1983b) Acta Bot. Neerl. 32: 185-202; Cowan & Reekie (2008) Plant Biol. 10: 202-210; Cramer et al. (1995) J. Exp. Bot. 46: 1569-1577; Cris & Stout (1929) Agric. Exp. Stat. MSC, No 6.; Cunniff et al. (2008) Glob. Change Biol. 14: 576-586; Cure et al. (1987) Bot. Gaz. 148: 67-72; Dai et al. (1992) Crop Sci. 32: 1269-1274; Dale (1964) Ann. Bot. 28: 127-135; De Groot et al. (2001) Plant Cell Environ. 24: 1309-1317; De Pinheiro & Marcelis (2000) Ann. Bot. 86: 1073-1080; Debez et al. (2004,2006) Plant Soil 262: 179-189; Env. Exp. Bot. 57: 285-295; Delgado et al. (1993) J. Exp. Bot. 44: 1-7; DeLucia et al. (1985) Photosynth. Res. 7: 175-184; Den Dubbelden & Oosterbeek (1995) Funct. Ecol. 9: 628-634; Den Hertog et al. (1993) Vegetatio 104/105: 369-387; Den Hertog et al. (1996) Physiol. Plant. 98: 77-88; Detling et al. (1979) Oecologia 41: 127-134; Dias-Filho (2002) Pesq. agropec. Bras. 37: 439-447; Dippery et al. (1995) Oecologia 101: 13-20; Donnelly et al. (2001) New Phytol. 149: 265-271; Dueck et al. (1999) AB-DLO report 207, WUR; Dunn et al. (1987) J. Exp. Bot. 38: 433-441; Elemans (2004) Acta Oecol. 26: 197-202; Eng et al. (1985) J. Hortic. Sci. 60: 389-395; Evans & Hughes (1961) New Phytol. 60: 150-180; Evans & Poorter (2001) Plant Cell Environ. 24: 755-767; Eze et al. (1973) Ann. Bot. 37: 315-329;

F-J

Feng et al. (2007) Acta Oecol. 31: 40-47; Ferris & Taylor (1993) New Phytol. 125: 855-866; Flint et al. (1984) Weed Sci. 32: 655-666; Flynn et al. (2006) Env. Exp. Bot. 56: 10-18; Franzaring et al. (2000) Env. Exp. Bot. 44: 39-48; Funnell et al. (1998) J. Am. Soc. Hortic. Sci. 123: 973-979; Garnier (1992) J. Ecol. 80: 665-675; Garnier et al. (1989) Oecologia 79: 542-550; Ghannoum & Conroy (1998) Aust. J. Plant Physiol. 25: 627-636; Gibson et al. (2001) Weed Res. 41: 59-67; Gifford (1991) NERDDC report 1402; Gimplinger & Kaul (2009) J. Appl. Bot. Food Qual. 82:

183-192; Gloser & Bartak (1994) Photosynthetica 30: 143-150; Groeneveld HW. (unpubl.); Gunn et al. (1999) Funct. Ecol. 13: 3-11; Guzman & Olave (2006) J. Food Agric. Environ. 4: 163-165; Hameed et al. (1987) Ann. Bot. 59: 685-692; Hao et al. (1997) Can. J. Bot. 75: 213-219; Hardacre & Turnbull (1986) Ann. Bot. 58: 779-787; Harmens et al. (2000) Ann. Bot. 86: 833-839; Heide et al. (1985) Physiol. Plant. 65: 135-145; Hibberd et al. (1996) New Phytol. 134: 309-315; Hirose & Kitajima (1986) Ann. Bot. 58: 479-486; Hocking & Meyer (1985) Ann. Bot. 55: 835-844; Hocking & Meyer (1991) Aust. J. Plant Physiol. 18: 339-356; Hoddinott & Hall (1982) Can. J. Bot. 60: 1285-1291; Hofstra et al. (1985) Physiol. Plant. 63: 13-18; Holt (1988) J. Appl. Ecol. 25: 307-318; Horsman et al. (1980) Aust. J. Plant Physiol. 7: 511-517; Horsman et al. (1981) Aust. J. Plant Physiol. 8: 405-408; Hossain et al. (2012) Aust. J. Crop Sci. 6: 480-487; Hughes & Evans (1963) New Phytol. 62: 367-388; Hurd (1968) Ann. Bot. 32: 531-542; Hurd (1974) Ann. Bot. 38: 613-623; Hurry et al. (1995) Plant Physiol. 109: 697-706; Imai & Murata (1976) Proc. Crop Sci. Soc. Jpn. 45: 598-606; Ishikawa & Kachi (2000) Ecol. Res. 15: 241-247; Jablonski (1997) Can. J. Bot. 75: 533-545; James (2008) Plant Soil 310: 201-210; Jeangros & Nosberger (1992) Weed Res. 32: 311-316;

K-O

Kalapos et al. (1996) Plant Soil 185: 137-149; Kanno et al. (2009) Soil Sci. Plant Nutr. 55: 124-131; Khan & Tsunoda (1970a) Tohuko J. Agric. Res. 21: 47-59; Khan & Tsunoda (1970a) Tohuko J. Agric. Res. 21: 60-72; Khavari-Nejad & Chaparzadeh (1998) Photosynthetica 35: 461-466; Khavari-Nejad (1988) Photosynthetica 22: 116-120; Kirkby & Mengel (1976) Plant Physiol. 42: 6-14; Knight & Mitchell (1988) J. Exp. Bot. 39: 317-328; Konings et al. (1989) Oecologia 80: 111-121; Kraus et al. (1993) New Phytol. 123: 39-44; Lavinsky et al. (2007) New For. 34: 41-50; Lenssen et al. (1993) Vegetatio 104/105: 379-388; Lenssen et al. (1995) Aq. Bot. 50: 181-192; Lewis et al. (1999) Planta 210: 104-114; Liu & Stutzel (2004) Sci. Hortic. 102: 15-27; Liu & Stützel (2004) Sci. Hortic. 102: 15-27; Lotscher & Nosberger (1997) Oecologia 111: 499-504; Loveys et al. (2002) Plant Cell Environ. 25: 975-988; Lucas et al. (1993) Planta 190: 88-96; Luo et al. (2009) Ann. Bot. 104: 1435-1444; MacDowall (1972) Can. J. Bot. 50: 89-99; Malik et al. (2001) Aust. J. Plant Physiol. 28: 1121-1131; Maranon & Grubb (1993) Funct. Ecol. 7: 591-599; Maroco et al. (1999) Planta 210: 115-125; McKenna & Shipley (1999) EcoScience 6: 286-296; Meijkamp et al. (2001) Plant Ecol. 154: 137-146; Meziane & Shipley (1999) Funct. Ecol. 13: 611-622; Mielke et al. (2003) Env. Exp. Bot. 50: 221-231; Miller et al. (1998) Crop Sci. 38: 122-128; Mingo et al. (2004) Funct. Plant Biol. 31: 971-978; Miranda et al. (2010) Agron. Colomb. 28: 165-172; Mommer et al. (2006) J. Ecol. 94: 1117-1129; Mommer et al. (2007) New Phytol. 176: 337-345; Mooney et al. (1995) Oecologia 104: 17-23; Morison & Gifford (1984) Plant Physiol. 75: 275-277; Muller & Garnier (1990) Oecologia 84: 513-518; Nabel M. et al. (unpubl.); Nagel et al. (2001) Physiol. Plant. 111: 33-39; Nagel K. (unpubl.); Nishizawa & Saito (1998) J. Am. Soc. Hortic. Sci. 123: 581-585; Nogues et al. (1998) Plant Physiol. 117: 173-181; Oberbauer et al. (1986) Can. J. Bot. 64: 2993-2998; Olff (1992) Oecologia 89: 412-421; Olszyk & Wise (1997) Agric. Ecosyst. Environ. 66: 1-10; Olufolaji & Tayo (1980) Sci. Hortic. 13: 181-189; Omami et al. (2006) N. Z. J. Crop Hortic. Sci. 34: 11-22; Overdieck et al. (1988) Angew. Botan. 62: 119-134;

P-T

Paez et al. (1983) Physiol. Plant. 58: 161-165; Patterson & Flint (1980) Weed Sci. 28: 71-75; Patterson & Flint (1982) Weed Sci. 30: 389-394; Patterson & Flint (1983) Weed Sci. 31: 318-

323; Patterson (1980) Weed Sci. 28: 735-740; Patterson (1986) Weed Sci. 34: 203-210; Patterson et al. (1988) Weed Sci. 36: 751-757; Paulilo et al. (2007) Aust. J. Bot. 55: 795-802; Pavlik (1983) Oecologia 57: 227-232; Peace & Grubb (1982) New Phytol. 90: 127-150; Peet (1986) Plant Physiol. 80: 59-62; Penuelas et al. (1993) Photosynthetica 29: 535-542; Peterson et al. (1991) J. Exp. Bot. 42: 1233-1240; Pilon-Smits et al. (1995) Plant Physiol. 107: 125-130; Pons & Poorter (2014) Front. Plant Sci. 5: 12; Pons (1977) Acta Bot. Neerl. 26: 29-42; Pons T.L. & Verkaar D. (unpubl.); Poorter & Nagel (2000) Aust. J. Plant Physiol. 27: 595-607; Poorter & Pothmann (1992) New Phytol. 120: 159-166; Poorter & Remkes (1990) Oecologia 83: 553-559; Poorter & Van der Werf (1998) In: Lambers et al.:pp. 309-336; Poorter (1991) Thesis, Utrecht; Poorter (1993) Vegetatio 104/105: 77-97; Poorter (1999) Funct. Ecol. 13: 396-410; Poorter et al. (1988) Physiol. Plant. 73: 553-559; Poorter et al. (1995) Plant Soil 171: 217-227; Poorter et al. (2005) Oecologia 142: 360-377; Qaderi et al. (2006) Physiol. Plant. 128: 710-721; Raynal et al. (1985) Ann. Bot. 55: 893-897; Read & Morgan (1996); Read et al. (1997) Ann. Bot. 77: 487-496; Ann. Bot. 79: 197-206; Reich et al. (2003) New Phytol. 157: 617-631; Retuerto & Woodward (1993) Oecologia 94: 415-427; Rice & Bazzaz (1989) Oecologia 78: 502-507; Rodriguez et al. (2005) Env. Exp. Bot. 53: 113-123; Roumet et al. (1996) New Phytol. 133: 595-603; Roumet et al. (2000) Env. Exp. Bot. 43: 155-169; Rudmann et al. (2001) Ann. Bot. 88: 571-577; Ryle et al. (1992) J. Exp. Bot. 43: 811-818; Ryser & Lambers (1995) Plant Soil 170: 251-265; Ryser et al. (1997) New Phytol. 137: 293-302; Sallaku et al. (2009) J. Food Agric. Environ. 7: 869-872; Sasek & Strain (1988) Weed Sci. 36: 28-36; Schippers & Olff (2000) Plant Ecol. 149: 219-231; Schulte et al. (2001) J. Agron. Crop Sci. 187: 231-239; Sciuchetti (1964) J. Pharm. Sci. 53: 61-69; Shipley (2002) Funct. Ecol. 16: 682-689; Singh et al. (2009) Sci. Hortic. 120: 173-180; Slauenwhite & Qaderi (2013) J. Agron. Crop Sci. 199: 286-298; Smith et al. (1987) Funct. Ecol. 1: 139-143; Snyder & Bunce (1983) Ann. Bot. 52: 895-903; Sobrado & Turner (1986) Oecologia 69: 181-187; Song et al. (2009); Song et al. (2010) Acta Oecol. 35: 128-135; Biol. Invasions 12: 1221-1230; Srutek (1995) Can. J. Bot. 73: 843-851; Stienstra (1986) Plant Cell Environ. 9: 307-313; Talwar et al. (1999) Crop Sci. 39: 460-466; Taub (2002) Can. J. Bot. 80: 34-41; Taylor et al. (2010) New Phytol. 185: 780-791; Tholen D. (unpubl.); Thomas & Strain (1991) Plant Physiol. 96: 627-634; Tognoni et al. (1967) Planta 72: 43-52; Tremmel & Patterson (1993) Can. J. Plant Sci. 73: 1249-1260; Turner & Lahav (1983) Aust. J. Plant Physiol. 10: 43-53;

U-Z

Van Arendonk et al. (1997) Plant Cell Environ. 20: 881-897; Van de Staaij et al. (1993) Vegetatio 104/105: 433-439; Van de Staaij et al. (1995) Environ. Poll. 90: 357-362; Van den Boogaard et al. (1997) Plant Cell Environ. 20: 200-210; Van der Werf :: AB-DLO report 207, WUR; Van der Werf et al. (1993a,b) Oecologia 94: 434-440; Physiol. Plant. 89:; Van der Werf et al. (1998) Inherent Variation in Plant Growth, pp. 489-502; Van Dobben et al. (1981) Acta Bot. Neerl. 30: 33-45; Veneklaas et al. (2002) Sci. Hortic. 93: 75-84; Venema et al. (2008) Env. Exp. Bot. 63: 359-367; Vessey et al. (1988) Biotronics 17: 79-94; Villar et al. (2005) Plant Soil 272: 11-27; Visser et al. (1997a) Plant Cell Environ. 20: 189-199; Volin & Reich (1996) Physiol. Plant. 97: 674-684; Volin et al. (1998) New Phytol. 138: 315-325; Wang & Han (2007) Soil Sci. Plant Nutr. 53: 278-285; Wang et al. (2007) Photosynthetica 46: 17-20; Watling & Press (1997) Plant Cell Environ. 20: 1292-1300; Watling & Press (1998) New Phytol. 140: 667-675; Welfare et al. (2002) Environ. Poll. 120: 397-403; Williams & Black (1993) Funct. Ecol. 7: 623-633; Williams & Black (1994) Oecologia 97: 512-519; Wyse (1980) Crop Sci. 20: 456-458; Yamauchi et al. (1988) Jap. J. Crop Sci. 57: 174-183; Zavala & Ravetta (2002) Plant Ecol. 161: 185-191; Zheng

et al. (2009) Plant Ecol. 203: 263-271; Ziska & Bunce (1994) Physiol. Plant. 91: 183-190; Ziska & Bunce (1997) Photosynth. Res. 54: 199-208; Ziska & Bunce (1999) Aust. J. Plant Physiol. 26: 71-77; Ziska (2003) J. Exp. Bot. 54: 395-404; Ziska et al. (1991) Oecologia 86: 383-389; Ziska et al. (1995) Physiol. Plant. 95: 355-364; Ziska et al. (1999) Physiol. Plant. 105: 74-80; Zoghlami et al. (2011) Afr. J. Biotechn. 10: 567-579; Zribi et al. (2009) Sci. Hortic. 120: 367-372;

Woody species:

A-E

Abrazhko (1973) & Alexeyev et al. (1973) Structure and productivity of spruce forests on southern taiga.; Adams (1928) Vermont Agric. Axp. Stn Bull. 282; Adams et al. (1990) New Phytol. 116: 689-694; Adegbidi et al. (2005) For. Ecol. Man. 218: 245-258; Ai & Shen (2001) J. Hubei Inst. Natl. (Nat. Sci.) 19: 20-22 (in Chinese); Ai & Zhou (1996) Hubei For. Sci. Technol.: 17-20 (in Chinese); Alameda & Villar (2009) Soil Till. Res. 103: 325-331; Alameda & Villar (2012) Env. Exp. Bot. 79: 49-57; Alameda et al. (2012) Soil Till. Res. 120: 121-129; Alban et al. (1978) From: Cannell (1982); Albaugh et al. (1998) For. Sci. 44: 317-328; Albrektson (1980a,b) Dept. of Silviculture. Report No. 2. Umea, Sweden; Ecol. Bull 32: 315-315; Alexeyev (1967) Reports of Academy of Sci. USSR. Vol. 175. No. 4. p 954-957; Ali (2000) Plant Sci. 152: 173-179; Almeida et al. (1999) Plant Soil 210: 159-166; Ammer (2003) Ann. Sci. For. 60: 163-171; An et al. (1991) Guizhou For. Sci. Technol. 19: 20-34 (in Chinese); Anderson & Tomlinson (1998) New Phytol. 140: 477-491; Andersson (1970) Botaniska Notiser. 123: 8-51; Ando & Takeuchi (1973) From: Cannell (1982); Ando (1965) & Kabaya et al. (1964) Advg. Front. Pl. Sci., New Delhi. Vol. 10. p. 1-10 & Bull. Marine Lab. Chiba Univ. 6: 1-26.; Ando et al. (1977) From: Cannell (1982); Ando et al. (1977) In: Primary Productivity in Forests, pp 213-245; Andrushenko (1976; 1977) Transactions of Khar¢kov Agricultural Institute. Vol. 225: 52-59 & 240: 53-56; Antonenko et al. (1982) Structure and functioning of the southern taiga geosystems at the Priirtysh'ye region. p 36-108; Antonov (1986) Gorskostopanska Nauka (Forest Sci.) Vol. 23: 43-51.; Antonov (1991) Gorskostopanska Nauka (Forest Sci.) No. 1: 55-61.; Anufrieva (1976) Lesovedeniye I Lesnoye Khozyaistvo (Forestry and Forest Management) 11: 106-113; Aranda et al. (2005) For. Ecol. Manage. 210: 117-129; Arnone & Gordon (1990) New Phytol. 116: 55-66; Ashton (1976) From: Cannell (1982); Ashton et al (1999) For. Sci. 45: 512-519; Askarov (1974) Ph.D. Thesis. Alma-Ata: Kazskhi, 1974.; Astrologova (1978) Lesnoi Zhurnal (Forest Journ.) 2: 16-20.; Atkin (1978) Lesovedeniye (Rus. For. Sci.) 5: 61-66.; Atkin et al. (1999) Oecologia 120: 544-554; Atkinson et al. (1997) Tree Physiol. 17: 319-325; Babich et al. (2004) Arkhangelsk: State Technical University, 112 p. ; Babich et al. (2004); Merzlenko (1986) Arkhangelsk: State Technical University, 112 p. & Vestnik of Agricultural Sciences 6: 145-147; Bacelar et al. (2007) Env. Exp. Bot. 60: 183-192; Balachandran et al. (1997) Plant Physiol. 114: 475-481; Balboa-Murias et al. (2006) Ann. For. Sci. 63: 557-565; Ball & Pidsley (1995) Funct. Ecol. 9: 77-85; Ball (1988) Aust. J. Plant Physiol. 15: 447-464; Ball (2002) Trees 16: 126-139; Bao et al. (1984) Acta Phytoecol. Geobot. Sin. 8: 313-320 (in Chinese); Barrett & Ash (1992) Aust. J. Bot. 40: 13-25; Bar-Tal et al. (1995) Sci. Hortic. 63: 195-208; Baruch et al. (2000) Int. J. Plant Sci. 161: 107-118; Baskerville (1965; 1966) From: Cannell (1982); Basov (1979) Bull. VNIALMI. 30: 21-25; BassiriRad et al. (1997) Aust. J. Plant Physiol. 24: 353-358; Bassman et al. (2001) Int. J. Plant Sci. 162: 103-110; Bassow et al. (1994) Ecol. Appl. 4: 593-603; Bazilevich (1967b) Rastitelnye Resursy (Plant Resources) 3: 567-588; Bencat (1988; 1989) Lesnictvi 34:

51-60 & Black locust biomass production in Southern Slovakia. Bratislava: VEDA, 191 p.; Bernardo et al. (1998) For. Ecol. Man. 104: 1-13; Berryman et al. (1993) Aust. J. Bot. 41: 195-209; Bert & Danjon (2006) Forest Ecol. Manage. 222: 279-295; Bieleski (1959) Aust. J. Bot. 7: 279-294; Biological production... (1978) JIBP Synthesis. University of Tokyo Press. Vol. 18.; Biskupsky (1981) From: Cannell (1982); Black et al. (2009) Forestry 82: 255-272; Bloomberg et al. (2008) New For. 36: 103-114; Blouin et al. (2008) For. Ecol. Manage. 255: 2444-2452; Bobkova & Tuzhilkina (2006b); Bobkova (2007) Natural spruce forests of the Russian North: Biodiversity, structure, functions. St.-Peterburg: Nauka. p. 265-288 & Lesovedeniye (Rus. For. Sci.) 6: 45-54; Bobkova (1987) Biological productivity of coniferous forests on European North-East. Leningrad: Nauka, 156 p.; Bobkova (1987; 2005) Biological productivity of coniferous forests on European North-East. Leningrad: Nauka, 156 p.; Bobkova (2001) Bioproductive process in forest ecosystems of Russian North. St.-Petersburg: Nauka, p 52-68; Bobkova (2005) Lesovedeniye (Rus. For. Sci.) 6: 30-37; Bobkova et al. (2006); Bobkova & Tuzhilkina (2006); Bobkova (2007) Ecologia (Rus. J. Ecology) 1: 23-31 & Natural spruce forests of the Russian North: Biodiversity, structure, functions. St.-Peterburg: Nauka. P 265-288 & Lesovedeniye (Rus. For. Sci.) 6: 45-54; Bongers et al. (1988) Funct. Ecol. 2: 379-390; Bonkowski et al. (2001) Appl. Soil. Ecol. 18: 193-204; Boutraa et al. (2010) Res. J. Agric. Biol. Sci. 6: 20-26; Boyko et al. (1970; 1975b) Berezina reserve: Studies. 1970. Issue 1. Minsk. P 51-63 & Experimental studies of natural complexes in Berezina reserve. Minsk: Nauka i Tekhnika, 376 p.; Boyko et al. (1975b) Experimental studies of natural complexes in Berezina reserve. Minsk: Nauka i Tekhnika, 376 p.; Boyko et al. (1976); Boyko & Loznukho (1982) Experimental studies on landscapes of Pripyatskii reserve. Minsk: Nauka i Tekhnika, 304 p. & Bio-ecological peculiarities of forest phytocoenoses of Pripyatski reserve. Minsk: Nauka i Tekhnika, 134 p. ; Boyko et al. (1976); Boyko & Loznukho (1982) Experimental studies on landscapes of Pripyatskii reserve. Minsk: Nauka i Tekhnika, 304 p. & Bio-ecological peculiarities of forest phytocoenoses of Pripyatski reserve. Minsk: Nauka i Tekhnika, 134 p. ; Boyko et al. (1976); Boyko & Loznukho (1982); Kirkovski,1986 Experimental studies on landscapes of Pripyatskii reserve. Minsk: Nauka i Tekhnika, 304 p. & Bio-ecological peculiarities of forest phytocoenoses of Pripyatski reserve. Minsk: Nauka i Tekhnika, 134 p. & Botany: Studies. Issue 27. Minsk: Nauka i Tekhnika. P. 170-172; Boyko et al. (1983) Ecologic and phytocoenotic studies of forest vegetation in Nalibokskaya Pushcha // Minsk: Nauka i Tekhnika, 208 p.; Bradbury (1990) J. Arid Environ. 18: 325-333; Brix (1971) Can. J. Bot. 49: 289-294; Broshtilova (1986) Forest Sci. (Sofia) 23: 59-66; Brown & Higginbotham (1986) Tree Physiol. 2: 223-232; Bruhn et al. (2000) New Phytol. 146: 415-425; Bruno et al. (1976; 1977) Annali di Botanica (Roma) 35-36: 109-118.; Bryla et al. (1997) Plant Cell Environ. 20: 1411-1420; Bugaev & Onishchenko (1987) Lesnoi Zhurnal (Forest Journal) 6: 15-17; Bungard et al. (1997) Aust. J. Plant Physiol. 24: 205-214; Burchett et al. (1984) Physiol. Plant. 60: 113-118; Burrows et al. (2001) NCAS techical report 33; Burslem et al. (1995) J. Ecol. 83: 113-122; Burslem et al. (1996) Biotropica 28: 636-648; Buwalda & Lenz (1992) Physiol. Plant. 84: 21-28; Buzykin & Pshenichnikova (1978); Pshenichnikova (1978) Pine forest productivity. Moscow: Nauka, P. 45-68 & 69-89; Cabanettes & Rapp (1978) From: Cannell (1982); Cai et al. (1991) J. Northeast For. Univ. 19: 331-336 (in Chinese); Calamini et al. (1983; 1989) Annali Istituto Sperimentale Studio e Difesa del Suolo, Firenze 14: 193-214 & Annali Accademia Italiana di Scienze Forestali 37: 373-399; Callaway et al. (1994) Oecologia 98: 159-166; Canham et al. (1996) Can. J. For. Res. 26: 1521-1530; Cao et al. (2008) For. Ecol. Manage. 254: 217-224; Capon et al. (2009) Env. Exp. Bot. 66: 178-185; Carey & O'Brien (1979) Irish For. 1: 25-35; Carswell et al. (2000) Tree Physiol. 20: 977-986;

Castro-Diez et al. (2006) Tree Physiol. 26: 389-400; Centritto (2000) Plant Biosyst. 134: 31-37; Centritto et al. (1999) Tree Physiol. 19: 799-806; Cermák et al. (1998); Xiao et al. (2003) Ann. Sci. For. 55: 63-88 & Tree Physiol. 23: 505-516; Cernusak et al. (2009) Plant Cell Environ. 32: 1441-1455; Ceulemans et al. (1999) Ann. Bot. 84: 577-590; Chambel et al. (2007) Ann. For. Sci. 64: 87-97; Chan et al. (2003) Can. J. For. Res. 33: 106-117; Chang et al. (1997) J. Northwest For. Coll. 12: 23-28 (in Chinese); Chappelka et al. (1985) Env. Exp. Bot. 25: 233-244; Chatzisthatis et al. (2009) HortScience 44: 1994-1999; Chazdon (1986) Am. Nat. 127: 9-30; Chen & Chen (1980) Sci. Silvae Sin.: 269-278 (in Chinese); Chen (2001) China For. Sci. Technol. 15: 24-26 (in Chinese); Chen (2006a) Fujian Agr. Sci. Technol.: 61-63 (in Chinese); Chen (2006b) J. Jiangsu For. Sci. Technol. 33: 23-24, 49 (in Chinese); Chen et al. (1982) J. Northwest For. Coll. 11: 103-114 (in Chinese); Chen et al. (1991) J. Shanxi Agr. Univ. 11: 240-247 (in Chinese); Chen et al. (1994) J. Fujian For. Sci. Technol. 21: 19-23 (in Chinese); Chen et al. (1996) J. Northwest For. Coll. 11: 103-114 (in Chinese); Chen et al. (2000a) J. Zhejiang For. Coll. 17: 369-372 (in Chinese); Chen et al. (2000b) J. Fujian Coll. For. 20: 309-312 (in Chinese); Chen et al. (2001a) J. Cent. South For. Univ. 21: 44-47 (in Chinese); Chen et al. (2001b) J. Fujian Coll. For. 21: 113-115 (in Chinese); Chen et al. (2005) Ann. For. Sci. 62: 659-668; Cheng & Li (1989) Inner Mongolian For. Invest. Des. 7: 29-39; Cheng & Li (1989) Inner Mongolian For. Invest. Des. 7: 29-40; Cheng & Li (1989) Inner Mongolian For. Invest. Des. 7: 29-41; Cheng & Li (1989) Inner Mongolian For. Invest. Des. 7: 29-42; Cheng & Li (1989) Inner Mongolian For. Invest. Des. 7: 29-43; Cheng & Li (1989) Inner Mongolian For. Invest. Des. 7: 29-44; Cheng & Li (1989) Inner Mongolian For. Invest. Des. 7: 29-45; Cheng & Li (1989) Inner Mongolian For. Invest. Des. 7: 29-46; Cheng & Li (1989) Inner Mongolian For. Invest. Des. 7: 29-47; Cheng & Li (1989) Inner Mongolian For. Invest. Des. 7: 29-48; Cheng & Li (1989) Inner Mongolian For. Invest. Des. 7: 29-49; Cheng & Li (1989) Inner Mongolian For. Invest. Des. 7: 29-50; Cheng & Li (1989) Inner Mongolian For. Invest. Des. 7: 29-51; Cheng & Li (1989) Inner Mongolian For. Invest. Des. 7: 29-52; Cheng et al. (2013) J. Trop. For. Sci. 25: 537-546; Chepurko (1971; 1972) Biological productivity and chemical element cycling in plant communities. Leningrad: Nauka, p 213-219 & Soils and plant community productivity. Issue 1. Moscow: State University, 1972. P. 94-116; Chepurko (1971; 1972); Manakov & Nikonov (1979) Biological productivity and chemical element cycling in plant communities. Leningrad: Nauka, p 213-219 & Soils and plant community productivity. Issue 1. Moscow: State University, 1972. P. 94-116 & Botan. Zhurnal (Rus. Bot. Journal) 64: 232-241; Chertovskoi et al. (1978) Ecology of taiga forests. Arkhangelsk: Forestry and Wood Chemistry Institute. P. 32-42; Chew & Chew (1965) Ecol. Monogr. 35: 355-375; Chibisov (1992) Increase of forest productivity on European North. Arkhangelsk: SevNIILKh, P. 34-37; Chibisov (1992) Increase of forest productivity on European North. Arkhangelsk: SevNIILKh, P. 34-38; Chibisov (1992) Increase of forest productivity on European North. Arkhangelsk: SevNIILKh, P. 34-39; Chibisov (1992) Increase of forest productivity on European North. Arkhangelsk: SevNIILKh, P. 34-40; Chibisov (1992) Increase of forest productivity on European North. Arkhangelsk: SevNIILKh, P. 34-41; Chibisov (1992) Increase of forest productivity on European North. Arkhangelsk: SevNIILKh, P. 34-42; Chibisov (1997) Lesnoi Zhurnal (Forest Journ.) 5: 7-16; Chibisov (1997) Lesnoi Zhurnal (Forest Journ.) 5: 7-17; Chibisov (1997) Lesnoi Zhurnal (Forest Journ.) 5: 7-18; Chibisov et al. (1978; 1980) Material of annual session in 1977. Arkhangelsk, P. 25-26 & Thinnings and clear cuttings on European North. Arkhangelsk: Institute of Forest and Forest Chemie, P. 6-29; Chibisov et al. (1978; 1980) Material of annual session in 1977. Arkhangelsk, P. 25-26 & Thinnings and clear cuttings on European North. Arkhangelsk: Institute of Forest and Forest Chemie, P. 6-30; Chibisov et al.

(1978; 1980) Material of annual session in 1977. Arkhangelsk, P. 25-26 & Thinnings and clear cuttings on European North. Arkhangelsk: Institute of Forest and Forest Chemie, P. 6-31; Chirino et al. (2008) For. Ecol. Manage. 256: 779-785; Chmura et al. (2014) Scand. J. For. Res. (in press); Climent et al. (2008) Silvae Genet. 57: 187-193; Climent et al. (2011) Eur. J. For. Res. 130: 841-850; Clough et al. (1979b) Plant Physiol. 64: 25-30; Comeau & Kimmins (1989a) For. Ecol. Man. 255: 2502-2511; Comeau & Kimmins (1989b) Can. J. For. Res. 19: 447-454; Condon et al. (1992) Funct. Ecol. 6: 680-685; Conroy et al. (1986) Ann. Bot. 57: 165-177; Conroy et al. (1990) Plant Cell Environ. 13: 329-337; Cordero (1999) Tree Physiol. 19: 153-163; Corley & Tinker (2003) In: The Oil Palm; Corley et al. (1971) Expl. Agric. 7: 129-136; Cornelissen et al. (1996) J. Ecol. 84: 755-765; Cornelissen et al. (1999) New Phytol. 141: 401-409; Cortina et al. (2008) Env. Exp. Bot. 62: 343-350; Costa et al. (2004) Tree Physiol. 24: 1165 - 1172; Cubera et al. (2009) Soil Till. Res. 103: 16-22; Curriel Yuste et al. (2005) Tree Physiol. 25: 701-712; Damesin et al. (1996) Ann. Sci. For. 53: 461-467; Dang & Wu (1991) Acta Bot. Yunnan 13: 59-64 (in Chinese); Dang & Wu (1992) J. Yunnan Univ. (Nat. Sci.) 14: 95-107 (in Chinese); Dang et al. (1994a) J. Yunnan Univ. (Nat. Sci.) 16: 205-209 (in Chinese); Dang et al. (1994b) J. Yunnan Univ. (Nat. Sci.) 16: 214-219 (in Chinese); De Groot et al. (2001) Plant Cell Environ. 24: 1309-1317; De la Rosa et al. (1998) Plant Soil 201: 17-25; De la Rosa et al. (2003) Glob. Change Biol. 9: 65-73; De Oliveira et al. (2012) Env. Exp. Bot. 77: 53-62; DeAngelis et al. (1981) Dynamic properties of forest ecosystems. IBP-23. Cambridge: Univ. Press, P. 567-672.; Debez et al. (2004,2006) Plant Soil 262: 179-189; Env. Exp. Bot. 57: 285-295; DeLucia et al. (1994) Tree Physiol. 14:669-677; DeLucia et al. (1998) Am. J. Bot. 85: 955-963; Deng et al. (1988) Chinese J. Ecol. 7: 13-18 (in Chinese); Devillez et al. (1973b) Bulletin de la Classe des Sciences. Acad. Royale de Belgique, Bruxelles. 5e série. Tome 59. P. 303-331.; Devillez et al. (1973a) Bull. de la Classe des Sciences. Academie Royale de Belgique, Bruxelles. 1973a. 5 serie. Vol. 59. P. 480-491.; Deyeva (1985; 1987) Botan. Zhurnal (Rus. Bot. Journ) 70: 54-58 & 72: 505-511; Di et al. (1991) J. Nanjing For. Univ. 15: 60-65 (in Chinese); Diakonov & Reteyum (1971) Biological productivity and cycling chemical elements in plant communities. Leningrad: Nauka, 1971. P. 43-46 (Rus.).; Dias et al. (2007) J. Plant Physiol. 164: 1639-1647; Diaz-Lopez et al. (2012) Agric. Water Manage. 105: 48-56; Ding & Sun (1989) J. Northeast For. Univ. 17(S): 1-98 (in Chinese); Ding & Wang (2001) For. Res. 15: 54-60 (in Chinese); Ding (2000) J. Mt. Agr. Biol. 19: 411-417 (in Chinese); Ding (2003) J. Fujian Coll. For. 23: 34-38 (in Chinese); Ding et al. (1990) Acta Phytoecol. Geobot. Sin. 14: 226-236 (in Chinese); Doley (1978) Aust. J. Plant Physiol. 5: 723-738; Domisch et al. (2001) Tree Physiol. 21: 465-472; Dong (2000) J. Chuzhou Norm. Coll. 2: 95-97, 80 (in Chinese); Downton & Grant (1994) Aust. J. Plant Physiol. 21: 273-279; Downton et al. (1990) Sci. Hortic. 44: 215-225; Droste zu Hülshoff (1969) Dissertation. München, 222 S.; Dueck et al. (1998) Atmos. Environ. 32: 545-550; Dufrene (1989) In: The Oil Palm; Duvigneaud et al. (1971); Duvigneaud & Denaeyer–De Smet (1967) Productivity of forest ecosystems (P. Duvigneaud, ed.). UNESCO: Paris, 1971. P. 259-270 & Symposium on primary productivity and mineral cycling in natural ecosystems. University of Maine, USA. 1967. P. 167 – 186.; Duvigneaud et al. (1977a) Productivité biologique en Belgique (P. Duvigneaud and P. Kestemont, eds.). SCOPE: Editions Duculot, Paris – Gembloux, 1977a. P. 489-500.; Duvigneaud et al. (1977ab) Productivité biologique en Belgique (P. Duvigneaud and P. Kestemont, eds.). SCOPE: Editions Duculot, Paris – Gembloux, 1977a. P. 489-500 & p. 107-154; Dylis & Nosova (1977); Kashlev (1968) Phytomass of forest biogeocoenoses on localities near Moscow. Moscow: Nauka, 1977. 143 p. & Reports of Timiryazev Agricult. Academy. Issue 139. 1968. P. 249-252 (Rus.).; Dylis & Nosova (1977); Lozinov (1980) Phytomass of forest

biogeocoenoses on localities near Moscow. Moscow: Nauka, 1977. 143 p. & Reports of Timiryazev Agricult. Academy. Issue 139. 1968. P. 249-252 (Rus.).; Dzens-Litovskaya (1960) Vestnik Leningrad State University. Geological and Geographical Series. 1960. Issue 12. No. 2. P. 110-126 (Rus.).; Egilla et al. (2001) Plant Soil 229: 213-224; Egunjobi (1975; 1976) From: Cannell (1982); El Kohen et al. (1992) Ann. Sci. For. 49: 83-90; Elkington & Jones (1974) J. Ecol. 62: 821-830; Ellenberg et al. (1986) Ökosystemforschung - Ergebnisse des Sollingprojekts: 1966-1986. Stuttgart: Verlag Ulmer, P 1-507; Enright (1985) Aust. J. Ecol. 10: 461-467; Entry et al. (1998) Plant Soil 200: 3-11; Equiza et al. (2005) Tree Physiol. 26: 353-364; Ermakov & Asyutin (1988) Lesovedeniye I Lesnoye Khozyaistvo (Forestry and Forest Management). Issue 23. Minsk, 1988. P. 74-76 (Rus.).; Ermolenko & Ermolenko (1982) Forming and productivity of forest phytocoenoses. Krasnoyarsk: Sukachev Institute, 1982. P. 60-71 (Rus.).; Ernst et al. (1997) Plant Ecol. 128: 163-170; Evans & Poorter (2001) Plant Cell Environ. 24: 755-767; Everett et al. (2010) J. Plant Nutr. 33: 1638-1657;

F-J

Fan et al. (1996ab) For. Res. 9: 50-54 (in Chinese); For. Res. 9: 78-85 (in Chinese); Fan et al. (1997) J. Beijing For. Univ. 19: 93-98 (in Chinese); Fan et al. (2005); Fan et al. (2006) Chinese J. Appl. Environ. Biol. 11: 521-527 (in Chinese); Acta Ecol. Sin. 26: 2463-2473 (in Chinese); Fang & Kong (2003) J. Fujian Coll. For. 23: 182-185 (in Chinese); Fang & Tian (2006) Guihaia 26: 516-522 (in Chinese); Fang (1990) Sci. Silvae Sin. 26: 201-208 (in Chinese); Fang et al. (1991) J. Qinghai Univ. 9: 71-77 (in Chinese); Fang et al. (1996) Tree Physiol. 16: 441-446; Fang et al. (1999) J. Cent. South For. Univ. 19: 16-19 (in Chinese); Fang et al. (2002) Sci. Silvae Sin. 38: 14-19 (in Chinese); Fang et al. (2003a) J. Cent. South For. Univ. 23: 11-15 (in Chinese); Fang et al. (2003b) J. Zhejiang For. Coll. 20: 374-379 (in Chinese); Feger et al. (1991) Forstwissenschaftliches Zentralblatt. 110: 248-262.; Feng & Yang (1981) J. Inner Mongolia For. Coll.: 1-17 (in Chinese); Feng & Yang (1985) Sci. Silvae Sin. 21: 86-92 (in Chinese); Feng & Yang (1985; 1995) Scientia Silvae Sinicae. 1985. Vol. 21. No.1. P. 86-92 (Chin.) & USDA Forest Service, Intermountain Research Station. GTR-INT-319 (Ecology and Management of Larix forests: A look ahead. Proc. Intern. Symp.). 1995. P. 240-243.; Feng (1980) Comprehensive Investigation Reports of Taoyuan County (eds Taoyuan Agricultural Experiment Station of Chinese Academy of Sciences): 322-333 (Hunan Science and Technology Press, 1980) (in Chinese); Feng et al. (1982a) Sci. Silvae Sin. 18: 127-134 (in Chinese); Feng et al. (1982b) J. Nanjing Technol. Coll. For. Prod.: 19-38 (in Chinese); Feng et al. (1983) J. Northeast. For. Inst. 11: 13-20 (in Chinese); Feng et al. (1984) Acta Phytoecol. Geobot. Sin. 8: 93-100 (in Chinese); Feng et al. (1999) J. Guangxi Acad. Sci. 15: 94-96; Feng et al. (2007) Acta Oecol. 31: 40-47; Ferree et al. (2004) HortScience 39: 40-48; Ferreira et al. (2007) Env. Exp. Bot. 60: 477-483; Ferreira et al. (2012) Acta Bot. Bras. 26: 408-414; Fetcher et al. (1983) Oecologia 58: 314-319; Fine et al. (2013) Int. J. Phytorem. 15: 585-601; Ford (1982) Forestry 55: 1-17; Foruqi (1981) From: Cannell (1982); Fownes & Harrington (2004) For. Ecol. Manage. 203: 297-310; Freitas et al. (2006) Servicios ambientales de almacenamiento y secuestro de carbono del ecosistema aguajal en la reserva nacional pacaya samiria, Loreto, Peru; Frey & Koppel (1983); Frey (1977) Horisont. 12: 13-18 (Eston.) & Spruce Forest Ecosystem Structure and Ecology. Vol. 1. (Estonian IBP Report, 11). Tartu, 1977. P. 21-36.; Fu (1989) J. Cent. South For. Univ. 9: 76-84 (in Chinese); Fukui (2000) Plant Prod. Sci. 3: 404-409; Gabeyev (1968; 1969; 1976; 1990) Izv. Sib. Otdel. AS. USSR. Biol. and Medicine Series. 1968. Vol. 15. No. 3. P. 98-106 (Rus.) & Lesovedeniye (Rus. For. Sci.). 1969. No. 3. P. 75-78 (Rus.) &

Biological productivity of Priob'ye region forests. Novosibirsk: Nauka, 1976. 171 p. (Rus.) & Ecology and productivity of pine forests. Novosibirsk: Nauka, 1990. 229 p. (Rus.).; Gabeyev (1968; 1976; 1990) Izv. Sib. Otdel. AS. USSR. Biol. and Medicine Series. 1968. Vol. 15. No. 3. P. 98-106 (Rus.) & Biological productivity of Priob'ye region forests. Novosibirsk: Nauka, 1976. 171 p. (Rus.) & Ecology and productivity of pine forests. Novosibirsk: Nauka, 1990. 229 p. (Rus.).; Gabeyev (1976) Biological productivity of Priob'ye region forests. Novosibirsk: Nauka, 1976. 171 p. (Rus.).; Gao & Xiao (2001) Chinese J. Appl. Ecol. 12: 667-671 (in Chinese); Gao (1986) J. Zhejiang For. Sci. Technol.: 25-30 (in Chinese); Gao et al. (1992) Acta Phytoecol. Geobot. Sin. 16: 64-71 (in Chinese); Gao et al. (2002) J. Beijing For. Univ. 24: 26-30 (in Chinese); Garcia-Moya & McKell (1970) Ecology 51: 81-88; Garelkov (1970) Gorskostopanska Nauka (Forest Sci.) 7: 39-58; Garelkov (1973) From: Cannell (1982); Garkoti (2008) Ecol. Res. 23: 41-49; Garrish et al. (2010) J. Exp. Bot. 61: 3735-3748; Gezelius & Nasholm (1993) Tree Physiol. 13: 71-86; Ghannoum et al. (2010) Glob. Change Biol. 16: 303-319; Gifford (1991) NERDDC report 1402; Gleadow et al. (1998) Plant Cell Environ. 21: 12-22; Gleason & Ares (2004) Tree Physiol. 24: 1087-1097; Glynn et al. (2003) Oikos 101: 385-397; Golley et al. (1962) From: Cannell (1982); Golley et al. (1971;1975) From: Cannell (1982); Golovenko et al. (1976; 1981); Gerasimova et al. (1980) Soils and plant community productivity. Issue 3. Moscow: State University, 1976. P. 73-96 & Soils and plant community productivity. Issue 5. Moscow: State University, 1981. P. 160-193 & Forest ecology and productivity of Nechernozemye region. Moscow: State University, P. 84-138; Golubets & Polovnikov (1975) Biological productivity of Carpatian spruce forests. Kiev: Naukova Dumka, P. 4-68; Golubets & Polovnikov (1975) Biological productivity of Carpatian spruce forests. Kiev: Naukova Dumka, P. 4-69; Golubets & Polovnikov (1975) Biological productivity of Carpatian spruce forests. Kiev: Naukova Dumka, P. 4-70; Golubets & Polovnikov (1975) Biological productivity of Carpatian spruce forests. Kiev: Naukova Dumka, P. 4-71; Golubets & Polovnikov (1975) Biological productivity of Carpatian spruce forests. Kiev: Naukova Dumka, P. 4-72; Golubets & Polovnikov (1975) Biological productivity of Carpatian spruce forests. Kiev: Naukova Dumka, P. 4-73; Golubets & Polovnikov (1975) Biological productivity of Carpatian spruce forests. Kiev: Naukova Dumka, P. 4-74; Golubets & Polovnikov (1975) Biological productivity of Carpatian spruce forests. Kiev: Naukova Dumka, P. 4-75; Golubets & Polovnikov (1975) Biological productivity of Carpatian spruce forests. Kiev: Naukova Dumka, P. 4-76; Golubets & Polovnikov (1975) Biological productivity of Carpatian spruce forests. Kiev: Naukova Dumka, P. 4-77; Golubets & Polovnikov (1975) Biological productivity of Carpatian spruce forests. Kiev: Naukova Dumka, P. 4-78; Golubets & Polovnikov (1975) Biological productivity of Carpatian spruce forests. Kiev: Naukova Dumka, P. 4-79; Golubets & Polovnikov (1975) Biological productivity of Carpatian spruce forests. Kiev: Naukova Dumka, P. 4-80; Golubets & Polovnikov (1975) Biological productivity of Carpatian spruce forests. Kiev: Naukova Dumka, P. 4-81; Golubets & Polovnikov (1975) Biological productivity of Carpatian spruce forests. Kiev: Naukova Dumka, P. 4-82; Golubets & Polovnikov (1975) Biological productivity of Carpatian spruce forests. Kiev: Naukova Dumka, P. 4-83; Gordina (1979) Dissertation. Krasnoyarsk: Siberian Technolog. Institute, 139 p. (Siberian Technological University library); Gordon (1981) From: Cannell (1982); Gortinski et al. (1975) Biospheric resources: Synthesis of the Soviet studies for the International Biological Programme. Volume 1. Leningrad: Nauka, P. 34-42; Gottschalk (1994) Tree Physiol. 14: 735-749; Goudriaan & De Ruiter (1983) Neth. J. Agric. Sci. 31: 157-169; Govorenkov (1972) Geography, Genesis and Soil Fertility: Transactions of Dokuchaev Central Soil Science Museum. Issue 5. Leningrad. P. 103-130; Gower et al. (1992) Ecol. Monogr. 62: 43-65; Grantz

et al. (2010) Plant Cell Environ. 33: 717-728; Grassi & Minotta (2000) Tree Physiol. 20: 645-652; Grechi et al. (2007) Env. Exp. Bot. 59: 139-149; Greshilova (2004); Stakanov (1990); Stakanov et al. (2002) Dissertation. Krasnoyarsk: Sukachev Forest Institute, 2004. 175 p. (V.N. Sukachev Institute library) & Lesovedeniye (Rus. For. Sci.) 4: 25-33 & Lesnoye Khozyaistvo (Rus. Forest Management) 2: 24-25; Grier et al. (1981) Can. J. For. Res. 11: 155-167; Griffin et al. (1995, 1996) New Phytol. 129: 547-556; Plant Cell Env. 19: 729-738; Groninger et al. (1996) Tree Physiol. 16: 773-778; Grozeva et al. (1986) Gorskostopanska Nauka (Forest Sci.) 23: 52-58; Grubb et al. (1996) J. Ecol. 84: 827-840; Gruk (1979) Lesovedeniye I Lesnoye Khozyaistvo (Forestry and Forest Management) 14: 56-59; Guehl et al. (1994) Tree Physiol. 14: 707-724; Guo (2003) China For. Sci. Technol. 17(S): 28-30 (in Chinese); Guo (2006) East China For. Manag. 20: 24-26 (in Chinese); Haggar & Ewel (1994) Interciencia 19: 347-351; Hale et al. (2005) J. Chem. Ecol. 31: 2601-2620; Han (1994) Long-term Research on China's Forest Ecosystems (eds Department of Science and Technology, Ministry of Forestry): 451-458 (Northeast Forestry University Press, 1994); Han et al. (1997); Han & Liang (1997) J. Shanxi Agr. Univ. 17: 278-283 (in Chinese);Shanxi For. Sci. Technol.: 36-40 (in Chinese); Han et al. (2007) J. Northwest For. Univ. 22: 100-104 (in Chinese); Hanson et al. (1987) HortScience 22: 1293-1295; Harada et al. (1969) J. Jap. For. Soc. 51: 125-133.; Harada et al. (1969); Yamada & Shidei (1968) J. Jap. For. Soc. 51: 125-133. & Bull. Kyoto Univer. For. 40: 67-92; Harada et al. (1972) Bull. Government. Forest Exper. Station Tokyo 249: 17-74; Hatiya et al. (1965; 1968) Bull. Governm. Forest Exp. Station Tokyo 176: 75-88 & Primary productivity of artificially managed forests. Progress report for 1967 (ed. T. Satoo). P. 27-30; Hatiya et al. (1966) J. Jpn. For. Soc. 48: 445-448; Hattenschwiler & Korner (2003) Funct. Ecol. 17: 778-785; Havas & Kubin (1983) Ann. Bot. Fennici. 20: 115-149; Havas (1981) From: Cannell (1982); Hayashida Y. & Boot R.G.A. (unpubl).; He (1998) J. Cent. South For. Univ. 18: 53-59 (in Chinese); He et al. (2008) J. Northeast For. Univ. 36: 17-18, 27 (in Chinese); Helmisaari et al. (2002) For. Ecol. Man. 165: 317-326; Heriansyah et al. (2007) J. Trop. For. Sci. 19: 226-235; Hester et al. (2004) Oikos 105: 536-550; Hibbs et al. (1995) New Phytol. 129: 569-577; Hoad & Leaky (1994) For. Ecol. Manage. 70: 265-273; Hollinger (1987) Tree Physiol. 3: 193-202; Holm & Jensen (1981) From: Cannell (1982); Hovenden & Schimanski (2000) Aust. J. Plant Physiol. 27: 281-287; Hovenden (2001) Aust. J. Bot. 49: 427-434; Hsu et al. (1996) HortScience 31: 1139-1142; Hu et al. (1998) J. Cent. South For. Univ. 18: 60-64 (in Chinese); Hu et al. (1999) J. Liaoning For. Sci. Technol.: 13-16, 49 (in Chinese); Huang (1988) Cent. South For. Invent. Plan.: 13-21 (in Chinese); Huang (1998) J. Fujian For. Sci. Technol. 25: 20-23 (in Chinese); Huang (2006) J. Southwest For. Coll. 26: 15-18 (in Chinese); Huang et al. (2000) Jiangxi For. Sci. Technol.: 4-9 (in Chinese); Huang et al. (2007) Ecol. Environ. 16: 60-65 (in Chinese); Huante & Rincon (1998) Oecologia 113: 53-66; Huante et al. (1995b) Funct. Ecol. 9: 849-858; Huet et al. (2004) Ann. For. Sci. 61: 683-694; Hui et al. (1988) For. Res. 1: 413-417 (in Chinese); Hui et al. (1989) Sci. Silvae Sin. 25: 564-569 (in Chinese); Hurry et al. (1995) Plant Physiol. 109: 697-706; Ibrahim et al. (1997) Can. J. For. Res. 27: 1413-1419; Ibrahim et al. (1998) Tree Physiol. 18: 481-487; Il'yushenko & Koshel'kov (1977); Zvorykina (1977) Forestry studies on the southern taiga subzone. Moscow: Nauka, P. 32-41 & Forestry studies on the southern taiga subzone. Moscow: Nauka, P. 42-50; Il'yushenko (1968; 1970; 1982) Lesovedeniye (Rus. For. Sci.) 6: 65-67 & Forming tree annual ring and organic mass accumulating. Moscow: Nauka, P. 50-61 & Biological productivity of forests at Povolgie region. Moscow: Nauka, P. 73-98; Il'yushenko (1982) Biological productivity of forests at Povolgie region. Moscow: Nauka, P. 73-98; Imai & Murata (1976) Proc. Crop Sci. Soc. Jpn. 45: 598-606; Imai et al. (1984) Jap. J. Crop Sci. 53:

479-485; Isaac et al. (2007) Plant Soil 298: 243-254; Ivanchikov & Zyabchenko (1977) Biological and commercial productivity of forest phytocoenoses of Karelia. Petrozavodsk: Karelian Branch of AS USSR, P. 21-43; Ivanchikov (1971) Forest vegetation resources of the southern Karelia. Petrozavodsk: Karelia Publ. House, P. 78-85; Jagodzinski AM (unpubl.); Jakucs (1985) Ecology of an oak forest in Hungary. Results of «Sikfökút Project», 1: Structure, primary production and mineral cycling. Budapest: Akadémiai Kiadó, 1985. 546 p.; Jakucs (1986) Ecology of an oak forest in Hungary. Results of «Sikfökút Project», 1: Structure, primary production and mineral cycling. Budapest: Akadémiai Kiadó, 1985. 546 p.; James (2008) Plant Soil 310: 201-210; Janssens et al. (1999) Ann. For. Sci. 56: 81-90; Jarvis (1964) J. Ecol. 52: 545-571; Jia & Zhang (1985) J. Liaoning For. Sci. Technol.: 18-23 (in Chinese); Jiang & Zhao (1992) Proceedings of Forest Ecosystems on Xiashu Ecological Station (eds Jiang Z.L.): 10-15 (China Forestry Publishing House, 1992)(in Chinese); Jiang (1986) Acta Phytoecol. Geobot. Sin. 10: 146-152 (in Chinese); Jiao (1985) Acta Phytoecol. Geobot. Sin. 9: 257-265 (in Chinese); Johnson et al. (1997) Physiol. Plant. 101: 124-134; Johnstone (1972) From: Cannell (1982); Jomura et al. (2010) In: Permafrost Ecosystems, pp. 385-412; Jones et al. (1970) In: The Biology of Atriplex (Ed. R. Jones);

K-O

Kadeba & Aduayi (1986) Oikos 46: 237-242; Kajimoto et al. (1997); Kajimoto et al. (1999a) Proc. of the Fifth symposium on the joint Siberian permafrost studies between Japan and Russia in 1996. Japan, Tsukuba: National Inst. Environ. Studies, P. 119-128 & Tree Physiol. 19: 815-822; Kajimoto et al. (1998) Proc. Sixth symposium on the joint Siberian permafrost studies between Japan and Russia in 1997. Japan, Sapporo: Forestry and Forest Products Research Inst., P. 65-71.; Kajimoto et al. (1999) Tree Physiol. 19: 815-822; Kajimoto et al. (1999b) Proc. Fourth symposium on the joint Siberian permafrost studies between Japan and Russia in 1995. Japan, Sapporo: Hokkaido University. P. 71-77. ; Kajimoto et al. (2003) Plant Soil 255: 281-292; Kajimoto et al. (2010) In: Permafrost Ecosystems, pp. 99-122; Kakubari (1977) From: Cannell (1982); Kalapos et al. (1996) Plant Soil 185: 137-149; Kalinin (1983) Forming of tree root systems. Moscow: Lesnaya Promyshlennost, 152 p.; Kamenetskaya (1970); Kamenetskaya et al. (1973) Forming tree annual ring and organic mass accumulating. Moscow: Nauka, 1970. P. 62-83 & Productivity and structure of young Scots pine vegetation. Moscow: Nauka, P. 5-62. ; Kanazawa et al. (1994) Japan, Sapporo: Forestry and Forest Products Research Inst., P. 37-46.; Kang & Fang (1997) Sci. Silvae Sin. 33: 67-73 (in Chinese); Kang et al. (2004) Sci. Silvae Sin. 40: 205-209 (in Chinese); Kang et al. (2006) Acta Ecol. Sin. 26: 1320-1329 (in Chinese); Kanno et al. (2009) Soil Sci. Plant Nutr. 55: 124-131; Kapustinskaite (1976) Lesovedeniye (Rus. For. Sci.) 3: 20-30; Kapustinskaite (1978) Lesovedeniye (Rus. For. Sci.) 4: 22-29; Karaseva (2003) Larix sibirica on the Middle Povolzhye. Ioshkar-Ola: Mari State Technical University, 376 p.; Karizumi (1974) Bull. Gov. For. Exp. Sta. 259: 1-99; Karlsson & Nordell (1987) Funct. Ecol. 1: 37-44; Karnosky et al. (1996) Can. J. For. Res. 26: 23-37; Kawahara et al. (1979) Jap. J. Ecol. 29: 387-395; Kawanabe et al. (1975) From: Cannell (1982); Kawanabe et al. (1975b) J. Jap. For. Soc. 57: 215-223; Kazimirov & Morozova (1973) Biological cycling of matter in spruce forests of Karelia. Leningrad: Nauka, 175 p. ; Kazimirov et al. (1977) Turnover of matter and energy in pine forests of the European North. Leningrad: Nauka, 304 p.; Kazimirov et al. (1978) Organic mass and matter flows in birch stands of the middle taiga; Leningrad: Nauka, 216 p. ; Ke & Werger (1999) Acta Oecol. 20: 579-586; Kelly et al. (2009) For. Ecol. Manage. 257: 287-293; Kestemont (1975) & Duvigneaud & Kestemont (1977) From: Cannell (1982); Kestemont

(1975) From: Cannell (1982); Kestemont (1975); Duvigneaud & Kestemont (1977) From: Cannell (1982); Kestemont et al. (1977) Productivite biologique en Belgique. Paris: Duculot, P. 161-176.; Khalid et al. (1999ab) J. Oil Palm Res. 11: 23-32; Ibid. 63-71 ; Khalil & Grace (1992) J. Exp. Bot. 43: 1591-1602; Khan & Tsunoda (1970a) Tohuko J. Agric. Res. 21: 47-59; Khan & Tsunoda (1970a) Tohuko J. Agric. Res. 21: 60-72; Kim et al. (2003) Plant Soil 257: 443-449; Kim et al. (2003) Water Air Soil Poll. 145: 253-266; Kim et al. (2004) Env. Poll. 131: 287-294; Kim et al. (2004) Water Air Soil Poll. 145: 253-266; Kimura (1963) Jap. J. Bot. 18: 255-287; Kirkby & Mengel (1976) Plant Physiol. 42: 6-14; Kirschbaum et al. (1992) Aust. J. Plant Physiol. 19: 55-66; Kitajima (1994) Oecologia 98: 419-428; Klinge (1977; 1978) & Klinge et al (1975) From: Cannell (1982); Knorre (1977); Ignatenko et al. (1973ab); Bondarev (1989) Transactions of State Reserve “Stolby”. Issue 11. Krasnoyarsk, P. 91-100 & Ekologia (Rus. J. Ecol.) 3: 36-43 & Soils and vegetation of permafrost regions of UdSSR. Magadan: Far East Sci. Centre of AS USSR, P. 335-350 & Lesnaya taksatsiya i lesoustroistvo (Forest Inventory and Forest Planning). Krasnoyarsk: SibTI, P. 35-39; Koch et al. (1986) Physiol. Plant. 67: 477-484; Kochkin (1955) Ph. D. Thesis. Simferopol’, 1955; Kolari et al. (2004) Glob. Change Biol. 10: 1106-1119; Kolishchuk (1968) Lesovedeniye (Rus. For. Sci.) 4: 28-38; Kolli & Kahrik (1970a) Transactions of Estonian Agricultural Academy: Soil regimes and processes 65: 69-91; Koposov (1975) Izvestiya Sibirskogo Otdel. AS USSR. Biol. Series. 1: 30-35; Kornyak & Chertovskoi (1977) Materials of annual session on sci. results of 1976. Arkhangelsk: Forest and Forest Chemistry Industry Institute. P. 14-15; Kosarev & Uspenski (1983) Forest Inventory and Forest Planning. Krasnoyarsk, P. 117-123; Koshurnikova (2007) Ph. D. Thesis. Krasnoyarsk: V.N. Sukachev Forest Institute, 20 p.; Krauss & Allen (2003) Aq. Bot. 77: 311-324; Kravchenko (1963; 1964); Smirnov (1971a) Bot. Zhurnal (Rus. Bot. Journ.) 48: 566-570 & Lesnoi Zhurnal (Forest Journ.) 1: 45-47 & Organic mass of certain forest phytocoenoses at European part of USSR. Moscow: Nauka, 362 p.; Kuchko (1977) Biological and commercial productivity of forest phytocoenoses of Karelia. Petrozavodsk: Forest Institute of KF AS USSR, P. 64-71; Kulagina (1978) Pine forest productivity. Moscow: Nauka, P. 90-178; Kulagina (1986) Hydromorphic systems of forests and marshes. Krasnoyarsk: Sukachev Institute, P. 71-83; Kurashi et al. (1993) Ecol. Res. 8: 349-361; Kürschner et al. (1998) Ann. Bot. 81: 657-664; Kuyah et al (2012a,b) Agric. Ecosys. Environ 158: 216-224 & 225-234; Kuyah et al (2013) Biomass Bioenergy 55: 276-284; Kuzikov (1979a,b) Ph. D. Thesis. Krasnoyarsk: Siberian Technological Institute, 24 p. & Dissertation. Krasnoyarsk: Siberian Technological Institute, 266 p. (SibGTU library); Kwesiga & Grace (1986) Ann. Bot. 57: 283-290; Lai et al. (2004a) J. Fujian For. Sci. Technol. 31: 21-25, 30 (in Chinese); Lai et al. (2004b) For. Res. 17: 434-440 (in Chinese); Lakida et al. (2006) Biological productivity of oak forests of Podol’ye region. Kiev: Institute of Agricultural Economy, 196 p.; Lamade & Setiyo (1996) In: Proc. PORIM Intern. Palm Oil Congress, pp 427-435; Lamoreaux & Chaney (1977) J. Environ. Qual. 6: 201-205; Larigauderie et al. (1994) Plant Soil 165: 21-32; Larsen et al. (1976) Can. J. For. Res. 6: 187-194; Lashchinski (1981) Structure and dynamics of pine forests of the Lower Angara river’s region. Novosibirsk: Nauka, 272 p.; Laurence et al. (1994) J. Environ. Qual. 23: 412-417; Lazukova & Shuytsev (1980) Ecology and productivity of forests of Nechernozem’ye region (on Valdai Hills example); Moscow: Moscow State University, 1980. P. 50-56 ; Le Roux et al. (1996) Tree Physiol. 16: 497-502; LeBourgeois et al. (1998) Ann. Sci. For. 55: 278-299; Ledig et al. (1970) Bot. Gaz. 131: 349-359; Lee et al. (1996a) Ecology 77: 568-580; Lee et al. (1996b) J. Trop. For. Sci. 8: 520-531; Lee et al. (1997); Lee et al. (2000) Oecologia 110: 1-9; Am. J. Bot. 87: 447-455; Lee et al. (1999) J. Trop. For. Sci. 11: 132-147; Lehto & Grace (1994) New Phytol. 127: 455-463; Lemoine et al. (1986) Ann. Sci. For.

43: 67-84; Lemoine et al. (1988) Ann. Sci. For. 45: 95-116; Lewis et al. (1999) Planta 210: 104-114; Li & Chen (1996) J. Shandong For. Sci. Technol.: 44-45 (in Chinese); Li (1984) J. Guangxi Agr. Coll.: 88-100 (in Chinese); Li (1999) Plant Soil 214: 165-171; Li (2000) Sci. Silvae Sin. 36: 131-136 (in Chinese); Li (2002) J. Fujian For. Sci. Technol. 29: 6-8, 13 (in Chinese); Li (2007) Fujian Agr. Sci. Technol.: 76-77 (in Chinese); Li et al. (1981) Research of Forest Ecosystem 2: 34-50; Li et al. (1985) Acta Agr. Univ. Henan 19: 382-392 (in Chinese); Li et al. (1996) J. Cent. South For. Univ. 16: 47-51 (in Chinese); Li et al. (2006) J. Zhejiang For. Coll. 23: 362-366 (in Chinese); Li et al. (2007) J. Soil Water Conserv. 21: 59-63 (in Chinese); Liang et al. (1993) Trop. Subtrop. Soil Sci. 2: 218-226 (in Chinese); Liang et al. (1995) Guangxi Sci. 2: 54-58 (in Chinese); Liang et al. (2006) J. West China For. Sci. 35: 188-192 (in Chinese); Liang et al. (2008) J. Fujian For. Sci. Technol. 35: 14-18 (in Chinese); Liao (2006) Fujian J. Agr. Sci. 21: 411-414 (in Chinese); Liao et al. (1986) For. Sci. Technol.: 15-18 (in Chinese); Liao et al. (1988) J. Fujian Coll. For. 8: 252-257 (in Chinese); Liao et al. (1991) J. Fujian Coll. For. 11: 313-317 (in Chinese); Liao et al. (1992a) Sci. Silvae Sin. 28: 439-444 (in Chinese); Liao et al. (1992b) For. Sci. Technol.: 5-9 (in Chinese); Liao et al. (1994) For. Sci. Technol.: 15-17 (in Chinese); Liao et al. (1995) J. Nanjing For. Univ. 19: 59-66 (in Chinese); Lin & Yang (1996) J. Fujian For. Sci. Technol. 23: 21-24 (in Chinese); Lin (1991) J. Guangxi Agr. Coll. 10: 27-39 (in Chinese); Lin (2002) China For. Sci. Technol. 16: 21-23 (in Chinese); Lin (2004) Sci. Technol. Qinghai Agr. For.: 29-31 (in Chinese); Lin (2007) J. Fujian For. Sci. Technol. 34: 38-41 (in Chinese); Lin et al. (1993); Wu (1999) J. Fujian Coll. For. 13: 351-356 (in Chinese); J. Fujian Coll. For. 19: 265-269 (in Chinese); Lin et al. (1994) J. Fujian Coll. For. 14: 344-348 (in Chinese); Lin et al. (1996a) Sci. Silvae Sin. 32: 385-392 (in Chinese); Lin et al. (1996b) J. Xiamen Univ. (Nat. Sci.) 35: 269-275 (in Chinese); Lin et al. (1999) J. Fujian Coll. For. 19: 314-317 (in Chinese); Lin et al. (2003) China For. Sci. Technol. 17: 25-27 (in Chinese); Lindroth (1993) Ecology 74: 763-777; Liu & Stutzel (2004) Sci. Hortic. 102: 15-27; Liu & Stützel (2004) Sci. Hortic. 102: 15-27; Liu (1980) J. Henan Agr. Coll.: 21-31 (in Chinese); Liu (1984) For. Sci. Technol.: 10-13 (in Chinese); Liu (1987) J. Beijing For. Univ. 9: 1-10 (in Chinese); Liu (1991) J. Gansu For. Sci. Technol.: 13-18 (in Chinese); Liu (1995a) J. Fujian For. Sci. Technol. 22(S): 59-63 (in Chinese); Liu (1995a) J. Fujian For. Sci. Technol. 22: 59-63 (in Chinese); Liu (1995b) Guihaia 15: 327-334 (in Chinese); Liu (1998) J. Fujian For. Sci. Technol. 25: 33-37 (in Chinese); Liu (2006) Subtrop. Agr. Res. 2: 29-32 (in Chinese); Liu et al. (1990) J. Northeast For. Univ. 18: 40-45 (in Chinese); Liu et al. (1991) J. Northeast For. Univ. 19(S): 351-357 (in Chinese); Liu et al. (1993a) J. Fujian Coll. For. 13: 267-272 (in Chinese); Liu et al. (1993b) J. Fujian For. Sci. Technol. 20: 45-49 (in Chinese); Liu et al. (1995) For. Res. 8: 88-93 (in Chinese); Liu et al. (1997a) Sci. Silvae Sin. 33: 157-166 (in Chinese); Liu et al. (1997b) J. Nanjing For. Univ. 21: 6-10 (in Chinese); Liu et al. (1998) Chinese J. Appl. Ecol. 9: 569-574 (in Chinese); Liu et al. (2001) Sci. Silvae Sin. 37: 10-18 (in Chinese); Liu et al. (2002) For. Ecol. Man. 158: 223-235; Liu et al. (2003) Acta Ecol. Sin. 23: 1488-1497 (in Chinese); Liu et al. (2004) J. Fujian Coll. For. 24: 294-297 (in Chinese); Liu et al. (2004) Silva Fenn. 38: 235-242; Liu et al. (2007) J. Cent. South Univ. For. Technol. 27: 83-86 (in Chinese); Lodhiyal & Lodhiyal (1997) For. Ecol. Man. 98: 167-179; Logan & Krotkov (1968) Physiol. Plant. 22: 104-116; Lohmus & Oya (1983) Lesovedeniye (Rus. For. Sci.) 4: 56-62; Lopez-Hoffman et al. (2006) Biotropica 38: 606-616; Lopez-Hoffman et al. (2007) Oecologia 150: 545-556; Lopez-Iglesias et al. (2014) Acta Oecol. 56: 10-18; Lossaint & Rapp (1978) From: Cannell (1982); Loveys et al. (2002) Plant Cell Environ. 25: 975-988; Lu (2002) J. Hubei Inst. Natl. (Nat. Sci.) 20: 14-17 (in Chinese); Lu et al. (2000) J. Jiangsu For. Sci. Technol. 27: 12-15 (in Chinese); Lucas et al. (1993) Planta 190: 88-96; Luganskaya & Luganski (1970) Ural forests

and their management. Issue 4. Sverdlovsk: UralLOS, P. 69-90; Luganskaya (1970); Luganskaya & Luganski (1970) Dissertation. Sverdlovsk: Ural Forest Engineering Institute, 275 p. (USFEU library) & Ural forests and their management. Issue 4. Sverdlovsk: UralLOS, P. 69-90; Luk'yanets (1980) Ekologia (Rus. J. Ecol.) 2: 51-59; Luken et al. (1995) Can. J. Bot. 73: 1953-1961; Lukina & Nikonov,1991 Lesovedeniye (Rus. For. Sci.) 4: 37-45; Lukina (1996) Lesovedeniye (Rus. For. Sci.) 3: 28-37; Luo & Huang (1981) Shaanxi J. Agr. Sci.: 18-22 (in Chinese); Luo (1996); Ni et al. (2001) Dissertation. Committee of Synthesis Investigation of Natural Resources, Chinese Academy of Sciences. Beijing, 211 p. & Ann. For. Sci. 58: 351-384; Luo et al. (1998) Studies on Alpine Ecological Environment of Gongga Mountain (vol. 2)(eds Chen F.B., Luo J.): 10-15 (China Meteorological Press, 1998); Luo et al. (2004) Chinese J. Appl. Ecol. 15: 1329-1333 (in Chinese); Lusk et al. (1997) Oecologia 109: 49-58; Ma (1989) J. Beijing For. Univ. 11: 1-10 (in Chinese); Ma et al. (1989) J. Sichuan For. Sci. Technol. 10: 6-14 (in Chinese); Ma et al. (2002) J. For. Res.-China 13: 165-170; Ma et al. (2007) Chinese J. Plant Ecol. 31: 305-312 (in Chinese); Magambo & Cannell (1981) From: Cannell (1982); Maherali & DeLucia (2000) Am. J. Bot. 87: 243-249; Maillard et al. (2001) Tree Physiol. 21: 163-172; Maisner (1970) Floristic and geobotanical studies in Byelorussia. Minsk: Nauka I Tekhnika, P. 109-119; Malinovski & Kolishchuk (1971) Biological productivity of spruce forests. Tartu: Estonian Committee of IBP, P.119-124; Malkonen (1974) Communicationes Instituti Forestalis Fenniae. Helsinki. 84: 1-87; Malkonen (1977) Communicationes Instituti Forestalis Fenniae (Helsinki) 91: 1-35.; Manakov & Nikonov (1979; 1981) Botan. Zhurnal (Rus. Bot. J.) 64: 232-241 & Biological cycling of mineral elements and soil forming in spruce forests of Kraini Sever. Leningrad: Nauka. 196p.; Mandre et al. (1995) J. Am. Soc. Hortic. Sci. 120: 228-234; Manolova (1970) Gorskostopanska Nauka (Forest Sci.) 7: 3-17; Marchenko & Karlov (1961; 1962) Botan. Zhurnal (Rus. Bot. J.) 46: 1146-1152 & Pochvovedeniye (Rus. Soil Sci.) 7: 52-66; Marchenko & Karlov (1962) Pochvovedeniye (Rus. Soil Sci.) 7: 52-66; Markkola et al. (2002) Env. Poll. 116: 273-278; Marler & Clemente (2006) HortScience 41: 96-98; Maroco et al. (1999) Planta 210: 115-125; Maromin et al. (2008) Acta Bot. Bras. 22: 941-953; Martynenko (2004; 2005) Urgent problems of the forest complex. Issue 8. Bryansk: Engineering Academy, P. 97-99 & Ph.D. Thesis. Voronezh: Forest Engineering Academy, 18 p. ; Maruyama (1971; 1974) From: Cannell (1982); Masci et al. (1998); Persson et al. (2000); Scarascia-Mugnozza (2000) Borghetti M. (ed.). Proc. 1st Congr. of the Italian Sylviculture and Forest Ecology Society 1. Padova, Italy. P. 225-232 & E.-D. Schulze (ed.). Ecological Studies. Vol. 142. P. 14-46 & P. 49-62; Masci et al. (1998); Persson et al. (2000); Scarascia-Mugnozza (2000) Borghetti M. (ed.). Proc. 1st Congr. of the Italian Sylviculture and Forest Ecology Society 1. Padova, Italy. P. 225-232 & E.-D. Schulze (ed.). Ecological Studies. Vol. 142. P. 14-46 & P. 49-62; McDonald et al. (1986) Plant Cell Environ. 9: 433-438; McKee (1995) Am. J. Bot. 82: 299-307; Medina et al. (2009) Ann. Bot. 104: 671-680; Medvedeva (1974; 1977); Medvedeva et al. (1977) The ways of swamp studying and mastering on the North-West of European part of USSR. Leningrad: Nauka, P. 99-106 & Biological and commercial productivity of forest phytocoenoses of Karelia. Petrozavodsk: Karelian Branch of AS USSR, P. 71-75 & P. 44-58; Medvedeva (1974; 1978); Medvedeva & Egorova (1977); Medvedeva et al. (1977) The ways of swamp studying and mastering on the North-West of European part of USSR. Leningrad: Nauka, P. 99-106 & Dynamics of organic matter during to peat forming. Leningrad: Nauka, P. 8-32 & Biological and commercial productivity of forest phytocoenoses of Karelia. Petrozavodsk: Karelian Branch of AS USSR, P. 44-58 & Permanent studying of marsh-ridden forests and swamps in relation to their melioration. Petrozavodsk: Karelian Branch AS USSR. P. 123-147; Megonigal & Day (1992) Ecology 73:

1182-1193; Meijkamp et al. (2001) Plant Ecol. 154: 137-146; Menzel et al. (1994) J. Hortic. Sci. 69: 553-564; Merzlenko & Gurtsev (1982); Babich & Merzlenko (1998) Lesovedeniye (Rus. For. Sci.) 2: 85-88 & Biological productivity of forest plantations. Arkhangel'sk: State Technical University, 89 p.; Messier (1992) Can. J. Bot. 70: 2271-2276; Miao et al. (1992) Oecologia 90: 300-304; Miller (1963) From: Cannell (1982); Miller et al. (1976a,b) From: Cannell (1982); Miller et al. (1980) From: Cannell (1982); Miller et al. (1990) For. Sci. 36: 734-747; Minderman (1967) From: Cannell (1982); Mironenko (1998) Ph. D. Thesis. Bryansk: Engineering Techn. Academy. 20 p.; Miroshnichenko (1974) Rastitel'nye Resursy (Rus. Vegetation Resources) 10: 329-337; Miroshnichenko (1975) Botan. Zhurnal (Rus. Botan. J.) 60: 1776-1795; Miyazawa & Lechowicz (2004) Ann. Bot. 94: 635-644; Miyazawa et al. (2014) Agrofor. Syst. 88: 907-919; Molchanov (1964) Scientific basis for oak forest management on forest-steppe. Moscow: Nauka, 255 p.; Molchanov (1970) Interrelations of biogeocoenosis components in hardwood young forests. Moscow: Nauka, P. 32-77; Molchanov (1971) Productivity of organic mass in forests of different natural zones. Moscow: Nauka, 275 pp.; Molchanov (1974) Productivity of organic and biological forest mass. Moscow: Nauka, P. 141-161; Molchanov (1977) Forestry studies at the southern taiga subzone. Moscow: Nauka, P. 51-60 ; Molchanov (1977); Molchanov et al. (1982) Forestry studies at the southern taiga subzone. Moscow: Nauka, P. 51-60 & Biological productivity of forests at Povolgie region. Moscow: Nauka, P. 147-163; Montero et al. (2005) In: Produccion de biomasa y fijacion de CO2 por les Bosques Espanoles; Morikawa et al. (1976) J. Jap. For. Soc. 58: 174-178; Morison & Gifford (1984) Plant Physiol. 75: 275-277; Morozova (1978) Soils of pine forests on Karelia. Petrozavodsk: Forest Institute of Karel. Branch of AS USSR, P. 85-112; Mortensen (1994) Acta Agric. Scand. 44: 164-169; Mortensen (1995) Environ. Poll. 87: 337-343; Moskalyuk (1979; 1980; 1984) Biological cycling in forest-tundra of the southern Magadan region. Vladivostok, P. 16-27 & Lesovedeniye (Rus. For. Sci.). 2: 32-39 & Ph. D. Thesis. Krasnoyarsk: V.N. Sukachev Forest Institute. 27 pp.; Mousseau & Saugier (1992) J. Exp. Bot. 43: 1121-1130; Mu et al. (1995ab) J. Northeast For. Univ. 23: 95-102 (in Chinese); Bull. Bot. Res. 15: 551-557 (in Chinese); Mueller & Day (2005) Int. J. Biometeorol. 49: 244-255; Mund et al. (2002) For. Ecol. Manage. 171: 275-296; Myakushko (1972) Ukrainian Bot. J. 29: 328-339; Myakushko (1972; 1978) Ukrainian Bot. J. 29: 328-339 & Scots pine forests of plain part of the Ukrainian republic: geobotanical characteristics and primary biological productivity. Kiev: Naukova Dumka, 256 p.; Nagimov et al. (2007) Coniferous species of boreal zone 24: 427-430; Nakaji & Izuta (2001) Water Air Soil Poll. 130: 971-976; Nativ et al. (1999) Aust. J. Bot. 47: 577-586; Neirynck et al. (1998) Ann. For. Sci. 55: 389-405; Nemich (1991a,b) Use and regeneration of resources of Angara-Yenisei region. Krasnoyarsk: Sib. Technol. Institute, P. 141-145 & P. 89-93; Neshataev et al. (1974a,b); Goryshina (1974) Biological productivity and its defermining factors on a forest-steppe oak forest. Leningrad: State University, P. 119-152 & 7-40 & Ekologia (Rus. J. Ecol.) 3: 5-10; Neufeld (1983) Bull. Torrey Bot. Club 110: 43-54; Newsome et al. (1982) Can. J. Bot. 60: 1688-1695; Ngugi et al. (2003) New For. 26: 187-200; Nicotra et al. (1997) Am. J. Bot. 84: 1542-1552; Nie (1993) For. Res. 6: 643-649 (in Chinese); Nie et al. (1997) Sci. Silvae Sin. 33: 394-402 (in Chinese); Nieves et al. (2011) Tree Physiol. 31: 92-101; Nihlgard & Lindgren (1977) Oikos 28: 95-104; Nihlgard (1972) Oikos 23: 69-81; Nikolopoulos et al. (1995) Aust. J. Plant Physiol. 22: 737-745; Nikonov & Tsvetkov (1984) Lesovedeniye (Rus. For. Sci.) 3: 37-41; Nikula et al. (2009) Boreal Environ. Res. 14: 29-47; Nilsen & Semones (1997) Int. J. Plant Sci. 158: 827-834; Nogues et al. (1998) Plant Physiol. 117: 173-181; Noh et al. (2007) J. Plant Biol. 50: 461-466; Norby & O'Neill (1991) New Phytol. 117: 515-528; Norby et al. (1986) Plant Physiol. 82: 83-89; Nordborg et al. (2003)

For. Ecol. Manage. 180: 571-582; Nurpeisov (1986) Ph. D. Thesis. Alma-Ata: Kazakh Agricultural Institute. 21 p.; Nykvist (1971) Systems analysis in northern coniferous forests - IBP workshop. Bulletins from the Ecological Research Committee. Vol. 14: 166-178; Oberbauer et al. (1985) Physiol. Plant. 65: 352-356; Oberbauer et al. (1986) Can. J. Bot. 64: 2993-2998; Odinak & Borsuk (1977; 1983); Odinak (1992) Ukrainian Bot. J. 34: 408-414 & Biogeocoenotic cover of Beskidy Mountain and its dynamics trends. Kiev: Naukova Dumka, P. 20-103 & Ph. D Thesis. Dnepropetrovsk: State University, 63 p. ; Odinak & Borsuk (1983) Biogeocoenotic cover of Beskidy Mountain and its dynamics trends. Kiev: Naukova Dumka, P. 20-103; Odinak et al. (1986) Forestry, Pulp and Wood Industry 17: 13-15; Odinak et al. (1987) Lesnoi Zhurnal (For. J.) 4: 23-26; Ogino (1977) From: Cannell (1982); Oksanen (2001) Water Air Soil Poll. 130: 947-952; Oleksyn et al. (1992) New Phytol. 120: 561-574; Oleksyn et al. (1998) Funct. Ecol. 12: 573-590; Oleksyn et al. (1999) Scand. J. For. Res. 14: 7-17; Olesniewicz & Thomas (1999) New Phytol. 142: 133-140; Olszyk et al. (2003) Can. J. For. Res. 33: 269-278; Olufolaji & Tayo (1980) Sci. Hortic. 13: 181-189; O'Neill et al. (1987a) Plant Soil 104: 3-11; O'Neill et al. (1987b) Can. J. For. Res. 17: 878-883; Orians et al. (1999) Can. J. Bot. 77: 514-522; Os'kina (1975; 1982) Scientific Transactions of Moscow Forest Engineering Institute 72: 43-47 & Ph. D. Thesis. Moscow: Forest Engineering Institute, 16 p.; Oshima (1961ab) From: Cannell (1982); Osorio et al. (1998) Tree Physiol. 18: 363-373; Osunkoya & Ash (1991) Aust. J. Bot. 39: 591-605; Osunkoya et al. (1994) J. Ecol. 82: 149-163; Ottorini & Le Goff (1998); Le Goff & Ottorini (2001) Biomasses aériennes et racinaires et accroissements annuels en biomasse dans le dispositif écophysiologique de la forêt de Hesse. Report. INRA, Nancy, 29 p. & Ann. For. Sci. 58: 1-13; Ouvrier (1989) Oleagineux 39: 73-80; Ovalle et al. (1996) Plant Soil 179: 131-140; Ovington & Madgwick (1959b) From: Cannell (1982); Ovington & Madgwick (1959b) Plant Soil 10: 389-400; Ovington & Olson (1970) In: A tropical rainforest; H53-H77; Ovington (1957) Ann. Bot. 21: 287-314;

P-T

Paakkonen & Holopainen (1995) New Phytol. 129: 595-603; Paavilainen (1980) Communicationes Instituti Forestalis Fenniae (Helsinki) 98: 230-241; Pajtik et al. (2008) For. Ecol. Man. 256: 1096-1103; Pajtik et al. (2008) For. Ecol. Manage. 256: 1096-1103; Pan (2005a) China For. Sci. Technol. 19: 16-18 (in Chinese); Pan (2005b) Jiangxi For. Sci. Technol.: 5-6, 22 (in Chinese); Pan et al. (1978) Hubei For. Sci. Technol.: 1-12 (in Chinese); Pan et al. (1983) J. Cent. South For. Inst. 3: 1-17 (in Chinese); Pan et al. (2008) J. Fujian For. Sci. Technol. 35: 6-10 (in Chinese); Park et al. (2005) Ecol. Res. 20: 227-231; Park et al. (2005) Kor. J. Env. Ecol. 19: 299-304; Parshevnikov (1962); Rodin & Bazilevich (1965) Transactions of V.N. Sukachev Forest and Wood Institute 52: 196-209 & Problems of modern botany. Moscow-Leningrad: Nauka, P. 236-243; Pasternak & Chernyavskii (1977) Forest Management and Forest Agromelioration. Forestry and Forest Management 49: 27-33; Patel & Pandey (2007) J. Arid Environ. 70: 174-182; Pautova (1976a); Molozhnikov & Pautova (1976) Transactions of Limnological Inst. 9: 144-360 & Pinus pumila on mountain landscapes of the Northern Pribaikal'ye. Moscow, Nauka, 203 p.; Pearson et al. (1984) Can. J. For. Res. 14: 259-265; Peet (1986) Plant Physiol. 80: 59-62; Pehl et al. (1984) For. Ecol. Man. 9: 155-160; Pellinen (1986) Dissertation Georg-August-Universität Göttingen. Göttingen, 145 s.; Pena-Roja et al. (2005) Funct. Plant Biol. 32: 117-130; Tree Physiol. 24: 813-822; Peng (2003) J. Fujian Coll. For. 23: 128-131 (in Chinese); Peng (2008) J. Fujian For. Sci. Technol. 35: 15-18, 23 (in Chinese); Peng et al. (1993) Plant Physiol. 101: 1063-1071; Perala & Alban (1982) Plant Soil 64: 177-192;

Perekhod (1974) Forest Science and Praxis 24: 31-33; Peri et al. (2008) For. Ecol. Man. 255: 2502-2511; Persson et al. (2000); Scarascia-Mugnozza (2000) In: Schulze (ed.). Carbon and nutrient cycling in European forest ecosystems. (Ecological Studies. Vol. 142). P. 14-46. & 49-62; Peterken & Newbould (1966) J. Ecol. 54: 143-150; Phillips & Fahey (2005) Glob. Change Biol. 11: 983-995; Picon et al. (1996) Ann. Sci. For. 53: 431-446; Picon et al. (1997) J. Exp. Bot. 48: 1547-1556; Pieters (1989) Ann. Sci. For. 46: 673s-679s; Pilon-Smits et al. (1995) Plant Physiol. 107: 125-130; Pis'merov et al. (1979) Lesovedeniye (Rus. For. Sci. No. 1. P. 68-72; Pisarenko et al. (1979) Lesovedeniye (Rus. For. Sci. No. 1. P. 68-72; Pleshikov et al. (2002) Forest ecosystems of the Yenisey Meridian. Novosibirsk: Publishing House of SB RAS, P. 73-83; Polikarpov (1962) Pine young forest dynamics on clear cuttings. Moscow: Academy of Sci. USSR, 171 p.; Pollard & Wareing (1968) Ann. Bot. 32: 573-591; Polley et al. (2002) Tree Physiol. 22: 383-391; Polyakov (1973) Creating high productive forests on drained lands. Moscow: Lesnaya Promyshlennost¢, 119 p.; Poorter (1991) Thesis, Utrecht; Poorter (1993) Vegetatio 104/105: 77-97; Poorter (1999) Funct. Ecol. 13: 396-410; Poorter et al. (1995) Plant Soil 171: 217-227; Poot & Lambers (2003) Plant Soil 253: 57-70; Portsmuth & Niinemets (2007) Funct. Ecol. 21: 61-77; Possen et al. (2011) For. Ecol. Manage. 262: 1387-1399; Pozdnyakov (1963; 1967; 1975a,b) Gidrology and climate conditions of larch forests in Central Jakutia. Moscow: Academy of Sciences of the USSR, 146 pp. & Lesovedeniye (Rus. For. Sci.) 6: 36-42 & Biospheric resources: Synthesis of the Soviet studies for the International Biological Programme. Vol. 1. Leningrad: Nauka, P. 43-55 & Dahurian larch. Moscow: Nauka, 312 pp.; Pozdnyakov (1967; 1975a) Lesovedeniye (Rus. For. Sci.) 6: 36-42 & Biospheric resources: Synthesis of the Soviet studies for the International Biological Programme. Vol. 1. Leningrad: Nauka, P. 43-55; Pozdnyakov (1975a); Gorbatenko (1970) Biospheric resources: Synthesis of the Soviet studies for the International Biological Programme. Vol. 1. Leningrad: Nauka, P. 43-55 & Ph. D. Thesis. Krasnoyarsk: Sukachev Forest Institute, 29 p.; Pozdnyakov et al. (1969); Protopopov (1971) Biological productivity of forests of the Middle Siberia and Jakutia. Krasnoyarsk: Book Publ. House, 120 p & Lesovedeniye (Rus. For. Sci.) No. 1. P. 32-36; Pregitzer et al. (1995) New Phytol. 129: 579-585; Prior et al. (1997) Tree Physiol. 17: 397-405; Prokhorova (1972) Studies of Siberian forest nature. Krasnoyarsk: V.N. Sukachev Forest Institute, P. 30-36; Protopopov (1971); Pozdnyakov (1975a) Biological productivity and cycling of chemical elements in plant communities. Leningrad: Nauka, P. 59-65 & Biospheric resources: Synthesis of the Soviet studies for the International Biological Programme. Vol. 1. Leningrad: Nauka, P. 43-55.; Prozorovski & Samoilova (1972) Soils and productivity of plant communities. Issue 1. Moscow: State University, P. 178-198; Pulkkinen & Poykko (1990) Tree Physiol. 6: 381-390; Puri et al. (1994) For. Ecol. Man. 65: 135-147; Pyavchenko (1967) Lesovedeniye (Rus. For. Sci.) 3: 32-43; Qi et al. (2007) Chinese J. Ecol. 26: 1697-1702 (in Chinese); Qian & Ye (1992) J. Nanjing For. Univ. 16: 19-24 (in Chinese); Qin et al. (2007) J. Northeast For. Univ. 35: 22-24 (in Chinese); Qin et al. (2008) J. Northwest For. Univ. 23: 17-20, 27 (in Chinese); Quero et al. (2006; 2008) New Phytol. 170: 819-834; Quero et al. (2007) Am. J. Bot. 94: 1795-1803; Radoglou & Jarvis (1990) Ann. Bot. 65: 617-627; Rakhteyenko (1952) Root systems of trees and shurbs. Moscow, Leningrad: Goslesbumizdat, 107 p.; Ramam (1981) From: Cannell (1982); Ramoliya & Pandey (2002) J. Arid Environ. 51: 121-132; Ramoliya & Pandey (2003) For. Ecol. Manage. 176: 185-194; Ramos et al. (2004) Acta Bot. Bras. 18: 351-358; Ran et al. (1994) Agron. J. 86: 530-534; Ranger & Gelhaye (2001) Ann. For. Sci. 58: 423-430.; Ranger et al. (1992) Ann. Sci. For. 49: 651-668; Rawat & Singh (1988) Ann. Bot. 62: 397-411; Rawat & Singh (1988) Ann. Bot. 62: 397-411; Redondo-Gomez et al. (2007) Ann. Bot.

100: 555-563; Rees & Chapas (1963) Ann. Bot. 27: 607-614; Rees & Tinker (1963) Plant Soil 19: 19-32; Reich et al. (1993) Trees 7: 67-77; Reich et al. (1998c) Funct. Ecol. 12: 327-338; Reich et al. (2003) New Phytol. 157: 617-631; Remezov & Bykova (1953) Pochvovedeniye (Rus. Soil Sci.) 8: 28-41; Remezov (1961) Transactions of the Voronezh reserve 13: 9-53; Remezov et al. (1959) Uptake and cycling of nitrogen and ash elements in forests of European part of USSR. Moscow: State University, 284 p. ; Remezov et al. (1959); Rodin & Bazilevich (1967) Uptake and cycling of nitrogen and ash elements in forests of European part of USSR. Moscow: State University, 284 p. & Production and mineral cycling in terrestrial vegetation. Edinburg: Oliver and Boyd, 1967; Ren et al. (2000) Acta Phytoecol. Sin. 24: 18-21 (in Chinese); Rencz & Auclair (1978) Can. J. For. Res. 14: 259-265; Reubens et al. (2009) Trees 23: 1213-1228; Rey & Jarvis (1997) Ann. Bot. 80: 809-816; Richards & Rowe (1977) Ann. Bot. 41: 729-740; Ripullone et al. (2004) Tree Physiol. 24: 671-679; Robakowski et al. (2003) Trees 17: 431-441; Rochefort & Bazzaz (1992) Can. J. For. Res. 22: 1583-1587; Roden et al. (1997) Ecology 78: 385-393; Roden et al. (1999) Aust. J. Plant Physiol. 26: 37-46; Rodin & Bazilevich (1967); Bazilevich (1965) Production and mineral cycling in terrestrial vegetation. Edinburg: Oliver and Boyd & Geochemistry of soda soil salting. Moscow: Nauka, 351p. ; Rodionov et al. (1974; 1975) Biological productivity of vegetation of Kazakhstan. Alma-Ata: Nauka, P. 234- 274 & Lesovedeniye (Rus. For. Sci.) No. 1. P. 20-27; Rodriguez et al. (2008) Plant Ecol. 195: 273-285; Rokjanis (1978; 1981) Transactions of Latvian Agricult. Academy. Issue 143 P. 43-56 & cology and forest preservation. Transactions of sci. works. Issue 6. Leningrad: Forest Engineering Academy. P. 4-12; Rothstein & Cregg (2005) For. Ecol. Manage. 219: 69-80; Rouhier et al. (1994) Plant Soil 162: 281-292; Rozanova (1960) Transactions of Forestry Laboratory of Academy of Sciences of USSR 1: 5-60.; Rozanova (1960); Utkin (1970) Transactions of Forestry Laboratory of Academy of Sciences of USSR 1: 5-60 & Lesovedeniye (Rus. For. Sci.) 3: 58-89; Rozhdestvenski (1979; 1982); Kashlev (1968) Lesovedeniye (Rus. For. Sci.) 4: 30-37 & Biological productivity of Povolgie forests. Moscow: Nauka, P. 99-109 & Reports of Timiryazev Agricult. Acad. 139: 249-252; Ru (1996) Henan Sci. 14(S): 64-65, 73 (in Chinese); Rubtsov & Rubtsov (1975) Lesovedeniye (Rus. For. Sci.) No. 1. P. 28-36 ; Rudneva et al. (1966) Pochvovedeniye (Rus. Soil Sci.) 3: 14-26; Ruiz et al. (1997) Tree Physiol. 17: 141-150; Ruiz-Robleto & Villar (2005) Plant Biol. 7: 484-494; Runion et al. (1999) Tree Physiol. 19: 329-335; Rusanova & Sloboda (1974; 1977) Botan. Zhurn. (Rus. Bot. J.) 59: 1827-1833 & Lesovedeniye (Rus. For. Sci.) №. 2. P. 13-19; Ryser & Lambers (1995) Plant Soil 170: 251-265; Sack & Grubb (2002) Oecologia 131: 175-185; Sack (2004) Oikos 107: 110-127; Safronova & Nipa (1979) Larch: Inter-Institutional Collection of Scientific works. Krasnoyarsk: State University, P. 46-52; Saitio et al. (1966; 1967) From: Cannell (1982); Saito & Shidei (1973); Satoo (1979d) J. Jap. For. Soc. 55: 52-62 & J. Jap. For. Soc. 61: 83-87; Saito (1977) Primary productivity in Japanese forest. JIBP Synthesis. University of Tokyo Press. Vol. 16: 252-268; Saito et al. (1968) From: Cannell (1982); Saito et al. (1970) Bull. Kyoto Univ. For. 41: 80-95; Saito et al. (1972) Bull. Kyoto Univ. For. 44: 121-140; Salmina (1973) Ph. D. Thesis. Moscow: Moscow State University, 23 p.; Samuelson & Edwards (1993) New Phytol. 125: 373-379; Samuelson & Seiler (1992) Env. Exp. Bot. 32: 352-356; Samuelson (1994) New Phytol. 128: 235-241; Samuelson et al. (1996) Environ. Poll. 91: 317-323; Satoo (1951) From: Cannell (1982); Satoo (1970) J. Jap. For. Soc. 52: 154-158; Satoo (1974) Bull. Tokyo Univ. For. 66: 153-164; Satoo (1974b) From: Cannell (1982); Schierenbeck et al. (1994) Ecology 7: 1661-1672; Schippers & Olff (2000) Plant Ecol. 149: 219-231; Schortemeyer et al. (1999) Aust. J. Plant Physiol. 26: 737-747; Schwilk & Ackerly (2005) Am. J. Bot. 92: 404-410; Searles et al. (1995)

Am. J. Bot. 82: 445-453; Sebastiani et al. (2004) Env. Exp. Bot. 52: 79-88; Semechkina (1978) Phytomass structure in Scots pine. Novosibirsk: Nauka, 1978. 165 p.; Sena Gomes & Kozlowski (1980) Plant Physiol. 66: 267-271; Shan et al. (1995) Water Air Soil Poll. 85: 1399-1404; Shan et al. (1997) Water Air Soil Poll. 97: 355-366; Shanklin & Kozlowski (1985) Env. Poll. 38: 199-212; Sharew et al. (1996) Tree Physiol. 16: 617-626; Shcherbakov & Zaitseva (1971) Forest plant resources of the Southern Karelia. Petrozavodsk: Karelia Publ. House. P 22-40; Shen et al. (2005) J. Hubei Inst. Natl. (Nat. Sci.) 23: 289-292 (in Chinese); Sheng & Fan (2005) Long-Term Productivity of Chinese Fir Plantations (eds Sheng W.T., Fan S.H.)(Science Press, 2005); Sheng & Xue (1992) Sci. Silvae Sin. 28: 397-404 (in Chinese); Sheriff (1992) Aust. J. Plant Physiol. 19: 637-652; Shi (1989) Acta Agr. Univ. Jiangxi 11: 32-45 (in Chinese); Shi et al. (1996) Acta Phytoecol. Sin. 20: 524-533 (in Chinese); Shipley (2002) Funct. Ecol. 16: 682-689; Shorrocks (1965) From: Cannell (1982); Shugaley (1998) Lesovedeniye (Rus. For. Sci.) No. 3. P. 3-11; Sidorovich & Bus'ko (1982); Bus'ko (1986) Proc. of Byelorus. Acad. Sci. Ser. Biol. 4: 98-100 & Ph.D. Thesis. Dnepropetrovsk: Dnepropetrovsk University, 24 p.; Sidorovich & Bus'ko (1982); Bus'ko (1986) Proc. of Byelorus. Acad. Sci. Ser. Biol. 4: 98-100 & Ph.D. Thesis. Dnepropetrovsk: Dnepropetrovsk University, 24 p.; Sidorovich et al. (1985) Functioning of forest phytocoenoses under anthropogenous pressing. Minsk: Nauka i Tekhnika, 206 p.; Sidorovich et al. (1985); Bus'ko (1986) Functioning of forest phytocoenoses under anthropogenous pressing. Minsk: Nauka i Tekhnika, 206 p. & Ph.D. Thesis. Dnepropetrovsk: Dnepropetrovsk University, 24 p.; Sigurdsson et al. (2001) Tree Physiol. 21: 941-950; Simon et al. (2014) Tree Physiol. 34: 49-60; Singh & Misra (1979) Final Technical Report. Indian MAB Project. Varanasi: Banaras Hindu University, India.; Singh & Sharma (1976) From: Cannell (1982); Singh et al. (1980) From: Cannell (1982); Singh et al. (1994) Ecol. Monogr. 64: 401-421; Singh et al. (2004) J. Trop. For. Sci. 16: 384-395; Singh et al. (2009) Sci. Hortic. 120: 173-180; Singh(1975); Misra (1972); Sharma et al. (1981) From: Cannell (1982); Sionit et al. (1985) Can. J. For. Res. 15: 468-471; Sirotkin & Gruk (1980) Forestry and Forest Management. Issue 15. Minsk, P. 35-39; Skovsgaard et al. (2006) Scan. J. For. Res. 21: 470-488; Slemnev (1969) Ph. D. Thesis. Leningrad: Forest Engin. Acad., 20 p.; Smirnov (1971a,b); Semenova (1975) Organic mass of certain forest phytocoenoses at European part of USSR. Moscow: Nauka, 362 p. & Ph. D. Thesis. Minsk: Experimental Botany Institute, 45 p. & Influence of final clear cutting on soils and matter cycling in forest ecosystem. Moscow: Lesnaya Promyshlennost, 183 p.; Smirnov (1971a,b) Organic mass of certain forest phytocoenoses at European part of USSR. Moscow: Nauka, 362 p. & Ph. D. Thesis. Minsk: Experimental Botany Institute, 45 p. ; Smirnov (1971a; 1974) Organic mass of certain forest phytocoenoses at European part of USSR. Moscow: Nauka, 362 p. & Modern problems of forestry and forest biogeocoenology. Moscow: Nauka, P. 137-156; Smirnova & Gorodentseva (1958) Bull. of MOIP. Biol. Division 63: 135-145; Smirnova (1951); Remezov et al. (1959) Vestnik Moscow State Univ. No. 10. P. 103-122; Smith & Lee (1999) Bull. Mar. Sci. 65: 795-806; Son et al. (2004) Ecol. Res. 19: 21-28; Son et al. (2007) Euras. J. For. Res. 10: 41-50; Song et al. (1997) J. Beijing For. Univ. 19: 99-103 (in Chinese); Squire et al. (1987) Aust. For. Res. 17: 99-111; Stakanov (1990) Lesovedeniye (Rus. For. Sci.) No. 4. P. 25-33; Stienstra (1986) Plant Cell Environ. 9: 307-313; Stoneman & Dell (1993) Tree Physiol. 13: 239-252; Su (1988) Shaanxi For. Sci. Technol.: 28-30 (in Chinese); Su (1995) J. Sichuan For. Sci. Technol. 16: 36-42 (in Chinese); Su (2000) J. Fujian Coll. For. 20: 35-41 (in Chinese); Su et al. (2000) J. Sichuan For. Sci. Technol. 21: 31-35 (in Chinese); Su et al. (2003) J. Nanjing For. Univ. (Nat. Sci.) 27: 107-109 (in Chinese); Sullivan & Teramura (1988) Am. J. Bot. 75: 225-230; Sun (1981) For. Sci. Technol.: 11-12 (in Chinese); Sun et al. (1992)

Proceedings of Forest Ecosystems on Xiashu Ecological Station (eds Jiang Z.L.): 16-22 (China Forestry Publishing House, 1992) (in Chinese); Sun et al. (2006) Syst. Sci. Compr. Stud. Agr. 22: 46-49 (in Chinese); Susiluoto & Berninger (2007) Silva Fennica 41: 221-233; Sutinen et al. (1997) Ann. Bot. Fenn. 34: 265-273; Swamy et al. (2004) Biom. Bioener. 26: 305-317; Tadaki & Kawasaki (1966) From: Cannell (1982); Tadaki et al. (1962); Tadaki (1965b; 1968b) J. Jap. For. Soc. 44: 350-359 & Jap. J. Ecol. 15: 142-147 & J. Jap. For. Soc. 50: 60-65; Tadaki et al. (1963); Tadaki (1965a; 1968a) J. Jap. For. Soc. 45: 293-301 & J. Jap. For. Soc. 47: 384-391 & Bull. Government. Forest Exper. Station Tokyo. 216: 99-115; Tadaki et al. (1964; 1965) J. Jap. For. Soc. 46: 246-253 & Bull. Gov. Forest Exper. Station Tokyo 173: 45-66; Tadaki et al. (1965) From: Cannell (1982); Tadaki et al. (1966) J. Jap. For. Soc. 48: 387-393; Tadaki et al. (1967b); Tadaki & Kawasaki (1966) Bull. Gov. For. Exper. Station Tokyo 199: 47-65 & J. Jap. For. Soc. 48: 55-61; Tadaki et al. (1967a; 1970) J. Japan. For. Sec. 49: 421-428 & Bull. Gov. For. Expt Station Tokyo 229: 1-20; Tadaki et al. (1968) From: Cannell (1982); Tadaki et al. (1969) J. Jap. For. Soc. 51: 331-339.; Takeuchi et al. (1975) Bull. Gov. For. Exper. Station Tokyo 272: 141-155; Tamai & Shidei (1971) From: Cannell (1982); Tan & Song (1984) Eucalypt Sci. Technol.: 1-11 (in Chinese); Tanabe et al. (2003) J. For. Res. 8: 247-252; Tang & Kozlowski (1982a) Plant Soil 66: 243-255; Tang & Kozlowski (1982b) Can. J. For. Res. 12: 196-202; Tang & Kozlowski (1983a) Physiol. Plant. 59: 218-222; Tang & Kozlowski (1983b) Can. J. For. Res. 13: 633-639; Tang & Xu (1993) Sichuan For. Explor. Design : 27-32 (in Chinese); Tang & Xu (1997) Sichuan For. Explor. Design : 21-26 (in Chinese); Ter Steege et al. (1994) Oecologia 100: 35-367; Terekhov & Usoltsev (2005) Ecological problems of the northern territory. Issue 8. Arkhangelsk: State Technical University. P. 107-108; Teskey & Will (1999) Tree Physiol. 19: 519-525; Thomas & Strain (1991) Plant Physiol. 96: 627-634; Thomas et al. (1991) Oecologia 88: 415-421; Thompson et al. (1992) Aust. J. Plant Physiol. 19: 1-18; Aust. J. Plant Physiol. 19:19-42; Tian et al. (1990) J. Cent. South For. Coll. 10: 121-128 (in Chinese); Tian et al. (1997) J. Beijing For. Univ. 19: 104-107 (in Chinese); Tian et al. (2002) Sci. Silvae Sin. 38: 14-18 (in Chinese); Tinoco-Ojanguren & Pearcy (1995) Funct. Ecol. 9: 222-230; Tirado & Slater (2010) Arboric. Urban For. 36: 164-170; Tissue et al. (1996) Tree Physiol. 16: 49-59; Tjoelker & Luxmoore (1991) New Phytol. 119: 69-81; Tjoelker et al. (1993) New Phytol. 124: 627-636; Tognoni et al. (1967) Planta 72: 43-52; Tokmurzin & Nurpeisov (1976); Nurpeisov (1976) Sci. Transactions of Kazakh Agric. Inst. 19: 127-136 & Sci. Transactions Kazakh Agric. Inst. 19: 89-93; Tolley & Strain (1984) Can. J. For. Res. 14: 343-350; Tomlinson et al. (2012, 2013) J. Ecol. 100: 1113-1121; Ann. Bot. 112: 575-587; Tomlinson et al. (2013) Ann. Bot. 112: 575-587; Tonkonogov & Dorokhova (1968) Bull. Dokuchaev Soil Inst. #2. Moscow, P. 11-24; Torlopova (2001) Bio-production process at the northern forest ecosystems. St.-Petersburg: Nauka, P. 68-72; Torlopova (2001) Bio-production process at the northern forest ecosystems. St.-Petersburg: Nauka, P. 68-72; Trione & Passera (1993) J. Arid Environ. 25: 331-341; Tschaplinski & Blake (1985) Physiol. Plant. 64: 167-176; Tschaplinski & Blake (1989) Can. J. Bot. 67: 1689-1697; Tschaplinski et al. (1993) Tree Physiol. 13: 283-296; Tsykunov (1972a,b); Tsykunov et al. (1984; 1986) Forestry and Forest Management. Issue 6. Minsk, P. 39-43 & : Ph. D. Thesis. Minsk: Byelorus. Techn. Institute, 22 p. & Lesnoi Zhurnal (For. J.) 6: 20-23 & Botany: Studies. Issue 27. Minsk: Nauka i Tekhnika, P. 226-227; Turnbull (1991) & Thesis (1992) Oecologia 87: 110-117 & Thesis; Turner et al. (1976) J. Ecol. 64: 965-974; Tyree et al. (2009) For. Ecol. Manage. 257: 1847-1858;

U-Z

Uenaka et al. (1972); Kabaya et al. (1964) Rep. Kyoto Univ. For. 10: 53-59 & Bull. Marine Lab. Chiba Univ. 6: 1-26; Uri et al. (2009) Forestry 82: 61-64; Ushio et al. (2008) Soil Sci. Plant Nutr. 54: 253-258; Usoltsev & Shcherba (1998) Phytomass structure in Pinus sibirica plantations. Krasnoyarsk: Sib. Technol. University, 134 p.; Usoltsev (1998) Growth and structure of forest biomass. Novosibirsk: Nauka, 253 p.; Usoltsev (2001b); Pryazhnikov & Pertsev (1971) Forest Inventory and Forest Planning. Krasnoyarsk: Sib. Technological University, P. 44-46 & Proc. For. Manage. on Western Siberia. Issue 9. P. 122-129; Usoltsev et al. (1999b); Gabeyev (1968; 1969) Forest Inventory and Forest Planning. Krasnoyarsk: Sib. Tech. Univ. P. 16-24 & Izv. Sib. Otdel. AS. USSR. Biol. and Medicine Series. 15: 98-106 & Lesovedeniye (Rus. For. Sci.) No. 3. P. 75-78; Usoltsev et al. (2002b) Ecology: science, education, training. Issue 3. Bryansk: Engineering and Technical Academy, P. 48-51; Usoltsev et al. (2004b) Modern problems of forest complex: Collection of scientific works. Issue 9. Bryansk: Engineering and Technical Academy, P. 53-57; Usoltsev et al. (2004a) Ural forests and its management. Issue 24. Ekaterinburg: State Forest Engineering University. P. 144-147; Usoltsev et al. (2005b) Modern problems of forest complex: Collection of scientific works. Issue 10. Bryansk: Engineering and Technical Academy, P. 67-69; Uspenski (1983) Lesovedeniye (Rus. For. Sci.) No. 6. P. 50-53; Uspenski (1987) Complex and rational use of forest resources. Voronezh: Forest Engineering Institute. P. 47-59; Utenkova & Flyagina (1983) Lesovedeniye (Rus. For. Sci.) No. 5. P. 57-63; Utenkova (1974) Byelovezhskaya Pushcha: Studyings. Issue 8. Minsk: Uradzhai, P. 73-98; Utenkova et al. (1971); Utenkova (1974) Byelovezhskaya Pushcha: Studies. Issue 5. P. 75-96 & Byelovezhskaya Pushcha: Studyings. Issue 8. Minsk: Uradzhai, P. 73-98; Utenkova et al. (1973) Byelovezhskaya Pushcha: Studies. Issue 7. Minsk: Uradzhai, P. 39-51; Utkin & Dylis (1966) Bull. MOIP. Biol. Division. 71: 79-91; Utkin et al. (1984) Lesovedenye (Rus. For. Sci.) No. 3. P. 28-36; Utkin et al. (1997) Lesovedeniye (Rus. For. Sci.) No. 5. P. 51-65; Vakurov & Polyakova (1982a) Chemical matter cycling in forests. Moscow: Nauka, P. 20-43; Vakurov & Polyakova (1982b) Chemical matter cycling in forests. Moscow: Nauka, P. 44-54; Vakurov (1973; 1974b) Productivity of forest organic mass at different natural zones. Moscow: Nauka, P. 7-27 & Productivity of organic and biological mass of forests. Moscow: Nauka, P. 11-15; Vakurov (1974a) Productivity of organic and biological forest mass. Moscow: Nauka, P. 7-10; Valetov (1984; 1992); Valetov et al. (1985) Lesovedeniye (Rus. For. Sci.) No. 3. P. 22-27 & Phytomass and primary production of the marshes with- and without trees. Part 1. Minsk: Berezina Reserve Publ. 218 p. & Primary production structure of swamp forests. Minsk: Uradzhai, 164 p; Valetov et al. (1981; 1985) Soil studies and fertilizer use. Issue 12. Minsk: Uradzhai, P. 11-17 & Primary production structure of swamp forests. Minsk: Uradzhai, 164 p; Valio (2001) Tree Physiol. 21: 65-70; Van Arendonk JJCM. & Villar R. (unpubl.) ; Van Arendonk et al. (1997) Plant Cell Environ. 20: 881-897; Van Cleve (1981) & Barney et al. (1973) In: Dynamics of Forest Ecosystems, pp 648-650 & Can. J. For. Res 3: 304-311; Van Cleve et al. (1971) Arct. Alp. Res. 3: 101-114; Van Hees & Clerkx (2003) For. Ecol. Manage. 176: 439-448; Van Splunder et al. (1996) Can. J. Bot. 74: 1988-1995; Vanninen et al. (1996) Trees 10: 231-238; Vasques et al. (2013) Env. Exp. Bot. 96: 11-19; Vatkovski & Grishina (1971); Vatkovski et al. (1974); Vatkovski (1976) Biological productivity of spruce forests. Tartu: IBP, P. 31-33 & Soils and plant community productivity. Issue 2. Moscow: State University, P. 89-141 & Analysis of forest primary production forming. Moscow: Nauka, 1976. 116 p. ; Vatkovski (1969; 1973) Lesovedeniye (Rus. For. Sci.) No. 1. P. 90-94 & Productivity of organic mass of forests on different natural zones. Moscow: Nauka, P. 28-54 ; Vatkovski (1976) Analysis of forest primary production forming. Moscow: Nauka, 116 p.; Vatkovski (1976); Vatkovski et al. (1975) Analysis

of forest primary production forming. Moscow: Nauka, 116 p. & Geochemical and soil aspects of landscape studies. Moscow: Moscow State University, P. 146-170; Vaz et al. (2012) Trees 26: 1145-1157; Ved' (1978) Lesovedeniye (Rus. For. Sci.) No. 6. P. 63-68; Vedrova (1998) Lesovedeniye (Rus. For. Sci.) No. 6. P. 3-11; Vedrova et al. (2000) Lesovedeniye (Rus. For. Sci.) No. 3. P. 40-48; Vedrova et al. (2002) Lesovedeniye (Rus. For. Sci.) No. 3. P. 40-48; Veenendaal et al. (1996) Funct. Ecol. 10: 501-511; Veneklaas et al. (2002) Sci. Hortic. 93: 75-84; Veselov (1973) Mixed fir and beech forests at the Northern Caucasus and their biological productivity. Krasnodar: Book Publ. House. 211 p.; Villar R. (unpubl.); Villar et al. (2005) Plant Soil 272: 11-27; Villar R. et al. (unpubl.); Vivin & Guehl (1997) Ann. Sci. For. 54: 597-610; Vogel et al. (1997) Plant Ecol. 130: 63-70; Volin & Reich (1996) Physiol. Plant. 97: 674-684; Volin et al. (1998) New Phytol. 138: 315-325; Vomperski & Ivanov (1978) Lesovedenye (Rus. For. Sci.) No. 6. P. 13-24; Vomperski & Ivanov (1982) Biogeocoenologic studies of swamp forests in relation to experimental hydro-melioration. Moscow: Nauka, P. 94-132; Vyskot (1972; 1973a,b) Acta Universitatis Agriculturae (Brno), Series C 41: 243-294 & Lesnictvi 19: 641-658 & Acta Universitatis Agriculturae (Brno), Series C. 42: 215-261; Vyskot (1976) Rozpravy Českoslov. Akad. Ved, Řada Matemat. a Prirodnich Ved. Ročnik 86: 1 –166; Vyskot (1982) Rozpravy Československé Akademie Véd. Rada Matematických a Prirodnich Véd. Praha 92: 1-162; Vznuzdaev et al. (1969; 1971); Kashlev (1968) Complex studying of Populus tremula forests related to its form diversity M.: Nauka, 77 p. & Biological productivity and cycling chemical elements in plant communities. Leningrad: Nauka, P. 53-59 & Reports of Timiryazev Agricult. Academy. Issue 139. P. 249-252; Walters & Reich (1989) Tree Physiol. 5: 159-172; Walters & Reich (1996) Ecology 77: 841-853; Walters et al. (1993) Oecologia 94: 7-16; Wang & Han (2007) Soil Sci. Plant Nutr. 53: 278-285; Wang & Zhang (1992) Larch Forests in China (eds Wang Z., Zhang S.Y.): 45-57 (China Forestry Publishing House, 1992); Wang et al. (1989) For. Sci. Technol.: 11-14 (in Chinese); Wang et al. (1993) J. Liaoning For. Sci. Technol.: 30-34 (in Chinese); Wang et al. (1998) Can. J. For. Res. 28: 44-55; Wang et al. (1998a) J. Fujian Coll. For. 18: 319-323 (in Chinese); Wang et al. (1998b) Hubei For. Sci. Technol.: 16-17 (in Chinese); Wang et al. (1999) J. Beijing For. Univ. 21: 44-49 (in Chinese); Wang et al. (2000) J. Northwest For. Univ. 15: 9-14 (in Chinese); Wang et al. (2001) Glob. Change Biol. 7: 719-730; Wang et al. (2007) For. Sci. Technol. 32: 37-40 (in Chinese); Way & Sage (2008) Glob. Change Biol. 14: 624-636; Way et al. (2013) Plant Cell Environ. 36: 103-115; Wei & Ma (2006a) Acta Agr. Univ. Jiangxi 28: 239-244 (in Chinese); Wei & Ma (2006b) Yantai Norm. Univ. J. (Nat. Sci.) 22: 130-133 (in Chinese); Wei & Ma (2007) J. Northwest Agr. For. Univ. (Nat. Sci.) 35: 171-174 (in Chinese); Wei et al. (1991) J. Guangxi Agr. Coll. 10: 1-26 (in Chinese); Weih et al. (1998) Trees 12: 201-207; Welander & Ottoson (1998) For. Ecol. Manage. 107: 117-126; Wen & Liang (1991) J. Guangxi Agr. Coll. 10: 49-57 (in Chinese); Wen (1993) Long-term Located Research on Forest Ecosystems (eds Liu X.Z., Kang W.X., Chen X.Y., Wen S.Z.): 42-48 (China Forestry Publishing House, 1993); Wen et al. (1988) J. Guangxi Agr. Coll. 7: 55-66 (in Chinese); Wen et al. (1997) Sci. Silvae Sin. 33(S2): 181-188 (in Chinese); Wen et al. (2000) J. Guangxi Agr. Biol. Sci. 19: 1-5 (in Chinese); Wendler & Millard (1996) Tree Physiol. 16: 153-159; Westman & Rogers (1977) Aust. J. Bot. 25: 171-191; Wiebel et al. (1994) Tree Physiol. 14: 263-274; Will & Teskey (1997) Tree Physiol. 17: 655-661; Witkowski (1991) Funct. Ecol. 5: 101-110; Wong et al. (1992) Aust. J. Bot. 40: 457-472; Wright & Westoby (1999) J. Ecol. 87: 85-97; Wu & Dang (1992a) J. Yunnan Univ. (Nat. Sci.) 14: 119-127 (in Chinese); Wu & Dang (1992b) J. Yunnan Univ. (Nat. Sci.) 14: 137-145 (in Chinese); Wu (1995) J. Fujian For. Sci. Technol. 22: 63-65 (in Chinese); Wu (2005a) J. Fujian For. Sci. Technol. 32: 125-129 (in Chinese); Wu (2005b) J.

Fujian Coll. For. 25: 142-146 (in Chinese); Wu et al. (1991) J. Fujian Coll. For. 11: 19-25 (in Chinese); Wu et al. (1994) J. Anhui Agr. Univ.: 20-26 (in Chinese); Wu et al. (1994a) J. Yunnan Univ. (Nat. Sci.) 16: 230-234 (in Chinese); Wu et al. (1994b) J. Yunnan Univ. (Nat. Sci.) 16: 220-224 (in Chinese); Wu et al. (1994c) J. Yunnan Univ. (Nat. Sci.) 16: 245-249 (in Chinese); Wu et al. (1996) Prot. For. Sci. Technol.: 21-24 (in Chinese); Wu et al. (1997) J. Liaoning For. Sci. Technol.: 28-31 (in Chinese); Wu et al. (1999) J. Fujian For. Sci. Technol. 26: 18-21 (in Chinese); Wu et al. (2003) J. Anhui Agr. Univ.: 20-26 (in Chinese); Xiao (1988) Acta Biol. Plateau Sin. 8: 147-157 (in Chinese); Xiao (1990) Acta Phytoecol. Geobot. Sin. 14: 237-246 (in Chinese); Xiao et al. (2000) J. Zhejiang For. Sci. Technol. 20: 26-30 (in Chinese); Xiao et al. (2003) Tree Physiol. 23: 505-519; Xiao et al. (2005) Trees 19: 711-720; Xie & Yang (2002) J. Nanjing For. Univ. (Nat. Sci.) 26: 49-52 (in Chinese); Xie (2004) Prot. For. Sci. Technol.: 23-25 (in Chinese); Xie et al. (2001a) J. Zhejiang For. Coll. 18: 354-358 (in Chinese); Xie et al. (2001b) J. Soil Water Conserv. 15: 66-70 (in Chinese); Xie et al. (2005) J. Sichuan For. Sci. Technol. 26: 1-6 (in Chinese); Xu & Chen (1990) Trop. Subtrop. For. Ecosys. 7: 148-157 (in Chinese); Xu et al. (1988) J. Ecol. No. 7 (Suppl.). P. 49-59; Xu et al. (1998) China For. Sci. Technol.: 35-36 (in Chinese); Xu et al. (2004) For. Res. 17: 26-35 (in Chinese); Xu et al. (2005) J. Northeast For. Univ. 33: 29-32 (in Chinese); Xu et al. (2012) Env. Exp. Bot. 77: 1-11; Xue & Luo (2002) J. South China Agr. Univ. 23: 24-26 (in Chinese); Xue & Sheng (1993) For. Sci. Technol.: 16-19 (in Chinese); Yakimchuk & Hoddinott (1994) Can. J. For. Res. 24: 1-8; Yamada & Shidei (1968) From: Cannell (1982); Yamakura et al. (1972a,b) From: Cannell (1982); Yamomoto et al. (1987) Can. J. For. Res. 17: 69-79; Yan & Zhu (1984) Collect. Guizhou Agr. Coll.: 101-105 (in Chinese); Yan et al. (1995) Prot. For. Sci. Technol.: 13-17 (in Chinese); Yan et al. (1997) J. Beijing For. Univ. 19(S2): 108-112 (in Chinese); Yang & Wu (2005) For. Sci. Technol. 30: 14-15 (in Chinese); Yang & Yang (2004) J. Northwest Norm. Univ. (Nat. Sci.) 40: 70-75 (in Chinese); Yang et al. (1987) Sichuan For. Sci. Technol. 8: 21-24 (in Chinese); Yang et al. (1991) J. Fujian Coll. For. 11: 341-348 (in Chinese); Yang et al. (1996) J. Fujian Coll. For. 16: 200-204 (in Chinese); Yang et al. (1999) J. Northeast For. Univ. 27: 9-12 (in Chinese); Yang et al. (2000) Sci. Silvae Sin. 36(S1): 120-124 (in Chinese); Yang et al. (2001) J. Mt. Sci. 19: 503-510 (in Chinese); Yang et al. (2004) Acta Agr. Univ. Jiangxi 26: 164-168 (in Chinese); Yang et al. (2005) For. Ecol. Man. 216: 216-226; Yang et al. (2005) Physiol. Plant. 124: 431-440; Yang et al. (2007) Jiangxi For. Sci. Technol.: 8-10 (in Chinese); Yang et al. (2008) J. Cent. South Univ. For. Technol. 28: 122-126 (in Chinese); Yang et al. (2008) J. Plant Res. 121: 377-385; Yang et al. (2013) J. Plant Growth Regul. 32: 298-306; Yao & Li (1997) Sci. Silvae Sin. 33(S2): 203-207 (in Chinese); Yao (1989) J. Beijing For. Univ. 11: 38-46 (in Chinese); Yao (2003) China For. Sci. Technol. 17: 13-15 (in Chinese); Yao et al. (1998) J. Shanxi Agr. Univ. 18: 310-315 (in Chinese); Yao et al. (2003) J. Cent. South For. Univ. 23: 1-5 (in Chinese); Yarmishko & Demianov (1983); Deyeva (1985; 1987) Botan. Zhurnal (Rus. Bot. J.) 68: 1225-1235 & 70: 54-58 & 72: 505-511; Yashcheritsina (1981a,b) Ph. D. Thesis. Sverdlovsk: Forest Engineering Institute, 24 p. & Stability of pine plantations on sandy soils of dry steppe related to their phytomass dynamics: Dissertation. Sverdlovsk: Forest Engineering Institute, 206 p. (USFEU library); Yazaki et al. (2004) Tree Physiol. 24: 941-949; Ye & Jiang (1983) Acta Ecol. Sin. 3: 7-14 (in Chinese); Ye et al. (1984) J. Nanjing Inst. For.: 1-9 (in Chinese); Ye et al. (1996) Prot. For. Sci. Technol. (S): 17-20 (in Chinese); Ye et al. (2007) J. Nanjing For. Univ. (Nat. Sci.) 31: 43-46 (in Chinese); Yi et al. (2008) J. Cent. South Univ. For. Technol. 28: 50-53 (in Chinese); Yin et al. (2005) Physiol. Plant. 123: 445-451; Ying et al. (2001) J. Fujian Coll. For. 21: 339-342 (in Chinese); Yoda (1967; 1968) J. Coll. Arts Sci. Chiba Univ. : 99-140 & 6: 277-302.; Yu &

Jiang (1992) Proceeding of Forest Ecosystems on Xiashu Ecological Station (eds Jiang Z.L.): 78-89 (China Forestry Publishing House, 1992) (in Chinese); Yu & Ong (2001) Photosynthetica 39: 477-479; Yu et al. (1990) Trop. Subtrop. For. Ecosys. 6: 145-152 (in Chinese); Yu et al. (2005) Chinese J. Appl. Ecol. 15: 1837-1841 (in Chinese); Yuan et al. (1998) Yunnan For. Sci. Technol.: 24-27 (in Chinese); Yuan et al. (2002) J. Northeast For. Univ. 30: 5-7 (in Chinese); Yurkevich & Yaroshevich (1974) Biological productivity of types and associations of pine forests. Minsk: Nauka i Tekhnica, 294 p.; Yurkevich et al. (1971; 1975) Types and associations of spruce forests (Studies in Byelorussian Republic). Minsk: Nauka i Tekhnika, 351 p.; Yuste et al. (2005) Tree Physiol. 25: 701-712; Zaboyeva et al. (1973); Archegova et al. (1975) Rastitelnye resursy (Plant Resources) 9: 100-106 & Productivity and elements cycling in northern phytocoenoses. Leningrad: Nauka, 130 p.; Zak et al. (1993) Plant Soil 151: 105-117; Zak et al. (2000) Ecol. Appl. 10: 34-46; Zavala & Ravetta (2002) Plant Ecol. 161: 185-191; Zavitkovski & Stevens (1972) Ecol. 53: 235-242; Zavitkovski & Stevens (1972) Ecol. 53: 235-243; Zavitkovski & Stevens (1972) Ecol. 53: 235-244; Zavitkovski & Stevens (1972) Ecol. 53: 235-245; Zekri (1991) Sci. Hortic. 47: 305-315; Zeng (2007) J. Mt. Agr. Biol. 26: 211-214 (in Chinese); Zeng et al. (1995) Studies on the Eucalypts Ecosystem of Short Rotation in Leizhou (eds Zeng, T.X.): 21-34 (China Forestry Publishing House, 1995) (in Chinese); Zeng et al. (2013) New For. 44: 703-718; Zhai (1982ab) J. Beijing For. Coll.: 1-11 (in Chinese); J. Beijing For. Coll.: 67-79 (in Chinese); Zhai et al. (1992) J. Beijing For. Univ. 14(S1): 156-163 (in Chinese); Zhang & Chen (1992) Shaanxi For. Sci. Technol.: 13-17 (in Chinese); Zhang & Feng (1979) Hubei For. Sci. Technol.: 1-7 (in Chinese); Zhang & Shangguan (1992) J. Shanxi Univ. 15: 72-77; Zhang & Shangguan (2006) Acta Ecol. Sin. 26: 373-382 (in Chinese); Zhang & Yuan (1988) Acta Phytoecol. Geobot. Sin. 12: 63-69 (in Chinese); Zhang (1981) J. Northeast. For. Inst.: 85-98 (in Chinese); Zhang (1990) J. Northwest. Coll. For. 5: 1-7 (in Chinese); Zhang (1992) J. Northwest For. Coll. 7: 64-67 (in Chinese); Zhang (2000) For. Sci. Technol.: 7-10 (in Chinese); Zhang (2006) J. Fujian Norm. Univ. (Nat. Sci.) 22: 102-106 (in Chinese); Zhang et al. (1984) J. Northeast. For. Inst. 12: 1-6 (in Chinese); Zhang et al. (1992) J. Fujian For. Sci. Technol. 19: 29-33 (in Chinese); Zhang et al. (1999) J. Northeast For. Univ. 27: 31-35 (in Chinese); Zhang et al. (2004) Plant Sci. 166: 791-797; Zhang et al. (2006) J. Agr. Univ. Hebei 29: 37-43 (in Chinese); Zhang et al. (2007) J. Nanjing For. Univ. (Nat. Sci.) 31: 143-146 (in Chinese); Zhang et al. (2009) Tree Physiol. 29: 1307-1316; Zhang et al. (2013a) Photosynthetica 50: 67-76; Zhang et al. (2013b) For. Ecol. Manage. 306: 234-242; Zhang et al. (2014) Plant Sci. 226: 182-188; Zhao & Chen (1994) Intensive Cultivations of Poplars in China (eds Zhao T.X., Chen Z.J.): 521-522 (China Science and Technology Press, 1994); Zhao & Tian (2000) J. Cent. South For. Univ. 20: 7-13 (in Chinese); Zhao (2006) Jiangxi For. Sci. Technol.: 5-7, 59 (in Chinese); Zhao et al. (1999) Acta Agr. Univ. Henan 33: 350-353 (in Chinese); Zhao et al. (2006) J. Beijing For. Univ. 28: 39-44 (in Chinese); Zheleznikov (1973; 1981a,b,c) Problems of raising forest productivity on the Far East. Blagoveshchensk: Agricultural Institute, P. 9-12 & Ph. D. Thesis. Sverdlovsk: ULTI, 22 p. (USFEU library) & Dissertation. Ussuriysk: Agricultural Institute, 226 p. & Ecology of Quercus mongolica at Primorski territory. Vladivostok: Far Eastern Sci. Centre AS USSR. P. 21-29.; Zheng & Chen (1999) J. Trop. Subtrop. Bot. 7: 282-288 (in Chinese); Zheng (1986) For. Invent. Plan. Northwest. North. China : 16-20 (in Chinese); Zheng (1996) J. Fujian Coll. For. 16: 114-118 (in Chinese); Zheng (1998) China For. Sci. Technol.: 30-31 (in Chinese); Zheng et al. (2002) Scand. J. For. Res. 17: 35-46; Zheng et al. (2005) J. Fujian Norm. Univ. (Nat. Sci.) 21: 65-69 (in Chinese); Zheng et al. (2009) Plant Ecol. 203: 263-271; Zhilkin (1966) The ways of increasing forest productivity. Minsk: Vysshaya Shkola, P 37-54; Zhilkin et al. (1971) Forestry

and Forest Management (Minsk). Issue 4. P. 13-21.; Zhong et al. (2008) J. Subtrop. Resour. Environ. 3: 11-18 (in Chinese); Zhou & Huang (1991) Acta Phytoecol. Geobot. Sin. 15: 9-16 (in Chinese); Zhou (1999) J. Fujian Coll. For. 19, 146-148 (in Chinese); Zhou (2004) J. Fujian Coll. For. 24: 68-71 (in Chinese); Zhou et al. (1993) Sichuan For. Sci. Technol. 14: 15-25 (in Chinese); Zhou et al. (1997) For. Res. 10: 453-457 (in Chinese); Zhu & Yang (1979) J. Guizhou Agr. Coll.: 1-14 (in Chinese); Zhu et al. (1993) Sci. Technol. Qinghai Agr. For.: 15-20 (in Chinese); Zhu et al. (2002) J. Nanjing For. Univ. (Nat. Sci.) 26: 15-20 (in Chinese); Zhu et al. (2006) Acta Agr. Univ. Jiangxi 28: 90-94 (in Chinese); Zhuravleva (1974) Biological productivity of vegetation of Kazakhstan. Alma-Ata: Nauka, P. 214-233; Ziska et al. (1991) Oecologia 86: 383-389; Zolotokrylin & Nosova (1974); Dylis & Nosova (1977) Lesovedeniye (Rus. For. Sci.). No. 4. P. 24-32 & Phytomass of forest biogeocoenoses on localities near Moscow. Moscow: Nauka, 143 p.; Zou et al. (1995) Chinese J. Appl. Ecol. 6: 123-127 (in Chinese); Zou et al. (2007) Funct. Ecol. 21: 721-730; Zribi et al. (2009) Sci. Hortic. 120: 367-372; Zubov (1977) Transactions Far Eastern For. Manage. Inst. Issue 19. Khabarovsk, P. 75-80; Zurbriggen et al. (2013) Plant Ecol. 214: 385-396; Zyabchenko & Ivanchikov (1975; 1978) Problems of forestry and forest management in Karelia. Petrozavodsk: Karelian Branch AS USSR. P. 38-50 & Growth and productivity of pine forests at Karelian republic and Murmansk oblast'. Petrozavodsk: Forest Institute of Karelian Branch of AS USSR, P. 30-75; Zyabchenko & Ivanchikov (1978) Growth and productivity of pine forests at Karelian republic and Murmansk oblast'. Petrozavodsk: Forest Institute of Karelian Branch of AS USSR, P. 30-75;

Table S3 Overview of the representation of various plant groups in the database. Data are provided for the total number of observations for herbaceous and woody species, the % of the total observations related to each phylogenetic class, the % of all observations of a phylogenetic class that were made in the field (rather than in a growth chamber, a glasshouse, an Open Top Chamber or in an experimental field), and the total number of species represented by the observations in a phylogenetic class.

Functional Type	# of observations in the database	Phylogenetic class	% of total observations	% of the # of observations made in the field	# of species per phylogenetic class
Herbaceous	2954	Monocots	12.2	0	192
		Eudicots	14.2	0	210
Woody	8178	Gymnosperms	33.4	80	108
		Basal Angiosperms	0.8	54	23
		Monocots	1.2	55	14
		Eudicots	37.7	35	643
Intermediate	69	Eudicots	0.6	1	15

Table S4 Allometric scaling exponents as given for all records of all species, and for herbaceous and woody species separately. (a) Standard Major Axis regression (SMA; model 2 regression) for the intercept (α) and slope (β) of the regression of leaf mass (LM) vs. root mass (RM), stem mass (SM) vs. root mass, and leaf mass vs. stem mass, all based on log10-transformed values. The 95% confidence interval for the slope, as well as the r2 of the equation are given. (b) Ordinary least square regression (OLS), tested for linear and quadratic relationships. a is the value for the intercept, b1, b2 and b3 are the values for the linear, the quadratic and the cubic component, respectively. The degrees of freedom was >3000 for herbaceous species and >8,200 for woody species.

(A) Species	Regression	α	β	95% CI for β	r^2
All	LM vs. SM	0.113	0.740	0.738 - 0.742	0.978
	LM vs. RM	0.070	0.849	0.847 - 0.851	0.977
	SM vs. RM	-0.058	1.147	1.145 - 1.149	0.988
Herbaceous	LM vs. SM	0.239	0.864	0.855 - 0.872	0.928
	LM vs. RM	0.192	0.985	0.972 - 0.991	0.931
	SM vs. RM	-0.054	1.140	1.128 - 1.153	0.904
Woody	LM vs. SM	0.127	0.728	0.725 - 0.730	0.971
	LM vs. RM	0.077	0.834	0.831 - 0.837	0.970
	SM vs. RM	-0.069	1.146	1.143 - 1.149	0.987

(B) Species	Regression	a	b_1	b_2	b_3	r^2
All	LM vs. SM	0.213	0.795	-0.0177	-	0.981
	LM vs. RM	0.151	0.897	-0.0184	-	0.979
	SM vs. RM	-0.126	1.144	0.0318	-0.00606	0.989
Herbaceous	LM vs. SM	0.216	0.819	-0.0082		0.926
	LM vs. RM	0.173	0.918	-0.0219		0.931
	SM vs. RM	-0.102	1.081	-		0.901
Woody	LM vs. SM	0.225	0.790	-0.0221	0.00094	0.977
	LM vs. RM	0.110	0.897		-0.00356	0.975
	SM vs. RM	-0.147	1.138	0.0408	-0.00748	0.990
